# Supplementary material for: Diels-Alder reaction affords circumpyrene tetracarboxydiimide with excited state intramolecular charge transfer character
Source: Commun Chem. 2026 Feb 28;9:122. doi: 10.1038/s42004-026-01946-3 (PMC12992557; doi:10.1038/s42004-026-01946-3)
Supplement: Supplementary file 2 — Supporting Information [file 42004_2026_1946_MOESM2_ESM.pdf]

Supporting Information for

Diels-Alder reaction affords circumpyrene tetracarboxydiimide with excited state intramolecular charge transfer character

Qiang Chen, Michele Guizzardi, Francisco Méndez, Giulio Cerullo, Chengwei Ju, Silvio Osella, Helena Rose Keller, Francesco Scotognella, Giuseppe M. Paternò,* Klaus Müllen, and Akimitsu Narita**

Table of Contents

1. Supplementary Methods S2
2. Synthetic Details S2
3. Theoretical Calculations S5
4. Supplementary Figures S10
5. MALDI-TOF MS Spectra S15
6. Supplementary References S16
7. Calculated Molecular Cartesian Coordinates S18

1. Supplementary Methods

All reactions working with air- or moisture-sensitive compounds were carried out under argon atmosphere using standard Schlenk line techniques. Unless otherwise noted, all starting materials were purchased from commercial sources and used without further purification. All other reagents were used as received. Thin layer chromatography (TLC) was done on silica gel coated aluminum sheets with F254 indicator and column chromatography separation was performed with silica gel (particle size 0.063 – 0.200 mm). Nuclear Magnetic Resonance (NMR) spectra were recorded using Bruker DPX 400, Bruker DPX 500 MHz NMR spectrometers. Chemical shifts (δ) were expressed in ppm relative to the residual of solvents (Chloroform-*d*, ^{1}H: 7.26 ppm, ^{13}C: 77.16 ppm; 1,1,2,2-tetrachloroethane-d_2, ^{1}H: 6.00 ppm, ^{13}C: 73.78 ppm). Abbreviations: s = singlet, d = doublet, t = triplet, q = quartet, m = multiplet. Coupling constants (J) were reported in Hertz. High resolution mass spectra (HR MS) were recorded on a G6545A Q-ToF (Agilent GmbH, Waldbronn, Germany) with atmospheric pressure chemical ionization (APCI) mass spectrometer or on a SYNAPT G2-Si high definition Q-TOF mass spectrometer (Waters Corp., Manchester, UK) by matrix-assisted laser desorption/ionization (MALDI) using 7,7,8,8-tetracyanoquinodimethane (TCNQ) as matrix and calibrated with poly(ethylene glycol). Data processing was done with MassLynx software V4.1. UV-*vis* absorption spectra were recorded on a Perkin-Elmer Lambda 900 spectrometer at room temperature using a 10 mm quartz cell. Photoluminescence spectra were recorded on a J&M TIDAS spectrofluorometer. Absolute fluorescence quantum yields were measured using an integrating sphere. For transient absorption (TA) spectroscopy, the molecule was dissolved in toluene with a concentration of 0.1 mg mL^{-1}. We employed an amplified Ti:sapphire laser with 2 mJ output energy, 1 kHz repetition rate, and a central energy of 1.59 eV (800 nm). We used a pump wavelength 620 nm, which is resonant with the main $\pi \rightarrow \pi^*$ transition, generated by using a visible optical parameter amplifier (OPA). Pump pulses were focused on a 200 µm spot (diameter), keeping pump fluence at ~ 50 µJ cm^{-2}. As a probe pulse, we used a broadband white light super-continuum generated in sapphire plate in the spectral region from 440 nm to 750 nm. Cyclic voltammetry (CV) measurements were performed on a computer-controlled GSTAT-12 in a three-electrode cell in anhydrous *o*-dichlorobenzene solution of *n*-Bu_4NPF_6 (0.1 M) at a scan rate of 50 mV/s at room temperature. A silver wire, a Pt wire and a glassy carbon electrode were used as the reference electrode, the counter electrode, and the working electrode, respectively.

2. Synthetic Details

The synthesis of **DBOV-Mes 1** was described in the literature.[1]

Synthesis of *N*-hexylmaleimide (**2a**)[2]

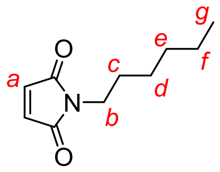

To a solution of maleic anhydride (1.47 g, 15.0 mmol) dissolved in acetic acid (10 mL) was added dropwise a solution of *n*-hexylamine (1.52 g, 15.0 mmol) in acetic acid (10 mL) at room temperature under inert atmosphere. After addition, the mixture was heated at 120 °C for 16 h. The solvent was removed under reduced pressure after completion of the reaction. The residue was purified by silica gel column chromatography (eluent: *n*-hexane:ethyl acetate = 4:1, v/v) to give the title compound (1.5 g, 55% yield) as colorless oil. ^{1}H NMR (400 MHz, $CDCl_3$, 298 K) δ 6.67 (s, 2H, H^{a}), 3.50 (t, J = 7.4 Hz, 2H, , H^{b}), 1.60 – 1.49 (m, 2H, H^{c}), 1.34 – 1.20 (m, 6H, H^{a} – H^{f}), 0.86 (t, J = 7.0 Hz, 3H, H^{f}); ^{13}C NMR

(100 MHz, $CDCl_3$, 298 K) δ 171.0 ($\underline{C}$=O), 134.2 (C^a), 38.1 (C^b), 31.4 (C^c), 28.6 (C^d), 26.5 (C^e), 22.6 (C^f), 14.1 (C^g); HRMS (APCI) [M^+] Calculated for $C_{10}H_{15}NO_2$: 181.1097; Found 181.1106.

Synthesis of **3a** and **4a**

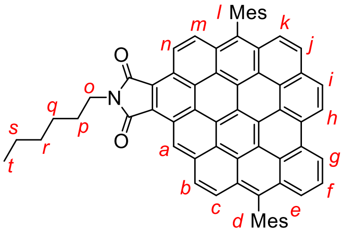

3a

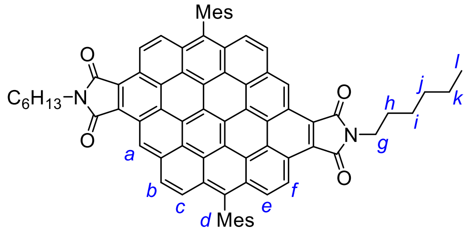

4a

To a 25-mL Schlenk tube was added **DBOV-Mes 1** (20 mg, 0.029 mmol), *N*-*n*-hexylmaleimide (**2a**) (639 mg, 3.53 mmol) and diphenyl ether (2.0 mL). The mixture was degassed by three freeze-pump-thaw cycles and heated at 265 °C for 70 h. After cooling down to a room temperature, the mixture was purified by silica gel column chromatography (eluent: tetrahydrofuran:*n*-hexane = 1:100 to 1:10, v/v) and size-exclusion chromatography (Bio-beads S-X3, eluent: toluene) to give mono-adduct **3a** (4.2 mg, 16% yield) and di-adduct **4a** (5.6 mg, 18% yield). **3a**: Brown solid. ^{1}H NMR (500 MHz, $C_2D_2Cl_4$, 413 K) δ 10.70 (s, 1H, H^a), 10.23 (d, J = 8.4 Hz, 1H, H^h), 10.12 (d, J = 9.0 Hz, 1H, H^n), 9.95 (d, J = 6.8 Hz, 1H, H^g), 9.28 (d, J = 8.3 Hz, 1H, H^i), 8.92 (d, J = 9.0 Hz, 1H, H^j), 8.87 (d, J = 9.2 Hz, 1H, H^m), 8.66 (d, J = 9.0 Hz, 1H, H^k), 8.61 (d, J = 9.3 Hz, 1H, H^b), 8.57 – 8.46 (q, J = 7.7 Hz, 2H, $H^{e,f}$), 8.14 (d, J = 9.3 Hz, 1H, H^c), 7.40 (s, 2H, H^d or H^l), 7.35 (s, 2H, H^l or H^d), 4.09 (t, J = 7.3 Hz, 2H, N-$\underline{CH_2}$-), 2.69 (s, 3H, Mes-$\underline{CH_3}$), 2.65 (s, 3H, Mes-$\underline{CH_3}$), 2.15 – 2.02 (m, 8H, Ar-$\underline{CH_3}$ and N-CH_2-$\underline{CH_2}$-) 1.99 (s, 6H, Ar-$\underline{CH_3}$), 1.65 (p, J = 8.1, 7.5 Hz, 2H, -$\underline{CH_2}$-CH_2-CH_2-CH_3), 1.57 – 1.50 (m, 2H, -$\underline{CH_2}$-CH_2-CH_3), 1.49 – 1.43 (m, 2H, -$\underline{CH_2}$-CH_3), 0.99 (t, J = 7.2 Hz, 3H, -$\underline{CH_3}$); ^{13}C NMR (125 MHz, $C_2D_2Cl_4$, 413 K) δ 137.93, 137.88, 137.77, 137.70, 135.89, 134.86, 134.67, 134.39, 133.22, 132.17, 131.71, 131.05, 130.81, 130.29, 129.88, 129.23, 128.67, 128.64, 128.60, 128.49, 128.38, 127.79, 126.79, 126.51, 126.12, 125.74, 123.88, 123.40, 122.91, 122.61, 121.94, 121.71, 121.67, 121.16, 38.31, 31.22, 29.31, 28.72, 26.56, 21.01, 20.97, 19.93, 19.84, 13.43; HRMS (MALDI-TOF) [M^+] Calculated for $C_{66}H_{47}NO_2$: 885.3602; Found 885.3596; UV-vis (toluene): λ (ε) = 380 (3.31×10^4 $M^{-1}cm^{-1}$), 451 (3.56×10^4 $M^{-1}cm^{-1}$), 512 (2.36×10^4 $M^{-1}cm^{-1}$), 548 (5.02×10^4 $M^{-1}cm^{-1}$), 609 (1.87×10^4 $M^{-1}cm^{-1}$). **4a**: Brown solid. ^{1}H NMR (500 MHz, $C_2D_2Cl_4$, 413 K) δ 11.63 (s, 2H, H^a), 10.84 (d, J = 9.0 Hz, 2H, H^f), 9.63 (d, J = 9.0 Hz, 2H, H^e), 9.54 (d, J = 9.3 Hz, 2H, H^b), 9.14 (d, J = 9.2 Hz, 2H, H^c), 7.57 (s, 4H, H^d), 4.25 (t, J = 7.3 Hz, 4H, N-$\underline{CH_2}$-), 2.82 (s, 6H, Mes-$\underline{CH_3}$), 2.20 (p, J = 7.4 Hz, 4H, N-CH_2-$\underline{CH_2}$-), 2.11 (s, 12H, Mes-$\underline{CH_3}$), 1.80 – 1.72 (m, 4H N-CH_2-CH_2-$\underline{CH_2}$-), 1.65 – 1.59 (m, 4H, -$\underline{CH_2}$-CH_2-CH_3), 1.58 – 1.51 (m, 4H, -$\underline{CH_2}$-CH_3), 1.06 (t, J = 7.2 Hz, 6H, -$\underline{CH_3}$); Solubility of **4a** was too low to record a reasonable ^{13}C NMR spectrum; HRMS (MALDI-TOF) [M^+] Calculated for $C_{76}H_{58}N_2O_4$: 1062.4392; Found 1062.4401; UV-vis (toluene): λ (ε) = 448 (8.13×10^4 $M^{-1}cm^{-1}$), 504 (2.06×10^4 $M^{-1}cm^{-1}$), 541 (5.32×10^4 $M^{-1}cm^{-1}$), 579 (1.85×10^4 $M^{-1}cm^{-1}$), 628 (4.16×10^4 $M^{-1}cm^{-1}$).

Synthesis of *N*-(4-*tert*-butylbenzyl)maleimide (**2b**)

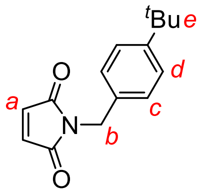

To a solution of maleic anhydride (2.00 g, 20.4 mmol) dissolved in acetic acid (10 mL) was added dropwise a solution of 4-*tert*-butylbenzylamine (3.33 g, 20.4 mmol) in acetic acid (10 mL) at room temperature under inert atmosphere. After addition, the mixture was heated at reflux for 10 h. The solvent was removed under reduced pressure after completion of the reaction. The residue was purified by silica gel column chromatography (eluent: *n*-hexane:ethyl acetate = 5:1, v/v) to give the title

compound (2.5 g, 50% yield) as a light-yellow solid. ^{1}H NMR (250 MHz, CD_2Cl_2) δ 7.35 (d, J = 8.3 Hz, 2H, H^c), 7.23 (d, J = 8.4 Hz, 2H, H^d), 6.70 (s, 2H, H^a), 4.62 (s, 2H, H^b), 1.29 (s, 9H, H^e); ^{13}C NMR (63 MHz, CD_2Cl_2) δ 171.0, 151.2, 134.6, 134.0, 128.2, 125.9, 41.3, 34.8, 31.5; HRMS (APCI) [M^+] Calculated for $C_{15}H_{17}NO_2$: 243.1252; Found 243.1261.

Synthesis of **3b** and **4b**

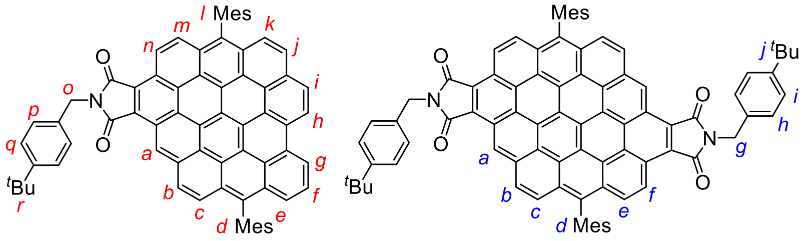

To a 25-mL Schlenk tube was added **DBOV-Mes 1** (50 mg, 0.073 mmol), *N*-(4-*tert*-butylbenzyl)maleimide (**2b**) (2.14 g, 8.80 mmol) and diphenyl ether (2.0 mL). The mixture was degassed by three freeze-pump-thaw cycles and heated at 265 °C for 47 h. After cooling down to a room temperature, the mixture was purified by silica gel column chromatography (eluent: tetrahydrofuran:*n*-hexane = 1:100 to 1:10, v/v) and size-exclusion chromatography (Bio-beads S-X3, eluent: toluene) to give mono-adduct **3b** (6.3 mg, 9% yield) and di-adduct **4b** (3.1 mg, 4% yield). **3b**: Brown solid. ^{1}H NMR (500 MHz, $C_2D_2Cl_4$, 413 K) δ 10.73 (s, 1H, H^a), 10.29 (d, J = 8.3 Hz, 1H, H^h), 10.16 (d, J = 9.1 Hz, 1H, H^n), 10.00 (d, J = 6.7 Hz, 1H, H^g), 9.34 (d, J = 8.2 Hz, 1H, H^i), 8.97 (d, J = 9.2 Hz, 1H, H^j), 8.92 (d, J = 9.2 Hz, 1H, H^m), 8.71 (d, J = 9.2 Hz, 1H, , H^k), 8.65 (d, J = 9.4 Hz, 1H, H^b), 8.58 (s, 2H, H^e and H^f), 8.19 (d, J = 9.3 Hz, 1H, H^c), 7.67 (d, J = 8.0 Hz, 2H, H^p), 7.48 (d, J = 8.2 Hz, 2H, H^q), 7.45 (s, 2H, H^d or H^l), 7.39 (s, 2H, H^l or H^d), 5.28 (s, 2H, H^o), 2.73 (s, 3H, Mes-CH_3), 2.69 (s, 3H, Mes-CH_3), 2.13 (s, 6H, Mes-$(CH_3)_2$), 2.03 (s, 6H, Mes-$(CH_3)_2$), 1.39 (s, 9H, -^{t}Bu); ^{13}C NMR (126 MHz, $C_2D_2Cl_4$, 413 K) δ 170.1, 169.9, 137.9, 137.8, 137.7, 137.4, 134.8, 134.6, 133.3, 130.0, 129.9, 129.2, 128.7, 128.6, 128.5, 127.9, 126.9, 126.6, 126.1, 125.8, 125.4, 123.8, 123.4, 121.9, 121.8, 121.7, 121.1, 113.5, 41.4, 31.1, 26.6, 21.1, 21.0, 20.0, 19.9; HRMS (MALDI-TOF) [M^+] Calculated for $C_{71}H_{49}NO_2$: 947.3756; Found 947.3764; UV-vis (toluene): λ (ε) = 379 (3.73 × 10^4 $M^{-1}cm^{-1}$), 407 (2.44 × 10^4 $M^{-1}cm^{-1}$), 423 (2.50 × 10^4 $M^{-1}cm^{-1}$), 452 (4.06 × 10^4 $M^{-1}cm^{-1}$), 478 (9.27 × 10^3 $M^{-1}cm^{-1}$), 512 (2.77 × 10^4 $M^{-1}cm^{-1}$), 549 (5.80 × 10^4 $M^{-1}cm^{-1}$), 611 (2.28 × 10^4 $M^{-1}cm^{-1}$). **4b**: Brown solid. ^{1}H NMR (500 MHz, $C_2D_2Cl_4$, 413 K) δ 11.65 (s, 2H, H^a), 10.85 (d, J = 9.2 Hz, 2H, H^f), 9.64 (d, J = 8.8 Hz, 2H, H^e), 9.55 (d, J = 7.6 Hz, 2H, H^b), 9.14 (d, J = 9.2 Hz, 2H, H^c), 7.77 (d, J = 7.6 Hz, 4H, H^h), 7.56 (s, 4H, H^d), 7.52 (d, J = 9.5 Hz, 4H, H^i), 5.42 (s, 4H, H^g), 2.81 (s, 6H, Mes-CH_3), 2.10 (s, 12H, Mes-$(CH_3)_2$), 1.41 (s, 18H, -^{t}Bu); Solubility of **4b** was too low to record a reasonable ^{13}C NMR spectrum; HRMS (MALDI-TOF) [M^+] Calculated for $C_{86}H_{62}N_2O_4$: 1186.4705; Found 1186.4690; UV-vis (toluene): λ (ε) = 357 (1.62 × 10^4 $M^{-1}cm^{-1}$), 407 (4.64 × 10^4 $M^{-1}cm^{-1}$), 421 (4.34 × 10^4 $M^{-1}cm^{-1}$), 449 (7.28 × 10^4 $M^{-1}cm^{-1}$), 504 (1.92 × 10^4 $M^{-1}cm^{-1}$), 542 (4.78 × 10^4 $M^{-1}cm^{-1}$), 582 (1.56 × 10^4 $M^{-1}cm^{-1}$), 630 (3.68 × 10^4 $M^{-1}cm^{-1}$).

Synthesis of *N*-(4-*tert*-butylphenyl)maleimide (**2c**)

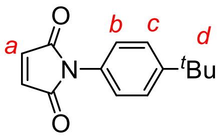

The reaction mixture of maleic anhydride (1.96 g, 20.0 mmol) and 4-*tert*-butylaniline (2.98 g, 20.0 mmol) dissolved in dichloromethane (50 mL) was stirred at 25 °C for 1 h. After evaporation of the solvent under reduced pressure, the residue was dried under high vacuum for 0.5 h. The crude product was dissolved in acetic anhydride (30 mL), and sodium acetate (1.00 g, 12.2 mmol) was added to the mixture, which was then heated at 100 °C for 3 h. After cooling down to a room temperature, the mixture was poured

into 450 mL of water and extracted with ethyl acetate (300 mL). The organic layer was separated, washed with brine, dried over Na_2SO_4, and evaporated. The residue was purified by silica gel column chromatography (eluent: *n*-hexane:ethyl acetate = 10:1, v/v) to give the title compound (3.26 g, 71% yield) as a light-yellow solid. ^{1}H NMR (300 MHz, CD_2Cl_2, 298 K) δ 7.51 (d, J = 8.8 Hz, 2H, H^b), 7.23 (d, J = 8.7 Hz, 2H, H^c), 6.83 (s, 2H, H^a), 1.35 (s, 9H, H^d); ^{13}C NMR (75 MHz, CD_2Cl_2, 298 K) δ 170.2, 151.7, 134.6, 129.1, 126.5, 126.4, 35.0, 31.4; HRMS (APCI) [M^+] Calculated for $C_{14}H_{15}NO_2$: 229.1098; Found 229.1102.

Synthesis of **3c**

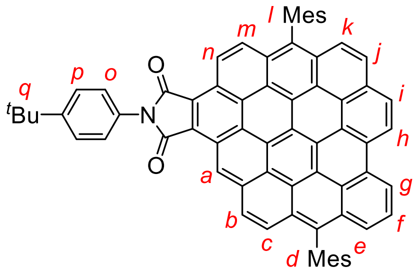

To a 25-mL Schlenk tube was added **DBOV-Mes 1** (10 mg, 15 μmol), *N*-(4-*tert*-butylphenyl) maleimide (**2c**) (404 mg, 1.76 mmol) and diphenyl ether (0.5 mL). The mixture was degassed by three freeze-pump-thaw cycles and heated at 265 °C for 48 h. After cooling down to a room temperature, the mixture was purified by silica gel column chromatography (eluent: *n*-hexane to tetrahydrofuran:*n*-hexane = 1:100 to 1:10, v/v) and recrystallized from dichloromethane and methanol to give mono-adduct **3c** (3.2 mg, 23% yield) as a dark brown solid. ^{1}H NMR (500 MHz, $C_2D_2Cl_4$, 413 K) δ 10.76 (s, 1H, H^a), 10.25 (d, J = 8.5 Hz, 1H, H^h), 10.18 (d, J = 9.3 Hz, 1H, H^n), 9.96 (d, J = 6.1 Hz, 1H, H^g), 9.30 (d, J = 8.4 Hz, 1H, H^i), 8.97 – 8.89 (m, 2H, H^j / H^m), 8.68 (d, J = 9.0 Hz, 1H, H^k), 8.62 (d, J = 9.5 Hz, 1H, H^b), 8.59 – 8.52 (m, 2H, H^e / H^f), 8.16 (d, J = 9.3 Hz, 1H, H^c), 7.78 (d, J = 8.5 Hz, 2H, H^o), 7.70 (d, J = 6.6 Hz, 2H, H^p), 7.41 (s, 2H, H^d or H^l), 7.35 (s, 2H, H^l / H^d), 2.69 (s, 3H, Mes-$\underline{CH_3}$), 2.65 (s, 3H, Mes-$\underline{CH_3}$), 2.09 (s, 6H, Mes-($\underline{CH_3}$)$_2$), 2.00 (s, 6H, Mes-($\underline{CH_3}$)$_2$), 1.52 (s, 9H, H^q); ^{13}C NMR (126 MHz, $C_2D_2Cl_4$, 413 K) δ 169.9, 169.7, 138.2, 138.0, 137.8, 136.3, 135.1, 134.9, 133.7, 131.3(3), 131.3(1), 131.1, 130.3, 130.2, 129.5, 129.0, 128.9, 128.8, 128.3, 127.4, 127.1, 126.83, 126.75, 126.4, 126.1, 126.0, 124.24, 124.15, 123.9, 123.7, 123.6, 123.4, 122.4, 122.3, 122.1, 122.0, 121.5, 121.4, 121.0, 120.6, 31.4, 29.6, 21.2, 21.2, 20.2, 20.1; HRMS (MALDI-TOF) [M^+] Calculated for $C_{70}H_{47}NO_2$: 933.3602; Found 933.3585; UV-vis (toluene): λ (ε) = 381 (4.93 × 10^4 $M^{-1}cm^{-1}$), 412 (2.94 × 10^4 $M^{-1}cm^{-1}$), 424 (3.17 × 10^4 $M^{-1}cm^{-1}$), 454 (4.43 × 10^4 $M^{-1}cm^{-1}$), 479 (1.23 × 10^3 $M^{-1}cm^{-1}$), 514 (3.47 × 10^4 $M^{-1}cm^{-1}$), 551 (7.32 × 10^4 $M^{-1}cm^{-1}$), 613 (2.83 × 10^4 $M^{-1}cm^{-1}$).

3. Theoretical Calculations

The activation energy for **Figure S1** was calculated in gas phase at the B3LYP/6-31G(d,p)//B3LYP/6-31G(d,p) level of theory using Gaussian 09 program (revision B.01)[3]. Frequency calculations confirmed that the local minimum and transition states have 0 and 1 imaginary frequencies, respectively.

Diels-Alder cycloaddition energy profiles for **Figure 3** and **Figures S2-S4** were performed using Gaussian 09 program (revision B.01)[3] at the B3LYP/6-31G(d)//B3LYP/6-31G(d), M062X/6-31G(d)//B3LYP/6-31G(d) and wB97XD/6-31G(d)//B3LYP/6-31G(d) levels of theory using diphenyl ether as solvent with the polarizable continuum model (PCM), respectively, unless otherwise noted. The nature of the transition states were verified through calculation of the intrinsic reaction coordinate using the Gonzalez-Schlegel's method implemented in Gaussian 09.[3] The molecular orbitals and energy levels of **3a** and **4a** shown in **Figures 4d** and **S5** were calculated at the B3LYP/6-31G(d,p) level of theory (*n*-hexyl group was replaced by methyl) using Gaussian 09 program.[4]

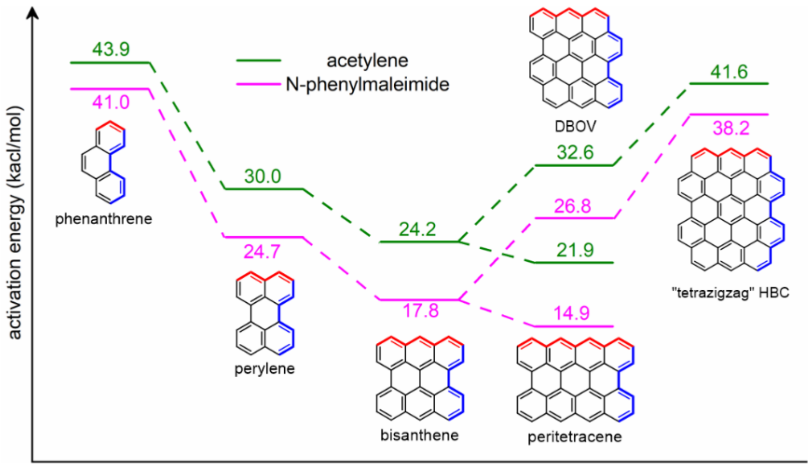

Figure S1. Activation energy, calculated in gas phase at the B3LYP/6-31G(d,p)//B3LYP/6-31G(d,p) level of theory using Gaussian 09 program (revision B.01), for the Diels-Alder addition of the first bay region of PAHs with acetylene (green) and *N*-phenylmaleimide (magenta).

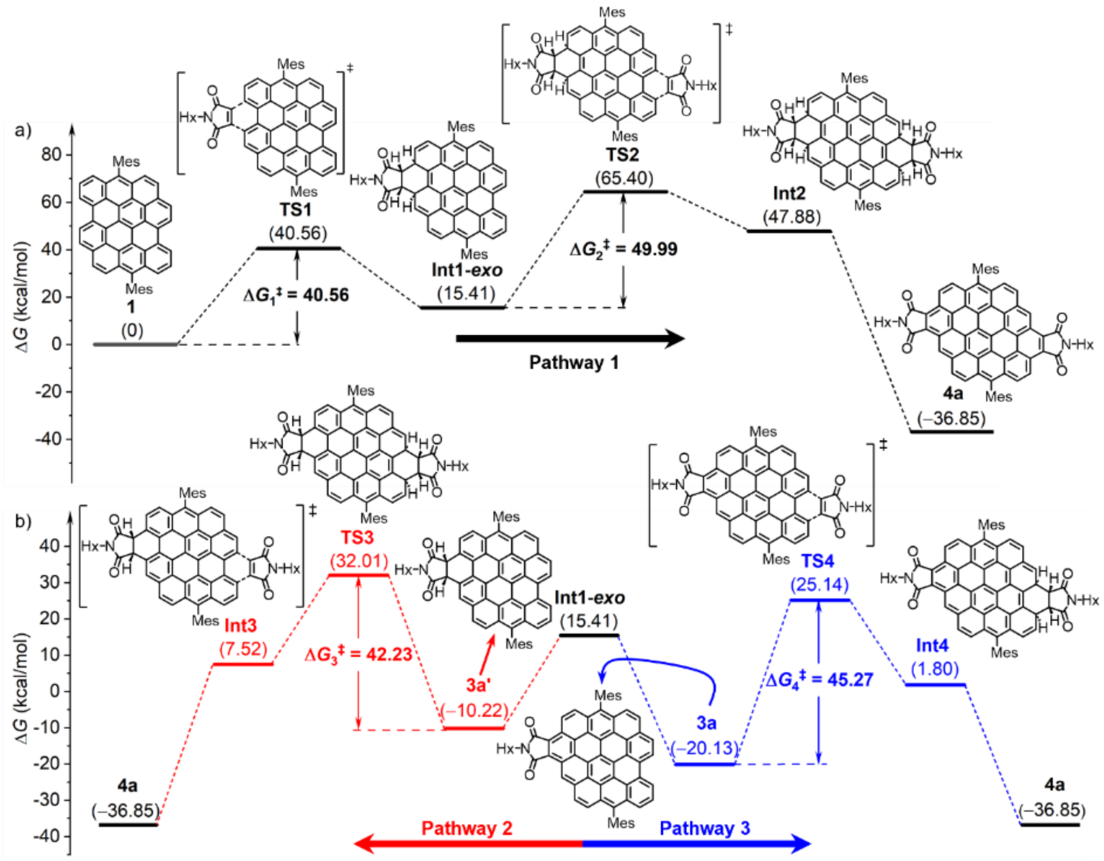

Figure S2. The relative free energy profiles for the D-A addition of DBOV-Mes (**1**) with N-hexyl maleimide (**2a**) in diphenyl ether via (a) pathway 1 through Int1-exo and (b) pathway 2/3 involving partially (**3a'**) and fully aromatized mono-adduct (**3a**). They were calculated at the B3LYP/6-31G(d)//B3LYP/6-31G(d)level of theory using the polarizable continuum model (PCM) with Gaussian 09 program (revision B.01).

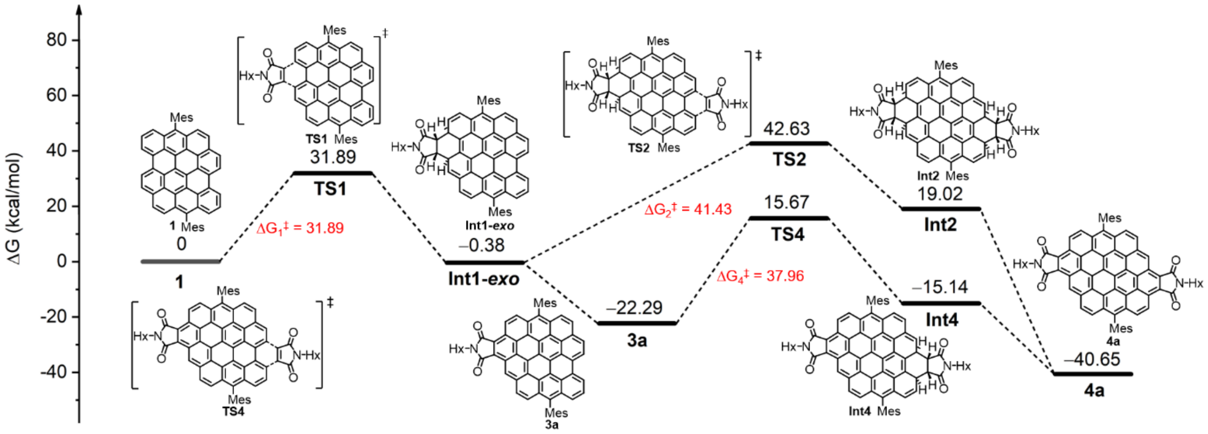

Figure S3. Energy diagram of Diels-Alder addition of DBOV-Mes (**1**) with *N*-*n*-hexylmaleimide (**2a**) in diphenyl ether calculated at the M062X/6-31G(d)//B3LYP/6-31G(d) level of theory using the polarizable continuum model (PCM) with Gaussian 09 program (revision B.01).

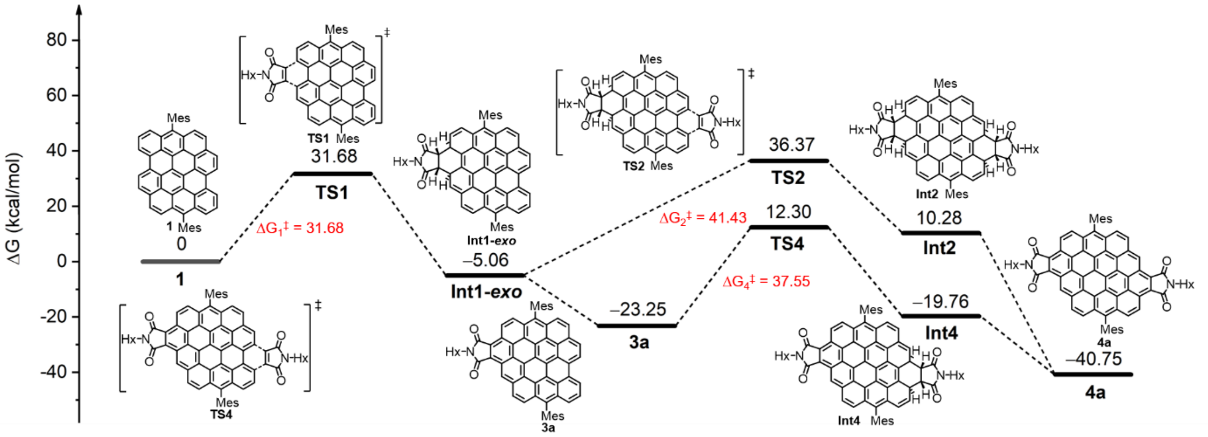

Figure S4. Energy diagram of Diels-Alder addition of DBOV-Mes (**1**) with *N*-*n*-hexylmaleimide (**2a**) in diphenyl ether calculated at the wB97XD/6-31G(d)//B3LYP/6-31G(d) level of theory using the polarizable continuum model (PCM) with Gaussian 09 program (revision B.01).

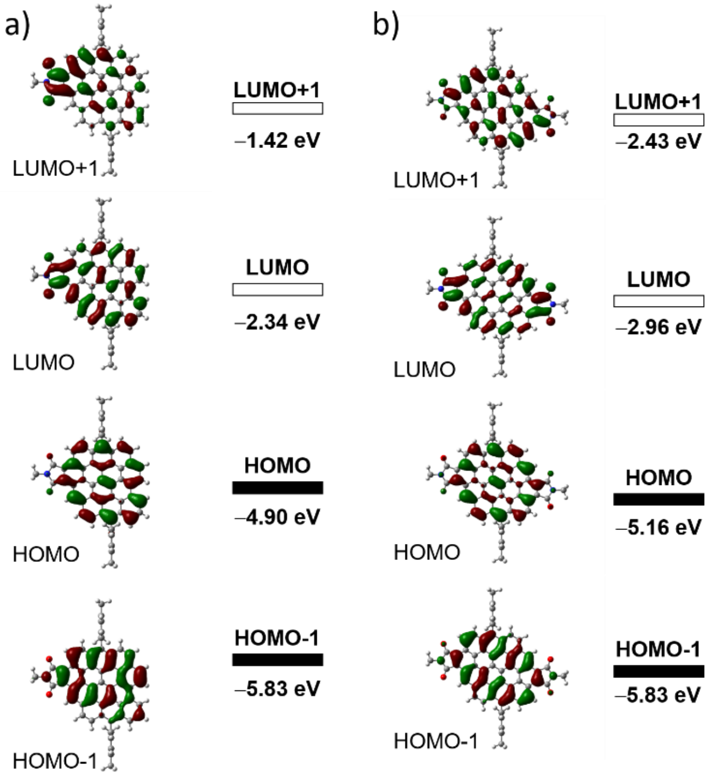

Figure S5. The molecular orbitals and energy levels of **3a** and **4a** calculated at the DFT (B3LYP/6-31G(d,p)) level of theory (*n*-hexyl group was replaced by methyl).

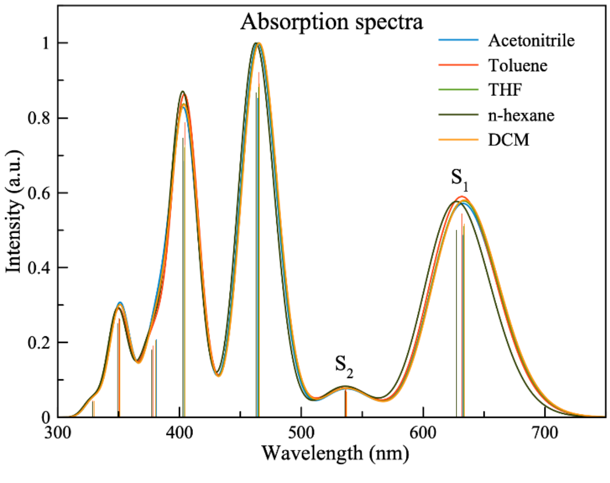

Figure S6. Simulated UV-vis absorption of **4a** in acetonitrile, toluene, THF, *n*-hexane and DCM, respectively by employing the implicit universal solvation model based on solute electron density (SMD). The electronic excited states were computed with the time-dependent density functional theory (TD-DFT)[5] framework with the HSE functional and 6-31G(d,p) basis set on all atoms. The electronic vertical excitation energies (VEEs) were calculated with the first thirty singlet states for the **4a** molecule taken into consideration. All calculations were performed for solvated systems, in toluene, THF, *n*-hexane, DCM and acetonitrile, by employing the implicit universal solvation model based on solute electron density (SMD)[6] and using the Gaussian16 package.[7] Before the TD-DFT

calculation, the geometry of the single molecule was fully optimized without any symmetry constraints, within the same level of theory and with implicit solvent accounted for. The excited states properties were analyzed with the Multiwnf software.[8]

Table S1. Excited state analysis of **4a** in different solvents.

Toluene	Wavelength (nm)	Energy (eV)	Oscillator strength	transition
S1	631.86	1.96	0.544	H → L (92%)
S2	536.64	2.31	0.076	H-1 → L (42%)<br>H → L+1 (54%)
S4	465.25	2.66	0.923	H-1 → L (58%)<br>H → L+1 (42%)
S9	404.33	3.07	0.788	H-1 → L+1 (88%)
S14	378.22	3.28	0.191	H-2 → L+2 (90%)

THF	Wavelength (nm)	Energy (eV)	Oscillator strength	transition
S1	633.30	1.96	0.511	H → L (92%)
S2	537.11	2.31	0.069	H-1 → L (43%)<br>H → L+1 (53%)
S5	465.01	2.66	0.883	H-1 → L (56%)<br>H → L+1 (44%)
S11	404.13	3.07	0.788	H-1 → L+1 (86%)
S14	380.39	3.26	0.205	H-2 → L+2 (88%)

n-hexane	Wavelength (nm)	Energy (eV)	Oscillator strength	transition
S1	627.45	1.98	0.501	H → L (91%)
S2	535.95	2.31	0.072	H-1 → L (42%)<br>H → L+1 (54%)
S5	462.66	2.68	0.828	H-1 → L (57%)<br>H → L+1 (43%)
S9	402.94	3.08	0.747	H-1 → L+1 (87%)
S15	377.05	3.29	0.180	H-2 → L+2 (89%)

DCM	Wavelength (nm)	Energy (eV)	Oscillator strength	transition
S1	634.06	1.96	0.518	H → L (92%)
S2	537.24	2.31	0.069	H-1 → L (43%)<br>H → L+1 53%)
S5	465.45	2.66	0.892	H-1 → L (56%)<br>H → L+1 (44%)

S11	404.35	3.07	0.728	H-1 → L+1 (86%)
S14	380.61	3.26	0.207	H-2 → L+2 (88%)

Acetonitrile	Wavelength (nm)	Energy (eV)	Oscillator strength	transition
S1	632.85	1.96	0.487	H → L (92%)
S2	537.16	2.31	0.065	H-1 → L (44%)<br>H→ L+1 (52%)
S5	464.30	2.67	0.853	H-1 → L (56%)<br>H→ L+1 (44%)
S11	403.75	3.07	0.685	H-1 → L+1 (84%)
S14	380.99	3.25	0.209	H-2 → L+2 (87%)

4. Supplementary Figures

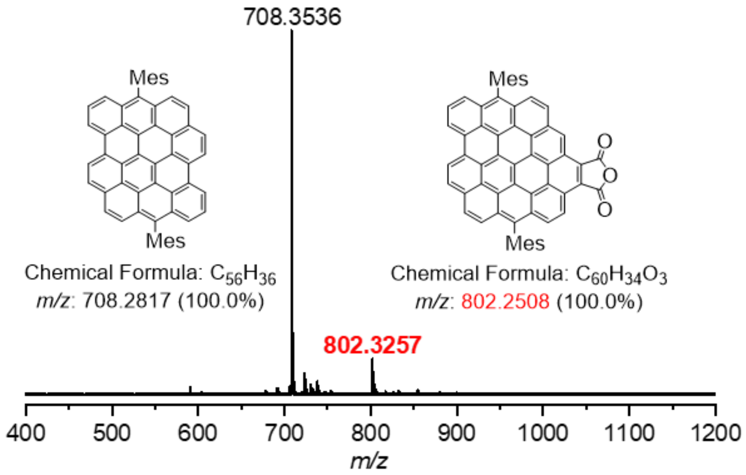

Figure S7. MALDI-TOF MS spectrum of the reaction mixture of DBOV-Mes **1** with maleic anhydride (100 eq.) at 260 °C for 48 h, showing the formation of trace mono-adduct with *m/z* = 802.3257.

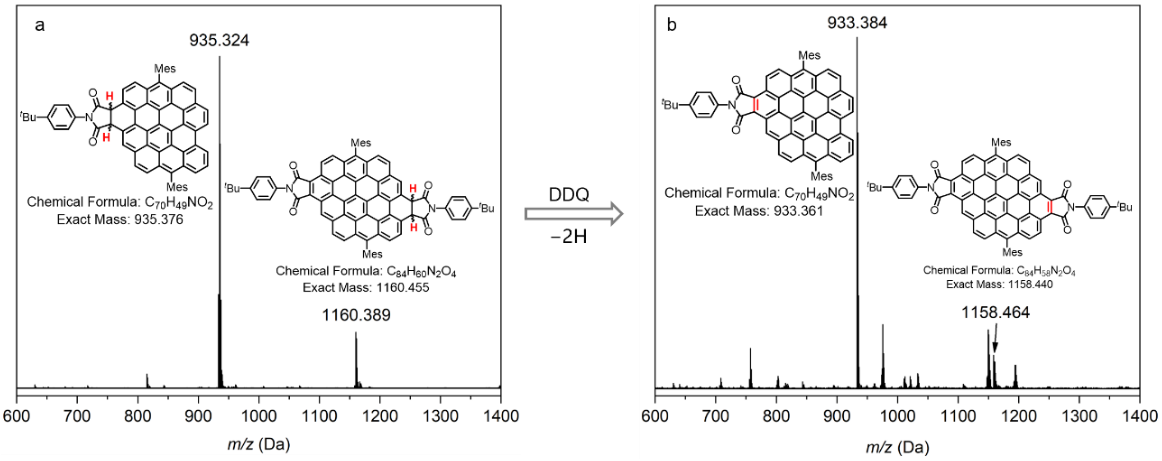

Figure S8. a) MALDI-TOF MS spectrum showing the formation of partially dehydrogenated D-A adducts for the reaction of DBOV-Mes and *N*-(*tert*-butylbenzyl) maleimide (**2c**); b) MALDI-TOF MS spectrum measured after further dehydrogenation of the intermediates in a) by DDQ using toluene as solvent (*c* = 0.2 mmol/L).

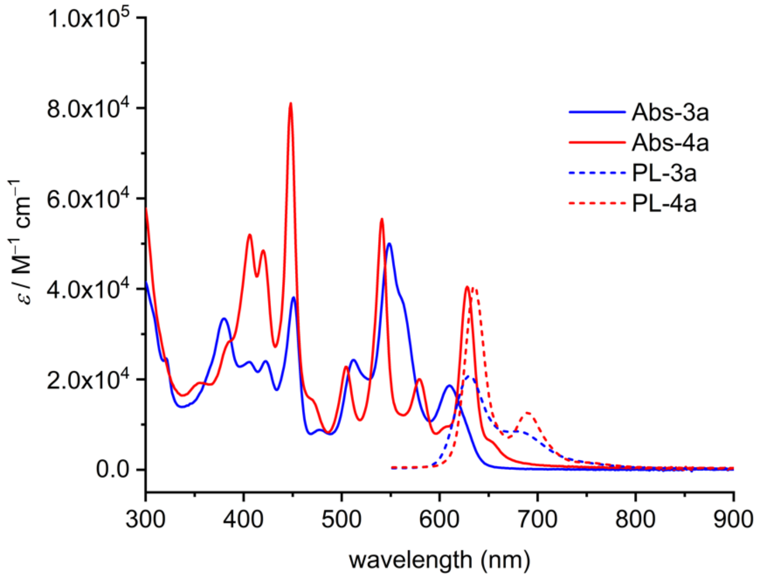

Figure S9. UV-vis absorption and fluorescence spectra of **3a** (c = 1.27 × 10^{-5} M) and **4a** (c = 1.07 × 10^{-5} M) measured in toluene solution at 25 °C. For fluorescence spectra measurement, the excitation wavelengths were 548 nm and 448 nm, respectively.

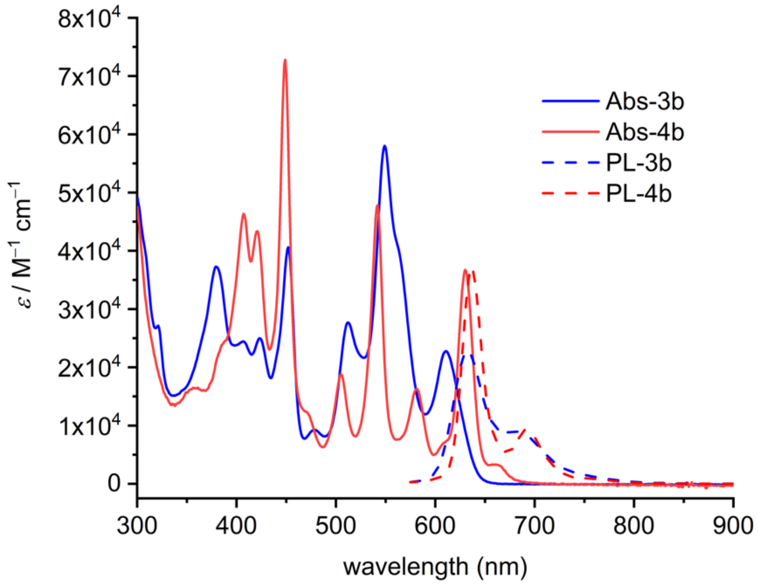

Figure S10. UV-vis absorption and fluorescence spectra of **3b** (c = 1.35 × 10^{-5} M) and **4b** (c = 1.35 × 10^{-5} M) measured in toluene at 25 °C. For fluorescence spectra measurement, the excitation wavelengths were 512 nm and 505 nm, respectively.

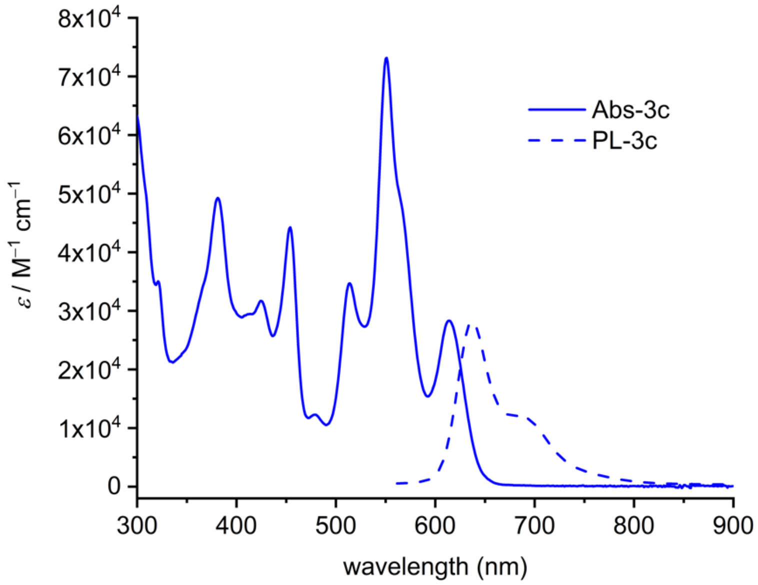

Figure S11. UV-vis absorption and fluorescence spectra of **3c** (c = 1.14 × 10^{-5} M) measured in toluene solution at 25 °C. For fluorescence spectrum measurement, the excitation wavelength was 513 nm.

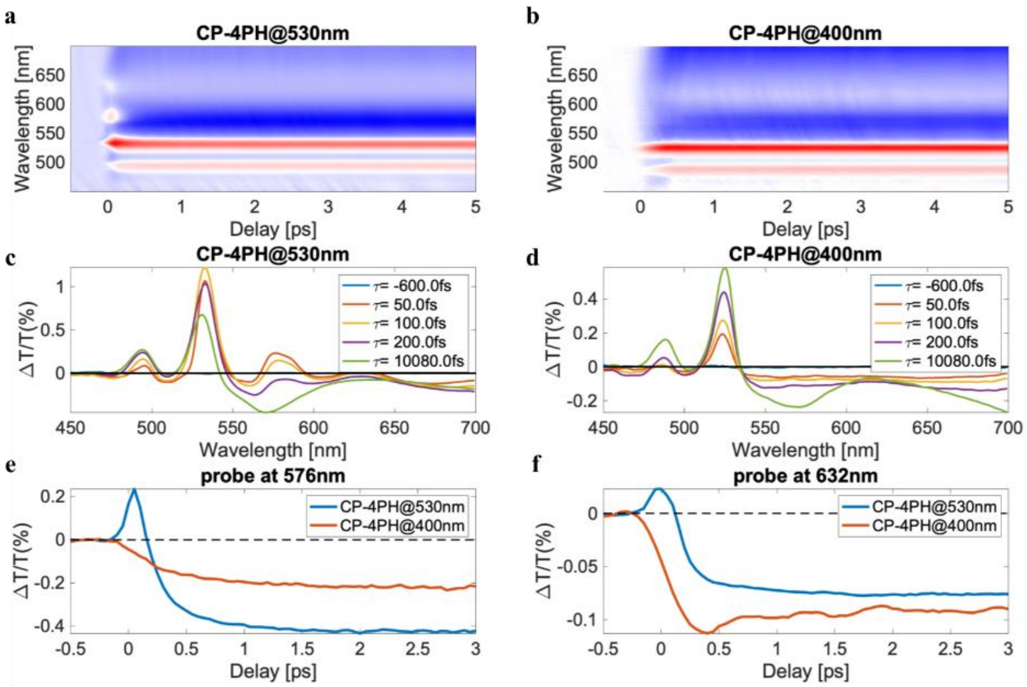

Figure S12. Transient absorption spectroscopy of **CP-Ph** (in the figure as CP-4PH since it bears four phenyl groups). The sample was dissolved in toluene at a concentration of 0.1 mg/mL, to ensure comparable absorption and transient absorption signal to **4a**. The sample in solution was excited both at 530 nm (main $\pi \rightarrow \pi^*$ transition) and higher lying states at 400 nm. Transient absorption maps obtained by exciting at a) 530 nm and b) 400 nm, respectively; Transient absorption spectra at selected pump-probe delays obtained by exciting at c) 530 nm and d) 400 nm, respectively; Dynamical traces at probe wavelengths of e) 576 nm (excitation wavelengths at 400 nm) and f) 632 nm (excitation wavelengths at 530 nm), respectively. We attribute the positive signals at 576 nm and 632 nm to stimulated emission from S_1. For this molecule, we observe an ultrafast quenching of the stimulated emission (below 0.5 ps), in contrast with the long stimulated emission of molecule **4a.**

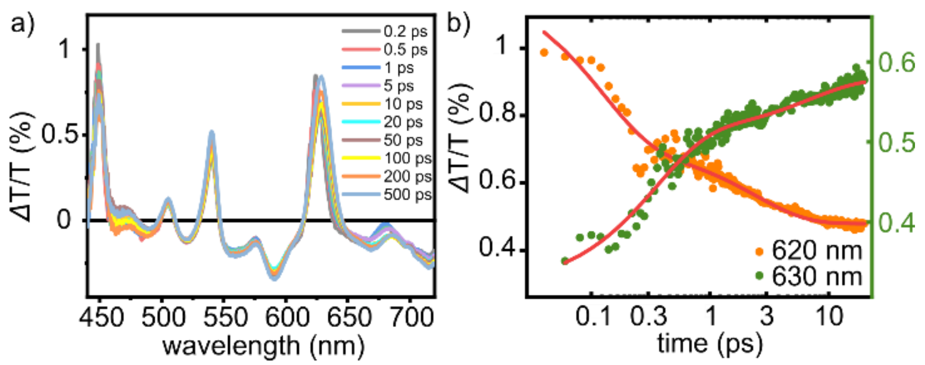

Figure S13. a) Transient absorption spectra of **4a** upon excitation at 620 nm (toluene, 0.1 mg/mL); b) Dynamical traces of the photobleaching (620 nm) and stimulated emission (630 nm) signals. The red curves represent the bi-exponential fitting model.

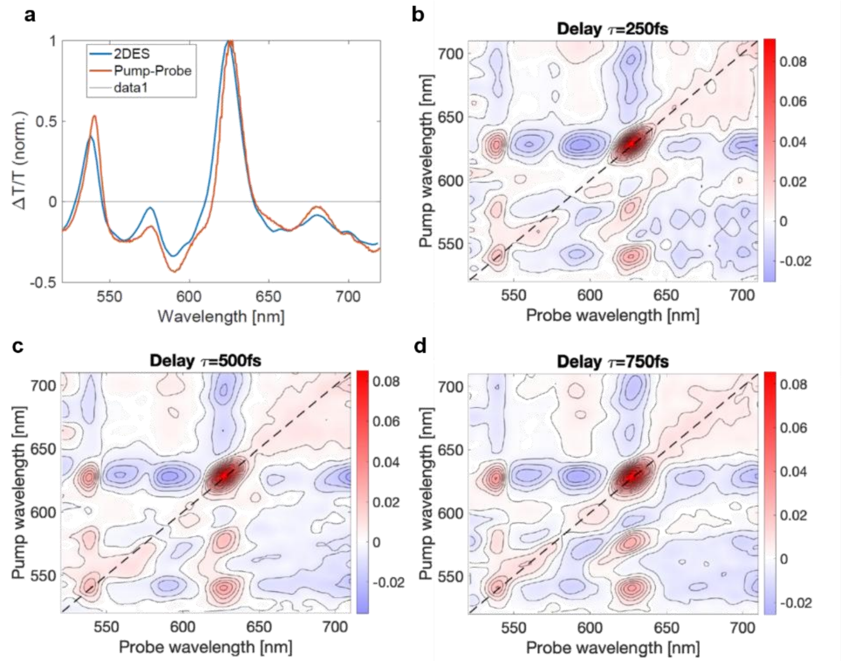

Figure S14. a) Transient absorption spectra of **4a** upon excitation at 630 nm (toluene, 0.1 mg/mL) in comparison with a horizontal slice of the 2DES map at 630 nm; b-d) Two-dimensional electronic spectroscopy map taken at different delay (τ = 250 fs, 500 fs, 750 fs) between pump and probe.

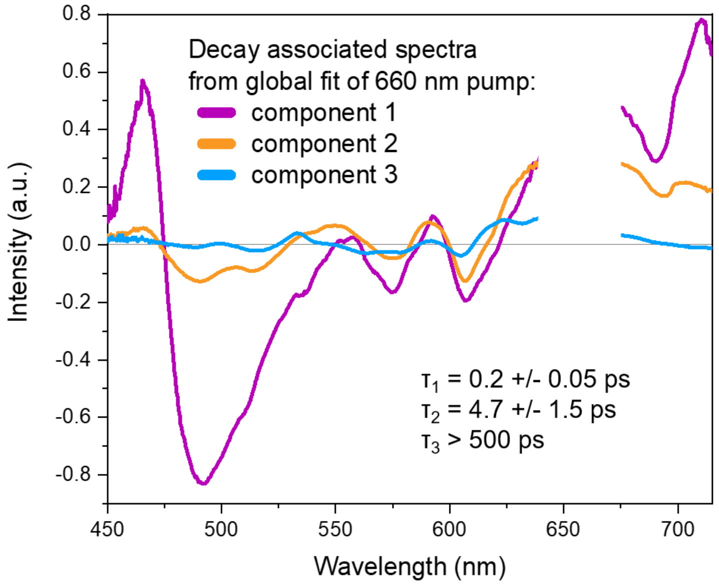

Figure S15. Decay associated spectra obtained from global fitting analysis of the transient absorption spectra for **4a** pumped at 660 nm.

5. MALDI-TOF MS Spectra

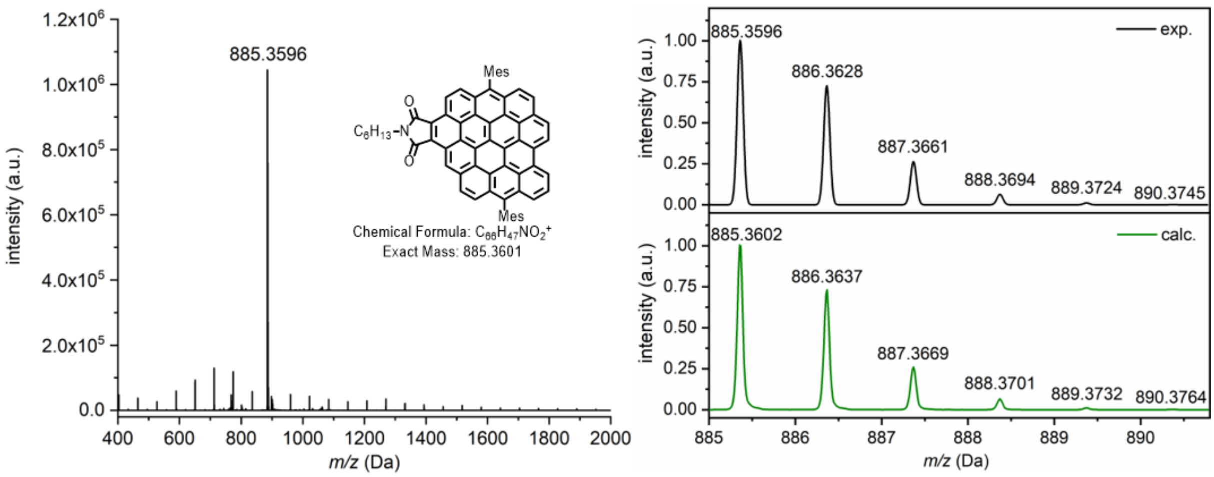

Figure S16. MALDI-TOF MS (TCNQ as matrix) spectrum of mono-adduct **3a**, right: comparison of experimental isotropic distribution pattern (top) with simulation (bottom).

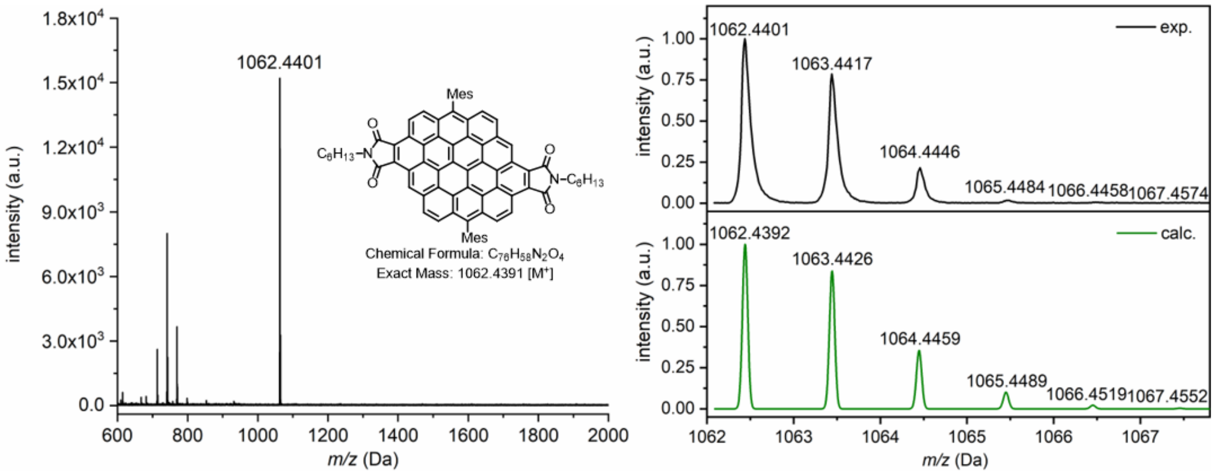

Figure S17. MALDI-TOF MS (TCNQ as matrix) spectrum of di-adduct **4a**, right: comparison of experimental isotropic distribution pattern (top) with simulation (bottom).

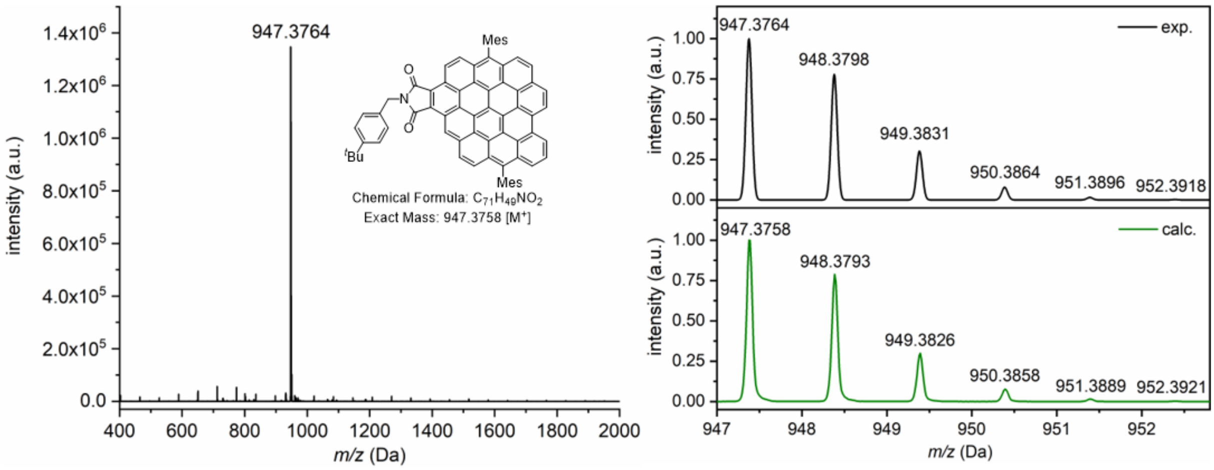

Figure S18. MALDI-TOF MS (TCNQ as matrix) spectrum of mono-adduct **3b**, right: comparison of experimental isotropic distribution pattern (top) with simulation (bottom).

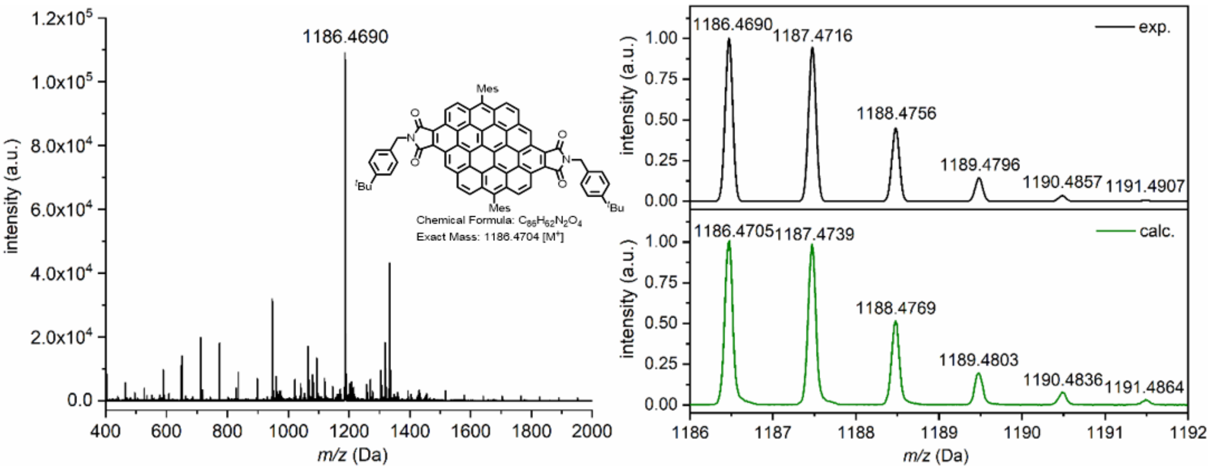

Figure S19. MALDI-TOF MS (TCNQ as matrix) spectrum of di-adduct **4b**, right: comparison of experimental isotropic distribution pattern (top) with simulation (bottom).

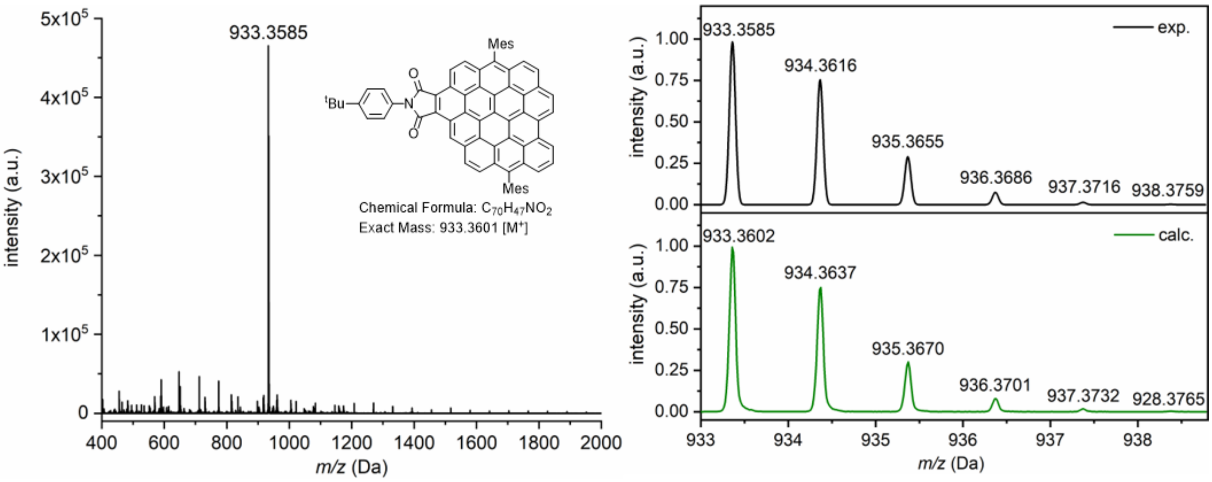

Figure S20. MALDI-TOF MS (TCNQ as matrix) spectrum of mono-adduct **3c**, right: comparison of experimental isotropic distribution pattern (top) with simulation (bottom).

6. Supplementary References

(1) Chen, Q.; Thoms, S.; Stottinger, S.; Schollmeyer, D.; Müllen, K.; Narita, A.; Basche, T., Dibenzo[hi,st]ovalene as Highly Luminescent Nanographene: Efficient Synthesis via Photochemical Cyclodehydroiodination, Optoelectronic Properties, and Single-Molecule Spectroscopy. *J. Am. Chem. Soc.* **2019,** *141* (41), 16439-16449.

(2) Khayum, M. A.; Kandambeth, S.; Mitra, S.; Nair, S. B.; Das, A.; Nagane, S. S.; Mukherjee, R.; Banerjee, R., Chemically Delaminated Free-Standing Ultrathin Covalent Organic Nanosheets. *Angew. Chem. Int. Ed.* **2016,** *55* (50), 15604-15608.

(3) M. J. Frisch, G. W. T., H. B. Schlegel, G. E. Scuseria, M. A. Robb, J. R. Cheeseman, G. Scalmani, V. Barone, B. Mennucci, G. A. Petersson, H. Nakatsuji, M. Caricato, X. Li, H. P. Hratchian, A. F. Izmaylov, J. Bloino, G. Zheng, J. L. Sonnenberg, M. Hada, M. Ehara, K. Toyota, R. Fukuda, J. Hasegawa, M. Ishida, T. Nakajima, Y. Honda, O. Kitao, H. Nakai, T. Vreven, J. A. Montgomery, Jr., J. E. Peralta, F. Ogliaro, M. Bearpark, J. J. Heyd, E. Brothers, K. N. Kudin, V. N. Staroverov, T. Keith, R. Kobayashi, J. Normand, K. Raghavachari, A. Rendell, J. C. Burant, S. S. Iyengar, J. Tomasi,

M. Cossi, N. Rega, J. M. Millam, M. Klene, J. E. Knox, J. B. Cross, V. Bakken, C. Adamo, J. Jaramillo, R. Gomperts, R. E. Stratmann, O. Yazyev, A. J. Austin, R. Cammi, C. Pomelli, J. W. Ochterski, R. L. Martin, K. Morokuma, V. G. Zakrzewski, G. A. Voth, P. Salvador, J. J. Dannenberg, S. Dapprich, A. D. Daniels, O. Farkas, J. B. Foresman, J. V. Ortiz, J. Cioslowski, and D. J. Fox *Gaussian 09, Revision B.01,* 2010, Gaussian, Inc.: Wallingford CT.

(4) M. J. Frisch, G. W. Trucks, H. B. Schlegel, G. E. Scuseria, M. A. Robb, J. R. Cheeseman, G. Scalmani, V. Barone, B. Mennucci, G. A. Petersson, H. Nakatsuji, M. Caricato, X. Li, H. P. Hratchian, A. F. Izmaylov, J. Bloino, G. Zheng, J. L. Sonnenberg, M. Hada, M. Ehara, K. Toyota, R. Fukuda, J. Hasegawa, M. Ishida, T. Nakajima, Y. Honda, O. Kitao, H. Nakai, T. Vreven, J. A. Montgomery, Jr., J. E. Peralta, F. Ogliaro, M. Bearpark, J. J. Heyd, E. Brothers, K. N. Kudin, V. N. Staroverov, T. Keith, R. Kobayashi, J. Normand, K. Raghavachari, A. Rendell, J. C. Burant, S. S. Iyengar, J. Tomasi, M. Cossi, N. Rega, J. M. Millam, M. Klene, J. E. Knox, J. B. Cross, V. Bakken, C. Adamo, J. Jaramillo, R. Gomperts, R. E. Stratmann, O. Yazyev, A. J. Austin, R. Cammi, C. Pomelli, J. W. Ochterski, R. L. Martin, K. Morokuma, V. G. Zakrzewski, G. A. Voth, P. Salvador, J. J. Dannenberg, S. Dapprich, A. D. Daniels, O. Farkas, J. B. Foresman, J. V. Ortiz, J. Cioslowski, and D. J. Fox, *Gaussian 09, Revision D.01*, 2013, Gaussian, Inc.: Wallingford CT.

(5) Furche, F.; Ahlrichs, R., Adiabatic time-dependent density functional methods for excited state properties. *J. Chem. Phys.* **2002,** *117* (16), 7433-7447.

(6) Marenich, A. V.; Cramer, C. J.; Truhlar, D. G., Universal Solvation Model Based on Solute Electron Density and on a Continuum Model of the Solvent Defined by the Bulk Dielectric Constant and Atomic Surface Tensions. *J. Phys. Chem. B* **2009,** *113* (18), 6378-6396.

(7) Frisch, M. J. T., G. W.; Schlegel, H. B.; Scuseria, G. E.; Robb, M. A.; Cheeseman, J. R.; Scalmani, G.; Barone, V.; Petersson, G. A.; Nakatsuji, H.; Li, X.; Caricato, M.; Marenich, A. V.; Bloino, J.; Janesko, B. G.; Gomperts, R.; Mennucci, B.; Hratchian, H. P.; Ortiz, J. V.; Izmaylov, A. F.; Sonnenberg, J. L.; Williams; Ding, F.; Lipparini, F.; Egidi, F.; Goings, J.; Peng, B.; Petrone, A.; Henderson, T.; Ranasinghe, D.; Zakrzewski, V. G.; Gao, J.; Rega, N.; Zheng, G.; Liang, W.; Hada, M.; Ehara, M.; Toyota, K.; Fukuda, R.; Hasegawa, J.; Ishida, M.; Nakajima, T.; Honda, Y.; Kitao, O.; Nakai, H.; Vreven, T.; Throssell, K.; Montgomery Jr., J. A.; Peralta, J. E.; Ogliaro, F.; Bearpark, M. J.; Heyd, J. J.; Brothers, E. N.; Kudin, K. N.; Staroverov, V. N.; Keith, T. A.; Kobayashi, R.; Normand, J.; Raghavachari, K.; Rendell, A. P.; Burant, J. C.; Iyengar, S. S.; Tomasi, J.; Cossi, M.; Millam, J. M.; Klene, M.; Adamo, C.; Cammi, R.; Ochterski, J. W.; Martin, R. L.; Morokuma, K.; Farkas, O.; Foresman, J. B.; Fox, D. J. *Gaussian 16 Revision B.01*, 2016, Gaussian, Inc.: Wallingford CT.

(8) Lu, T.; Chen, F., Multiwfn: A multifunctional wavefunction analyzer. *J. Comput. Chem.* **2011,** *33* (5), 580-592.

7. Calculated Molecular Cartesian Coordinates

7.1 Energy and cartesian coordinates obtained in the gas phase at the B3LYP/6-31G(d,p)//B3LYP/6-31G(d,p) level of theory using Gaussian 09 program (revision B.01).

Phenanthrene

E(RB3LYP) = –539.53865629 a.u.

Charge = 0; Multiplicity = 1

	x	y	z
C	-1.50037	-1.56689	0.000002
C	-2.88294	-1.52929	0.000002
C	-0.72893	-0.38088	0.000000
C	0.728932	-0.38088	0.000000
C	1.422942	0.865919	0.000001
C	-1.42294	0.865919	-0.000001
C	2.882936	-1.52929	-0.000002
C	1.500372	-1.56689	-0.000002
C	3.561768	-0.29629	0.000000
C	2.837348	0.879097	0.000001
C	-2.83735	0.879097	-0.000001
C	-3.56177	-0.29629	0.000000
C	-0.67976	2.093902	-0.000001
C	0.679759	2.093902	0.000001
H	-1.00693	-2.53292	0.000004
H	-3.44684	-2.45823	0.000003
H	3.446844	-2.45823	-0.000003
H	1.00693	-2.53292	-0.000004
H	4.648038	-0.27186	0.000001
H	3.347948	1.839421	0.000002
H	-3.34795	1.839421	-0.000002
H	-4.64804	-0.27186	0.000000
H	-1.23282	3.030183	-0.000001
H	1.232823	3.030183	0.000002

TS (Phenanthrene + Acetylene)

E(RB3LYP) = –616.79437930 a.u.

Charge = 0; Multiplicity = 1

	x	y	z
C	-2.84672	-1.16443	-0.46799
C	-0.70337	0.04742	-0.34842
C	0.703368	0.047403	-0.34841
C	1.427624	1.235534	0.009779
C	-1.42762	1.235537	0.009807
C	2.846725	-1.16447	-0.46788
C	3.518433	-0.00849	-0.13012
C	2.817877	1.189703	0.107998
C	-2.81786	1.189697	0.108072
C	-3.51842	-0.00849	-0.13013
C	-0.67952	2.437297	0.277303
C	0.679534	2.437295	0.27729
H	-3.40142	-2.07335	-0.68791
H	3.401416	-2.07341	-0.68770
H	4.604193	-0.00998	-0.07322
H	3.362911	2.094183	0.366793
H	-3.36289	2.094162	0.366925
H	-4.60418	-0.00999	-0.07316
H	-1.23116	3.345065	0.510675
H	1.231182	3.345063	0.510648
C	-0.62945	-2.32679	1.000895
C	0.629381	-2.32681	1.000872
C	1.416805	-1.19712	-0.51898
H	0.986952	-1.93229	-1.19789
C	-1.41680	-1.19711	-0.51904
H	-0.98695	-1.93225	-1.19793
H	1.504065	-2.70220	1.498129
H	-1.50406	-2.70206	1.498378

TS (Phenanthrene + *N*-Phenylmaleimide)

E(RB3LYP) = –1129.95219286 a.u.

Charge = 0; Multiplicity = 1

	x	y	z
C	-1.16209	-2.87039	-0.38704
C	-2.37466	-0.70814	-0.41521
C	-2.36602	0.689992	-0.48187
C	-3.56599	1.439121	-0.21555
C	-3.58336	-1.41348	-0.07850
C	-1.12644	2.829991	-0.65719
C	-2.29317	3.513908	-0.41289
C	-3.50969	2.829208	-0.19044
C	-3.54422	-2.79539	0.079513
C	-2.33642	-3.51306	-0.07671
C	-4.79074	-0.64814	0.101059
C	-4.78252	0.709301	0.035604
H	-0.24165	-3.42887	-0.51690
H	-0.19907	3.361622	-0.83968
H	-2.29095	4.600778	-0.41074
H	-4.41926	3.394082	-0.00190
H	-4.46038	-3.32845	0.321161
H	-2.34733	-4.59465	0.029896
H	-5.71128	-1.18422	0.319011
H	-5.69638	1.274944	0.200587
C	-0.11890	-0.67361	1.048665
C	-0.11599	0.762881	0.987868
C	1.282718	-1.13098	0.788574
C	1.288506	1.19290	0.701224
O	1.711245	-2.26723	0.809129
O	1.718506	2.328189	0.644145
C	3.419886	-0.00708	0.081258
C	3.862500	-0.98369	-0.81944
C	4.320418	0.934457	0.593967
C	5.202808	-1.01671	-1.19972
H	3.165896	-1.7179	-1.20530
C	5.655940	0.897823	0.194521
H	3.973679	1.693108	1.283318
C	6.104033	-0.07583	-0.69883
H	5.540105	-1.78091	-1.89458
H	6.348722	1.634809	0.591453
H	7.147065	-0.10217	-1.00187
N	2.047372	0.02000	0.474657
H	-0.65873	1.358751	1.712275
H	-0.65598	-1.2026	1.827214
C	-1.09988	1.390229	-0.62692
H	-0.37284	0.931901	-1.29818
C	-1.11708	-1.43438	-0.49424
H	-0.38909	-1.05161	-1.21083

Perylene

E(RB3LYP) = –769.40611887 a.u.

Charge = 0; Multiplicity = 1

	x	y	z
C	-1.4794	2.427422	-0.00017
C	-0.73826	1.249957	-0.00012
C	0.738262	1.249957	0.000038
C	1.479397	2.427422	0.00002
C	2.886127	2.422689	0.00021
C	1.439617	-7E-06	0.000215
C	-1.43962	-7E-06	-0.00022
C	-2.88613	2.422689	-0.00036
C	0.738269	-1.24997	0.000182
C	1.479424	-2.42743	0.000417
C	2.88615	-2.42268	0.000648
C	3.575613	-1.23263	0.000643
C	2.874357	0.000002	0.000429
C	3.575601	1.232642	0.000423
C	-3.5756	1.232642	-0.0005
C	-2.87436	0.000002	-0.00043
C	-3.57561	-1.23263	-0.00057
C	-2.88615	-2.42268	-0.0005
C	-1.47942	-2.42743	-0.00027
C	-0.73827	-1.24997	-0.0001
H	0.977314	-3.3882	0.000434
H	3.420779	-3.36863	0.000825
H	-3.42078	-3.36863	-0.00061
H	-0.97731	-3.3882	-0.00022
H	4.662528	1.217279	0.000581
H	4.662541	-1.21726	0.000807
H	-4.66253	1.217279	-0.00066
H	-4.66254	-1.21726	-0.00073
H	-3.42074	3.368651	-0.0004
H	-0.97727	3.388181	-0.00006
H	0.977267	3.388181	-0.00015
H	3.420738	3.368651	0.00019

TS (Perylene + Acetylene)

E(RB3LYP) = –846.68409871 a.u.

Charge = 0; Multiplicity = 1

	x	y	z
C	-1.48995	2.761503	0.17021
C	-0.7336	1.595126	-0.01517
C	0.733664	1.595104	-0.01517
C	1.490041	2.761455	0.170246
C	2.884177	2.729732	0.22106
C	1.427881	0.357614	-0.17525
C	-1.42785	0.357654	-0.17525
C	-2.88408	2.729823	0.221021
C	0.710314	-0.86112	-0.41754
C	1.416743	-2.09609	-0.48566
C	2.85508	-2.08529	-0.43744
C	3.539582	-0.92792	-0.23514
C	2.856279	0.329345	-0.10111
C	3.564074	1.525941	0.09597
C	-3.56401	1.526048	0.095951
C	-2.85625	0.329432	-0.10111
C	-3.53959	-0.92781	-0.23513
C	-2.85512	-2.08521	-0.43743
C	-1.41678	-2.09607	-0.48566
C	-0.71033	-0.8611	-0.41754
H	3.388778	-3.02397	-0.56514
H	-3.38886	-3.02386	-0.56512
H	4.649733	1.497441	0.148961
H	4.62647	-0.92768	-0.19405
H	-4.64967	1.497579	0.148941
H	-4.62648	-0.92755	-0.19404
H	-3.43543	3.654544	0.368191
H	-0.99369	3.718341	0.288566
H	0.993815	3.718303	0.288637
H	3.435555	3.654434	0.368253
C	0.624569	-3.10049	1.255822
C	-0.62483	-3.10044	1.255747
H	1.550094	-3.36904	1.726867
H	-1.55029	-3.36891	1.727002
H	-0.96768	-2.92156	-1.02784
H	0.967584	-2.92159	-1.02775

TS (Perylene + *N*-Phenylmaleimide)

E(RB3LYP) = –1359.84566620 a.u.

Charge = 0; Multiplicity = 1

	x	y	z
C	4.980309	1.518621	0.099851
C	3.821482	0.747778	-0.08785
C	3.829968	-0.71617	-0.04311
C	4.997223	-1.46076	0.192353
C	4.969917	-2.85124	0.277742
C	2.602208	-1.423	-0.21505
C	2.585464	1.428618	-0.30043
C	4.936833	2.911278	0.101138
C	1.385541	-0.71939	-0.50484
C	0.149011	-1.44059	-0.58578
C	0.167244	-2.88126	-0.4967
C	1.326068	-3.54459	-0.25191
C	2.578714	-2.849	-0.1081
C	3.771883	-3.54274	0.13706
C	3.730578	3.579069	-0.07867
C	2.545317	2.858107	-0.27955
C	1.284549	3.529242	-0.46347
C	0.133049	2.839123	-0.66465
C	0.131387	1.395382	-0.66556
C	1.376985	0.694846	-0.54612
H	-0.77033	-3.41602	-0.6028
H	-0.81059	3.355338	-0.80323
H	3.749183	-4.62665	0.216304
H	1.332953	-4.62922	-0.17451
H	3.695327	4.665446	-0.06472
H	1.279241	4.616587	-0.45301
H	-0.63486	-1.02071	-1.20942
H	-0.64959	0.930647	-1.26028
C	-0.8635	0.731576	1.087805
C	-0.8638	-0.68693	1.123542
C	-2.25385	1.177975	0.783692
C	-2.25189	-1.14746	0.830995
O	-2.67836	2.316761	0.73451
O	-2.67905	-2.28552	0.820933
H	-0.2684	1.348959	1.747925
H	-0.27373	-1.27047	1.81795
N	-3.01425	0.010197	0.528714
H	5.892561	-3.39431	0.462671
H	5.948014	-0.95596	0.320952
H	5.937115	1.033455	0.256237
H	5.85328	3.475216	0.251163

C	-4.38043	-0.00721	0.116517
C	-4.81653	-0.97423	-0.798
C	-5.28358	0.935393	0.623352
C	-6.15152	-0.99663	-1.19736
H	-4.1191	-1.71007	-1.17897
C	-6.61335	0.909815	0.204374
H	-4.94248	1.686659	1.323451
C	-7.05457	-0.0543	-0.70262
H	-6.48313	-1.75384	-1.90261
H	-7.30737	1.647932	0.597144
H	-8.09332	-0.07223	-1.0207

Bisanthene

E(RB3LYP) = –1075.50262156 a.u.

Charge = 0 ; Multiplicity = 1

	x	y	z
C	-2.487666	0.736175	0.000097
C	-2.487666	-0.736175	-0.000095
C	-1.229900	-1.431763	-0.000047
C	0.000000	-0.726046	0.000004
C	0.000000	0.726046	-0.000004
C	-1.229900	1.431763	0.000047
C	1.229900	-1.431763	0.000057
C	1.222298	-2.872720	0.000111
C	2.458624	-3.579567	0.000263
C	3.647183	-2.894427	0.000382
C	3.659231	-1.483393	0.000304
C	2.487666	-0.736175	0.000089
C	-1.222298	2.872720	0.000096
C	-2.458624	3.579567	0.000262
C	-3.647184	2.894427	0.000414
C	-3.659231	1.483393	0.000346
C	-3.659231	-1.483393	-0.000342
C	-3.647183	-2.894427	-0.000411
C	-2.458623	-3.579567	-0.000261
C	-1.222298	-2.872720	-0.000096
C	0.000000	-3.552975	0.000010
C	3.659231	1.483393	-0.000304
C	2.487666	0.736175	-0.000090
C	3.647183	2.894427	-0.000384
C	2.458624	3.579567	-0.000266
C	1.222298	2.872720	-0.000113
C	1.229900	1.431763	-0.000058
C	0.000000	3.552975	-0.000013
H	2.438340	-4.666224	0.000303
H	4.591631	-3.431523	0.000539
H	4.622619	-0.986108	0.000436
H	-2.438340	4.666224	0.000294
H	-4.591631	3.431523	0.000592
H	-4.622619	0.986108	0.000519
H	-4.622619	-0.986108	-0.000513
H	-4.591631	-3.431524	-0.000588
H	-2.438340	-4.666224	-0.000293
H	0.000000	-4.640595	0.000010
H	4.622619	0.986108	-0.000434
H	4.591631	3.431524	-0.000540
H	2.438340	4.666224	-0.000308
H	0.000000	4.640595	-0.000014

TS (Bisanthene + Acetylene)

E(RB3LYP) = –1152.78972664 a.u.

Charge = 0; Multiplicity = 1

	x	y	z
C	1.550262	-2.866547	0.001424
C	1.570652	-1.431412	-0.030191
C	0.341931	-0.720058	-0.147731
C	-0.883390	-1.421239	-0.243583
C	-0.889953	-2.860972	-0.178842
C	0.311356	-3.542974	-0.063327
C	-2.114992	-0.713331	-0.409822
C	-2.114989	0.713341	-0.409825
C	-3.342002	1.429541	-0.417243
C	-3.342011	-1.429524	-0.417221
C	1.570656	1.431408	-0.030188
C	0.341933	0.720058	-0.147730
C	1.550272	2.866543	0.001431
C	0.311369	3.542975	-0.063322
C	-0.889943	2.860977	-0.178842
C	-0.883385	1.421244	-0.243585
C	-2.152291	3.550423	-0.238877
C	-3.320995	-2.867751	-0.366919
C	-3.320985	2.867766	-0.366935

C	-2.152304	-3.550413	-0.238873
H	0.306627	-4.630038	-0.019396
H	0.306643	4.630039	-0.019390
H	-2.145689	4.637257	-0.201154
H	-4.263674	-3.404867	-0.438300
H	-4.263661	3.404885	-0.438322
H	-2.145705	-4.637247	-0.201154
C	-4.256915	0.622738	1.433008
C	-4.256895	-0.622748	1.432996
C	2.819864	-0.735882	0.060871
C	2.819866	0.735873	0.060874
H	-4.210898	-0.979195	-0.882943
H	-4.210897	0.979207	-0.882944
H	-4.476552	-1.567314	1.889738
H	-4.476562	1.567312	1.889730
C	2.772323	3.575258	0.105751
H	2.749915	4.661902	0.127624
C	3.990234	1.488264	0.158102
H	4.952577	0.993182	0.225257
C	3.990229	-1.488277	0.158095
H	4.952574	-0.993198	0.225250
C	2.772310	-3.575267	0.105741
H	2.749899	-4.661910	0.127612
C	3.967759	2.892801	0.177955
H	4.904565	3.437933	0.255793
C	3.967749	-2.892814	0.177944
H	4.904554	-3.437949	0.255779

TS (Bisanthene + *N*-Phenylmaleimide)

E(RB3LYP) = –1665.95314318 a.u.

Charge = 0; Multiplicity = 1

	x	y	z
C	3.717792	-2.855222	0.044888
C	3.736057	-1.422031	-0.013599
C	2.510944	-0.718301	-0.206134
C	1.294517	-1.427569	-0.348934
C	1.288976	-2.866347	-0.259779
C	2.483871	-3.538907	-0.070806
C	0.072628	-0.728484	-0.590973
C	0.067285	0.694321	-0.621385
C	-1.167371	1.409491	-0.709881
C	-1.156297	-1.455903	-0.651165
C	3.725762	1.438831	-0.076915
C	2.505733	0.718534	-0.237860
C	3.697144	2.873023	-0.081851
C	2.458241	3.542029	-0.226661
C	1.268144	2.853271	-0.384997
C	1.284183	1.412050	-0.411001
C	0.005604	3.535207	-0.520147
C	-1.134159	-2.895088	-0.564925
C	-1.155505	2.851331	-0.688403
C	0.031450	-3.562630	-0.365035
H	2.481304	-4.624774	-0.006630
H	2.447862	4.629613	-0.210149
H	0.007008	4.622306	-0.499026
H	-2.075345	-3.428755	-0.641634
H	-2.100445	3.374031	-0.789509
H	0.040315	-4.647625	-0.294749
C	-2.138323	0.717589	1.128123
C	-2.141890	-0.693672	1.152615
C	4.975262	-0.719016	0.124270
C	4.969959	0.751545	0.091442
H	-1.975389	-1.026540	-1.218082
H	-1.985416	0.950325	-1.254416
H	-1.507215	1.341669	1.746073
C	4.907685	3.590535	0.066187
H	4.880218	4.677086	0.060799
C	6.131833	1.512634	0.228980
H	7.091644	1.024381	0.354967
C	6.142443	-1.464919	0.295874
H	7.098669	-0.964751	0.400710
C	4.933373	-3.556775	0.224777
H	4.913703	-4.642684	0.267186
C	6.101961	2.915842	0.214438
H	7.030945	3.468183	0.325625
C	6.122647	-2.867584	0.343501
H	7.055512	-3.407745	0.479283
H	-1.518368	-1.298893	1.796386
C	-3.524897	1.174191	0.836164
C	-3.528957	-1.152723	0.866731
O	-3.944212	2.315816	0.792197
C	-5.663322	-0.000422	0.186713
C	-6.114785	-0.964112	-0.724122
C	-6.556195	0.946917	0.703252
C	-7.453956	-0.978475	-1.109653
H	-5.425845	-1.703989	-1.112450
C	-7.890279	0.929406	0.297867

H	-6.203671	1.695680	1.400340	H	-9.388613	-0.043083	-0.912623
C	-8.346564	-0.031393	-0.605208	N	-4.293337	0.009671	0.584608
H	-7.797060	-1.733313	-1.811992	O	-3.956609	-2.291207	0.846672
H	-8.575868	1.671279	0.698350				

Peritetracene

E(RB3LYP) = –1381.59929998 a.u.

Charge = 0; Multiplicity = 1

	x	y	z				
C	1.236077	0.721740	-0.000004	C	4.875868	-2.896688	-0.000249
C	1.236077	-0.721740	0.000001	C	3.686347	-3.581626	-0.000159
C	0.000000	-1.423143	0.000014	C	2.449531	-2.874878	-0.000064
C	-1.236077	-0.721740	0.000012	C	1.227725	-3.552143	-0.000018
C	-1.236077	0.721740	-0.000009	C	-1.227725	-3.552143	0.000067
C	0.000000	1.423143	-0.000014	C	-2.449531	-2.874878	0.000083
C	0.000000	2.868407	-0.000024	C	-3.686347	-3.581626	0.000146
C	-2.463200	1.431272	-0.000035	C	-4.875868	-2.896688	0.000183
C	-2.463200	-1.431272	0.000040	C	-4.891629	-1.486356	0.000137
C	0.000000	-2.868407	0.000024	C	-3.719685	-0.736230	0.000045
C	2.463200	-1.431272	-0.000029	H	-5.855660	0.990499	-0.000172
C	2.463200	1.431272	0.000021	H	-5.819136	3.435716	-0.000236
C	-3.719685	0.736230	-0.000038	H	5.819136	3.435716	0.000355
C	-4.891629	1.486356	-0.000128	H	5.855660	0.990498	0.000323
C	-4.875868	2.896688	-0.000172	H	5.855660	-0.990498	-0.000332
C	-3.686347	3.581626	-0.000137	H	5.819136	-3.435716	-0.000354
C	-2.449531	2.874878	-0.000077	H	-5.819136	-3.435716	0.000251
C	-1.227725	3.552143	-0.000061	H	-5.855660	-0.990499	0.000180
C	1.227725	3.552143	0.000013	H	3.666385	4.668181	0.000156
C	2.449531	2.874878	0.000053	H	1.224498	4.639573	0.000024
C	3.686348	3.581626	0.000145	H	-1.224498	4.639573	-0.000080
C	4.875868	2.896688	0.000242	H	-3.666385	4.668181	-0.000163
C	4.891629	1.486356	0.000209	H	3.666385	-4.668181	-0.000182
C	3.719685	0.736230	0.000054	H	1.224498	-4.639573	-0.000028
C	3.719685	-0.736230	-0.000060	H	-1.224498	-4.639573	0.000088
C	4.891629	-1.486356	-0.000214	H	-3.666385	-4.668181	0.000171

TS (Peritetracene + Acetylene)

E(RB3LYP) = –1458.89007569 a.u.

Charge = 0; Multiplicity = 1

	x	y	z				
C	1.569892	-0.721364	-0.043758	C	0.331893	-2.865594	-0.077426
C	1.569893	0.721364	-0.043758	C	-2.120217	-1.422004	-0.253681
C	0.340215	1.423434	-0.105228	C	-2.120216	1.422007	-0.253681
C	-0.893604	0.717156	-0.189216	C	0.331895	2.865595	-0.077425
C	-0.893604	-0.717155	-0.189216	C	2.797785	1.431973	0.024947
C	0.340214	-1.423434	-0.105229	C	2.797783	-1.431975	0.024945
				C	-3.351951	-0.716179	-0.382873

C	-4.577218	-1.435400	-0.362416
C	-4.552814	-2.872046	-0.313045
C	-3.380362	-3.555487	-0.217665
C	-2.116992	-2.866016	-0.191737
C	-0.913709	-3.545361	-0.111024
C	1.548705	-3.549022	-0.009128
C	2.778614	-2.871821	0.037130
C	4.009215	-3.579483	0.100275
C	5.201689	-2.895811	0.149734
C	5.222048	-1.488070	0.142181
C	4.050779	-0.736275	0.083239
C	4.050780	0.736273	0.083239
C	5.222049	1.488067	0.142182
C	5.201692	2.895808	0.149737
C	4.009218	3.579481	0.100278
C	2.778616	2.871820	0.037132
C	1.548708	3.549022	-0.009125
C	-0.913706	3.545363	-0.111022
C	-2.116990	2.866018	-0.191737
C	-3.380358	3.555491	-0.217665
C	-4.552811	2.872050	-0.313049
C	-4.577215	1.435406	-0.362423
C	-3.351950	0.716183	-0.382874
H	-5.496463	-3.410169	-0.359655
H	6.141773	-3.438793	0.197215
H	6.186019	-0.993558	0.185741
H	6.186020	0.993553	0.185742
H	6.141775	3.438789	0.197218
H	-5.496460	3.410174	-0.359662
H	3.989034	-4.666267	0.108596
H	1.545971	-4.636598	0.009476
H	-0.914662	-4.632404	-0.069113
H	-3.373340	-4.642316	-0.181486
H	3.989038	4.666264	0.108600
H	1.545974	4.636598	0.009479
H	-0.914658	4.632406	-0.069111
H	-3.373336	4.642319	-0.181485
H	-5.463288	-0.982879	-0.791256
H	-5.463287	0.982882	-0.791254
C	-5.440420	0.621951	1.538270
C	-5.440412	-0.621963	1.538264
H	-5.636465	1.574146	1.989023
H	-5.636454	-1.574158	1.989018

TS (Peritetracene + *N*-Phenylmaleimide)

E(RB3LYP) = –1972.05431718 a.u.

Charge = 0; Multiplicity = 1

	x	y	z
C	3.682211	-0.716726	-0.046071
C	3.678806	0.724860	-0.071338
C	2.453351	1.421587	-0.207195
C	1.227006	0.709474	-0.340769
C	1.230375	-0.722239	-0.315555
C	2.460025	-1.423527	-0.157341
C	2.452792	-2.864461	-0.107532
C	0.009778	-1.432434	-0.432783
C	0.003085	1.409415	-0.482441
C	2.439283	2.863318	-0.207726
C	4.900616	1.440947	0.045976
C	4.907387	-1.422530	0.095854
C	-1.211717	-0.732724	-0.640095
C	-2.440243	-1.460946	-0.680886
C	-2.417491	-2.898286	-0.598052
C	-1.248166	-3.569796	-0.427544
C	0.012375	-2.876142	-0.350873
C	1.209977	-3.548849	-0.196577
C	3.664088	-3.543101	0.033850
C	4.890298	-2.861200	0.131088
C	6.116411	-3.564561	0.267211
C	7.304099	-2.876478	0.364227
C	7.322040	-1.469586	0.333852
C	6.153474	-0.721827	0.204315
C	6.149946	0.750352	0.178807
C	7.314913	1.507668	0.282693
C	7.290315	2.914662	0.264267
C	6.099422	3.593322	0.143119
C	4.876710	2.879870	0.031126
C	3.647313	3.552173	-0.089893
C	1.193248	3.538327	-0.320430
C	-0.001199	2.855089	-0.450975
C	-1.264922	3.539779	-0.552004
C	-2.431358	2.857451	-0.697433
C	-2.447467	1.417886	-0.728122
C	-1.215192	0.697274	-0.664618
H	-3.360923	-3.430634	-0.654524
H	8.241547	-3.415995	0.467218
H	8.281738	-0.971845	0.416441

H	8.276876	1.017579	0.382782
H	8.225144	3.461871	0.348864
H	-3.377214	3.382767	-0.774048
H	6.098241	-4.650984	0.292654
H	3.662908	-4.630118	0.069778
H	1.209522	-4.634994	-0.138365
H	-1.240540	-4.654999	-0.360982
H	6.076119	4.679876	0.131045
H	3.640997	4.639764	-0.091538
H	1.187707	4.625821	-0.300000
H	-1.262031	4.626711	-0.525005
H	-3.278392	-1.025475	-1.213182
H	-3.285529	0.960497	-1.241779
C	-3.387894	0.712278	1.157289
C	-3.393247	-0.696258	1.176099
C	-4.775388	1.173911	0.885471
C	-4.782706	-1.153910	0.907428
O	-5.192480	2.317017	0.848474
O	-5.211389	-2.292434	0.886550
H	-2.734746	1.338191	1.749491
H	-2.749027	-1.310885	1.789409
N	-5.550322	0.011089	0.643125
C	-6.925893	0.004849	0.265904
C	-7.394174	-0.957597	-0.637906
C	-7.808207	0.955186	0.795189
C	-8.738976	-0.967747	-1.003449
H	-6.713620	-1.699952	-1.036069
C	-9.148195	0.941837	0.409647
H	-7.443020	1.703045	1.486677
C	-9.621043	-0.017714	-0.486217
H	-9.094820	-1.721712	-1.700394
H	-9.825329	1.686057	0.820072
H	-10.667579	-0.026145	-0.778132

DBOV

E(RB3LYP) = –1457.87282427 a.u.

Charge = 0; Multiplicity = 1

	x	y	z
C	0.681937	2.477176	-0.000031
C	-0.576444	1.802397	-0.000005
C	-1.785174	2.555597	0.000024
C	-1.696855	3.959349	0.000064
C	-0.477574	4.615736	0.000028
C	0.729091	3.901930	-0.000032
C	-3.073584	1.856519	0.000013
C	-3.084084	0.425184	0.000036
C	-4.335463	-0.277365	0.000047
C	-5.545255	0.459507	0.000037
C	-5.519464	1.838015	-0.000009
C	-4.297254	2.528981	-0.000028
C	-0.601594	0.367840	0.000007
C	-1.867046	-0.316023	0.000027
C	-1.898255	-1.730402	0.000017
C	-3.161099	-2.422654	0.000028
C	-4.337321	-1.689825	0.000051
C	0.601595	-0.367837	-0.000003
C	0.576450	-1.802399	-0.000006
C	-0.681937	-2.477178	-0.000017
C	1.898253	1.730402	-0.000050
C	1.867039	0.316026	-0.000026
C	-0.729095	-3.901924	-0.000072
C	1.785174	-2.555588	-0.000030
C	0.477582	-4.615736	-0.000163
C	1.696853	-3.959357	-0.000154
C	3.084087	-0.425190	0.000001
C	3.073579	-1.856517	0.000060
C	-3.164514	-3.862067	0.000000
C	-2.003688	-4.566541	-0.000054
C	2.003693	4.566542	-0.000079
C	3.164514	3.862071	-0.000104
C	3.161105	2.422653	-0.000076
C	4.337317	1.689830	-0.000053
C	4.335465	0.277361	0.000007
C	4.297259	-2.528985	0.000213
C	5.545248	-0.459505	0.000098
C	5.519457	-1.838024	0.000222
H	-2.600069	4.558508	0.000137
H	-0.450142	5.702631	0.000055
H	-6.489027	-0.079606	0.000054
H	-6.448029	2.402211	-0.000035
H	-4.325142	3.612478	-0.000091
H	-5.291910	-2.211403	0.000060
H	0.450134	-5.702631	-0.000254
H	2.600073	-4.558505	-0.000274
H	-4.123980	-4.373127	0.000017
H	-2.015847	-5.653693	-0.000086
H	2.015847	5.653694	-0.000092

H	4.123982	4.373127	-0.000137
H	5.291910	2.211401	-0.000065
H	4.325140	-3.612481	0.000357
H	6.489028	0.079594	0.000092
H	6.448028	-2.402210	0.000333

TS (DBOV + Acetylene)

E(RB3LYP) = –1535.14646376 a.u.

Charge = 0; Multiplicity = 1

	x	y	z
C	2.062343	3.390752	-0.036031
C	1.056161	2.373668	-0.075004
C	1.435797	1.009650	-0.168423
C	2.808574	0.655217	-0.227598
C	3.806629	1.679738	-0.153258
C	3.412905	3.017764	-0.066329
C	3.192724	-0.713913	-0.367994
C	2.200488	-1.727278	-0.384066
C	2.579146	-3.106119	-0.376766
C	4.577973	-1.074571	-0.311727
C	-0.939246	0.336723	-0.085096
C	0.429041	-0.010645	-0.176080
C	-1.932899	-0.687963	-0.054641
C	-1.539410	-2.052572	-0.096870
C	-0.168189	-2.398828	-0.187679
C	0.820777	-1.381262	-0.249527
C	0.230333	-3.785073	-0.225129
C	5.559252	-0.012943	-0.273075
C	1.571221	-4.105505	-0.338496
C	5.191453	1.292532	-0.177881
H	4.175634	3.792397	-0.019204
H	6.612360	-0.280691	-0.316072
H	1.862574	-5.151378	-0.406938
H	5.944657	2.076402	-0.142339
C	3.814157	-3.106190	1.477273
C	4.659871	-2.184429	1.421490
C	-0.325343	2.729708	-0.012475
C	-1.333157	1.715865	-0.015976
C	-3.315729	-0.341039	0.025918
C	-2.705710	2.076067	0.056674
C	-0.692477	4.102864	0.063549
C	1.652936	4.762403	0.042259
H	2.421806	5.530824	0.066787
C	-3.725619	1.027964	0.072609
C	0.333561	5.102651	0.084677
H	0.035464	6.146736	0.142427
C	-3.031902	3.449273	0.120140
C	-2.061108	4.430623	0.123447
H	-4.070372	3.755828	0.173700
H	-2.351264	5.477116	0.178652
H	4.889839	-1.970822	-0.841580
H	3.528826	-3.390397	-0.816988
C	-0.799463	-4.797079	-0.177190
H	-0.493144	-5.839966	-0.209836
C	-4.298446	-1.384259	0.059881
C	-5.097842	1.306460	0.140367
H	-5.445366	2.332715	0.173496
C	-5.665135	-1.043429	0.131500
H	-6.407105	-1.837508	0.156494
C	-2.111557	-4.467647	-0.096034
H	-2.874345	-5.241774	-0.061350
C	-6.052658	0.285212	0.168548
H	-7.106906	0.543133	0.222118
C	-2.542597	-3.089320	-0.053112
C	-3.875290	-2.740444	0.021186
H	-4.632791	-3.520873	0.050033
H	3.355278	-3.936400	1.975496
H	5.465472	-1.693086	1.936752

TS (DBOV + *N*-Phenylmaleimide)

E(RB3LYP) = –2048.30892385 a.u.

Charge = 0; Multiplicity = 1

	x	y	z
C	-2.018891	4.086634	-0.129091
C	-2.318065	2.689401	-0.139961
C	-1.268593	1.745481	-0.296966
C	0.074891	2.187311	-0.436322
C	0.361200	3.593572	-0.406121
C	-0.682001	4.502636	-0.259936
C	1.125761	1.244875	-0.625213
C	0.829896	-0.139603	-0.666093
C	1.870259	-1.109706	-0.798188

C	2.518138	1.686149	-0.615112
C	-2.900161	-0.100711	-0.114533
C	-1.567006	0.346308	-0.290621
C	-3.180747	-1.498593	-0.077332
C	-2.119302	-2.434981	-0.204543
C	-0.787790	-1.986378	-0.390364
C	-0.509329	-0.594645	-0.450981
C	0.287307	-2.937690	-0.511086
C	2.745909	3.136577	-0.675535
C	1.582885	-2.483124	-0.727166
C	1.732429	4.022118	-0.548689
H	-0.458955	5.567283	-0.245995
H	3.770610	3.477016	-0.772611
H	2.392320	-3.198361	-0.837533
H	1.932223	5.090965	-0.567530
C	3.037712	-0.346823	1.201244
C	3.262229	1.047108	0.952311
C	-3.665661	2.244133	0.012647
C	-3.967677	0.847245	0.029350
H	2.233625	-0.764069	1.788736
C	-4.522908	-1.952823	0.095495
C	-5.309918	0.409753	0.191747
C	-4.707881	3.201977	0.156848
C	-3.093161	5.020639	0.017915
H	-2.859792	6.082362	0.021027
C	-5.599751	-1.022272	0.224021
C	-4.383297	4.595452	0.151408
H	-5.190942	5.314925	0.261352
H	2.863657	1.781836	1.648491
C	4.267394	-1.093946	1.003932
C	4.762010	1.166254	0.704444
O	4.480035	-2.289431	1.116906
C	6.618293	-0.465228	0.284204
C	7.678622	0.346915	0.707754
C	6.878951	-1.615408	-0.472912
C	8.986928	0.009057	0.363436
H	7.476523	1.239355	1.285144
C	8.192636	-1.947361	-0.798463
H	6.059853	-2.250186	-0.786066
C	9.251977	-1.137092	-0.386863
H	9.802837	0.647635	0.691283
H	8.385169	-2.844146	-1.381269
H	10.274191	-1.397239	-0.647667
N	5.272946	-0.131695	0.616586
O	5.393473	2.197788	0.567906
C	-6.319900	1.390401	0.323623
C	-6.031349	2.739259	0.306812
H	-7.353735	1.088435	0.445755
H	-6.834132	3.464538	0.414520
H	3.164076	1.138745	-1.304066
H	2.817984	-0.815552	-1.229707
C	-0.024844	-4.343113	-0.438882
H	0.793108	-5.053433	-0.525737
C	-4.784178	-3.361299	0.140046
C	-6.897551	-1.529843	0.383563
H	-7.738906	-0.853588	0.481767
C	-6.109853	-3.813370	0.305030
H	-6.303566	-4.882371	0.338253
C	-1.299620	-4.775346	-0.267824
H	-1.519448	-5.838758	-0.215730
C	-7.148246	-2.904626	0.422684
H	-8.168199	-3.257008	0.548353
C	-2.399058	-3.848417	-0.147001
C	-3.702247	-4.274091	0.019617
H	-3.915834	-5.339976	0.060658

“Tetrazigzag” HBC

E(RB3LYP) = –1916.47794353 a.u.

Charge = 0; Multiplicity = 1

	x	y	z
C	-0.710944	1.232767	0.000000
C	0.710944	1.232767	0.000000
C	1.426018	2.477442	0.000006
C	0.726804	3.715183	0.000013
C	-0.726804	3.715183	-0.000010
C	-1.426017	2.477442	-0.000006
C	2.855071	2.466444	0.000010
C	3.564657	1.226950	0.000001
C	4.996988	1.222083	-0.000002
C	5.692562	2.476202	0.000006
C	5.012785	3.656938	0.000021
C	3.580782	3.688992	0.000025
C	1.418404	0.000000	-0.000002
C	2.854651	0.000000	-0.000004
C	3.564657	-1.226951	-0.000008
C	4.996988	-1.222084	-0.000012
C	5.677884	0.000000	-0.000011

C	0.710944	-1.232767	0.000000
C	1.426017	-2.477442	0.000000
C	2.855071	-2.466444	-0.000004
C	-1.418404	0.000000	0.000002
C	-0.710944	-1.232767	0.000002
C	3.580782	-3.688992	-0.000005
C	0.726804	-3.715183	0.000006
C	2.860837	-4.901542	0.000005
C	1.483334	-4.912293	0.000012
C	-1.426018	-2.477442	0.000005
C	-0.726804	-3.715183	0.000005
C	5.692561	-2.476203	-0.000016
C	5.012785	-3.656938	-0.000012
C	-2.855071	2.466444	-0.000012
C	-3.564657	1.226951	-0.000005
C	-2.854651	0.000000	0.000001
C	-3.564657	-1.226950	0.000005
C	-2.855071	-2.466444	0.000006
C	-1.483335	-4.912293	0.000003
C	-3.580782	-3.688992	0.000008
C	-2.860837	-4.901542	0.000004
C	-4.996988	-1.222083	0.000007
C	-5.012785	-3.656938	0.000011
C	-5.692562	-2.476202	0.000012
C	-4.996988	1.222084	-0.000006
C	-3.580782	3.688992	-0.000027
C	-5.692561	2.476203	-0.000016
C	-5.012785	3.656938	-0.000028
C	-1.483334	4.912293	-0.000039
C	-2.860837	4.901542	-0.000046
C	2.860837	4.901542	0.000048
C	1.483335	4.912293	0.000044
C	-5.677884	0.000000	0.000002
H	6.779574	2.463704	0.000002
H	5.549754	4.602327	0.000031
H	6.765768	0.000000	-0.000015
H	3.409424	-5.840272	0.000008
H	6.779574	-2.463705	-0.000021
H	5.549754	-4.602328	-0.000013
H	-3.409424	-5.840272	0.000002
H	-5.549754	-4.602328	0.000013
H	-6.779574	-2.463704	0.000015
H	-6.779574	2.463705	-0.000016
H	-5.549754	4.602328	-0.000038
H	-0.978011	5.870799	-0.000067
H	-3.409424	5.840273	-0.000069
H	3.409424	5.840272	0.000072
H	0.978011	5.870798	0.000075
H	-6.765768	0.000000	0.000003
H	0.978011	-5.870798	0.000025
H	-0.978012	-5.870798	-0.000006

TS (“Tetrazigzag” HBC + Acetylene)

E(RB3LYP) = –1993.73735554 a.u.

Charge = 0; Multiplicity = 1

	x	y	z
C	0.709221	-1.490598	-0.057816
C	-0.709136	-1.490629	-0.057774
C	-1.425002	-2.730506	0.000873
C	-0.724875	-3.967838	0.043626
C	0.725078	-3.967803	0.043664
C	1.425146	-2.730438	0.000885
C	-2.855312	-2.713308	0.020728
C	-3.562551	-1.472934	-0.008637
C	-4.989984	-1.459693	0.028538
C	-5.687760	-2.705469	0.084760
C	-5.011199	-3.893212	0.106443
C	-3.584171	-3.932661	0.077880
C	-1.419082	-0.255961	-0.107964
C	-2.847457	-0.246121	-0.072468
C	-3.550999	0.987102	-0.092567
C	-4.985629	0.987266	-0.046559
C	-5.667058	-0.223797	0.009743
C	-0.710710	0.971413	-0.185974
C	-1.422592	2.211306	-0.233460
C	-2.837640	2.215138	-0.162335
C	1.419107	-0.255895	-0.107962
C	0.710676	0.971443	-0.186014
C	-3.550798	3.467665	-0.173542
C	-0.705282	3.438282	-0.349462
C	-2.845114	4.655613	-0.262996
C	-1.419157	4.690375	-0.287393
C	1.422498	2.211368	-0.233476
C	0.705120	3.438316	-0.349471
C	-5.672157	2.255902	-0.067074
C	-4.993457	3.430145	-0.126565
C	2.855456	-2.713172	0.020723

C	3.562636	-1.472765	-0.008669
C	2.847484	-0.245986	-0.072530
C	3.550967	0.987270	-0.092607
C	2.837547	2.215278	-0.162365
C	1.418981	4.690381	-0.287457
C	3.550647	3.467831	-0.173490
C	2.844889	4.655761	-0.262862
C	4.985595	0.987504	-0.046601
C	4.993301	3.430384	-0.126521
C	5.672062	2.256171	-0.067086
C	4.990069	-1.459455	0.028488
C	3.584372	-3.932488	0.077933
C	5.687906	-2.705199	0.084728
C	5.011400	-3.892971	0.106467
C	1.488272	-5.164790	0.093185
C	2.862995	-5.149261	0.109010
C	-2.862736	-5.149404	0.108841
C	-1.488012	-5.164866	0.093014
C	5.667085	-0.223530	0.009684
H	-6.774624	-2.690812	0.110427
H	-5.554571	-4.834062	0.149484
H	-6.754733	-0.220980	0.039857
H	-3.393672	5.593910	-0.311857
H	-6.758987	2.250001	-0.032271
H	-5.530557	4.375565	-0.140883
H	3.393390	5.594093	-0.311666
H	5.530356	4.375831	-0.140823
H	6.758893	2.250323	-0.032301
H	6.774770	-2.690490	0.110376
H	5.554816	-4.833794	0.149531
H	0.984875	-6.124140	0.122439
H	3.414453	-6.085531	0.149888
H	-3.414151	-6.085704	0.149619
H	-0.984571	-6.124198	0.122109
H	6.754760	-0.220660	0.039796
C	-0.629194	5.499143	1.445744
C	0.628597	5.499253	1.445939
H	-1.511980	5.761498	1.998341
H	1.511583	5.761362	1.998296
H	-0.981459	5.545138	-0.798954
H	0.981157	5.545296	-0.798635

TS (“Tetrazigzag” HBC + *N*-Phenylmaleimide)

E(RB3LYP) = –2506.89588416 a.u.

Charge = 0; Multiplicity = 1

	x	y	z
C	3.174412	-0.707195	-0.048072
C	3.176540	0.710537	-0.057118
C	4.411421	1.425707	0.065726
C	5.643661	0.724858	0.181307
C	5.641973	-0.724259	0.187892
C	4.407727	-1.423611	0.081606
C	4.393816	2.856106	0.075476
C	3.158375	3.563770	-0.030669
C	3.143587	4.990646	-0.004422
C	4.383517	5.688403	0.120265
C	5.568206	5.011338	0.215082
C	5.608508	3.584904	0.197586
C	1.947599	1.421499	-0.182547
C	1.936009	2.848668	-0.161749
C	0.706846	3.552906	-0.263908
C	0.704565	4.987871	-0.226654
C	1.910012	5.668273	-0.100994
C	0.725344	0.713125	-0.323573
C	-0.505075	1.425825	-0.457488
C	-0.514584	2.840309	-0.406407
C	1.942697	-1.416807	-0.160649
C	0.722294	-0.707833	-0.309699
C	-1.765545	3.554200	-0.503213
C	-1.725266	0.705079	-0.640244
C	-2.947653	2.853472	-0.671871
C	-0.514058	-1.418656	-0.416073
C	-1.730612	-0.699303	-0.603705
C	-0.560918	5.674502	-0.326385
C	-1.729998	4.996954	-0.456297
C	4.386978	-2.853706	0.106538
C	3.149417	-3.559608	0.013187
C	1.927842	-2.843202	-0.119269
C	0.695470	-3.545995	-0.204436
C	-0.524269	-2.833171	-0.342288
C	-1.780305	-3.547518	-0.423249
C	-2.960092	-2.854129	-0.571820
C	0.691925	-4.982056	-0.155483
C	-1.743832	-4.993161	-0.370544
C	-0.575661	-5.669428	-0.244153
C	3.131870	-4.985864	0.053550
C	5.600446	-3.583851	0.231545

C	4.370131	-5.684984	0.179026
C	5.557060	-5.009463	0.262703
C	6.834181	-1.489218	0.304391
C	6.815589	-2.863229	0.324680
C	6.821562	2.862618	0.301694
C	6.837109	1.488286	0.294043
C	1.895581	-5.662534	-0.031371
H	4.368423	6.775313	0.138257
H	6.504999	5.555108	0.309094
H	1.906475	6.755999	-0.076956
H	-3.884551	3.393361	-0.766841
H	-0.556798	6.761264	-0.292759
H	-2.672931	5.532816	-0.527772
H	-3.897991	-3.395872	-0.638194
H	-2.687874	-5.528260	-0.432638
H	-0.570834	-6.755958	-0.204365
H	4.352874	-6.771628	0.207153
H	6.492943	-5.554598	0.358002
H	7.791270	-0.986822	0.382188
H	7.747402	-3.415832	0.417164
H	7.754295	3.413936	0.392427
H	7.792849	0.984650	0.380161
H	1.890850	-6.750005	0.001123
C	-5.258774	1.133130	0.911893
C	-5.296989	-1.191359	0.801812
C	-3.867042	0.663545	1.076360
O	-5.734451	-2.324020	0.757118
C	-3.858341	-0.773006	0.996356
O	-5.681956	2.272452	0.971412
C	-2.973989	1.429818	-0.699148
C	-3.007875	-1.407821	-0.582452
H	-3.306057	-1.352790	1.729232
H	-3.222405	1.221846	1.742050
H	-3.764456	-1.005805	-1.259746
H	-3.786787	0.980561	-1.264665
C	-7.448587	0.006664	0.322825
C	-7.962239	-0.894224	-0.618690
C	-8.298953	0.924514	0.951452
C	-9.321952	-0.876496	-0.923175
H	-7.304714	-1.610529	-1.095971
C	-9.655106	0.940118	0.626790
H	-7.897428	1.625241	1.671446
C	-10.173435	0.041448	-0.306261
H	-9.713635	-1.582387	-1.650500
H	-10.308638	1.658700	1.113848
H	-11.232074	0.055331	-0.550363
N	-6.055808	-0.016501	0.636018

Acetylene

E(RB3LYP) = –77.32564618 a.u.

Charge = 0; Multiplicity = 1

	x	y	z
C	0.000000	0.000000	0.602488
H	0.000000	0.000000	1.669114
C	0.000000	0.000000	-0.602488
H	0.000000	0.000000	-1.669114

***N*-Phenylmaleimide**

E(RB3LYP) = –590.47883683 a.u.

Charge = 0; Multiplicity = 1

	x	y	z
C	-1.575995	-1.128766	0.239557
C	-2.994327	-0.652263	0.139470
C	-2.994084	0.652783	-0.139858
C	-1.575577	1.128840	-0.239550
N	-0.762257	-0.000112	-0.000085
H	-3.827206	-1.328069	0.285563
H	-3.826705	1.328857	-0.286170
O	-1.194969	2.254858	-0.475865
O	-1.195875	-2.254856	0.476337
C	0.665999	-0.000211	0.000004
C	1.362892	-1.068496	-0.575844
C	1.362703	1.068236	0.575728
C	2.756985	-1.065828	-0.564615
H	0.819146	-1.896212	-1.014141
C	2.756795	1.065744	0.564633
H	0.818782	1.895966	1.013832
C	3.458934	-0.000018	0.000082
H	3.293790	-1.900608	-1.006634
H	3.293445	1.900644	1.006612
H	4.545355	0.000063	0.000115

7.2 Cartesian coordinates of the optimized geometries of the species involved in the Diels-Alder cycloaddition of DBOV-Mes 1 with *N*-hexylmaleimide (2a). They were obtained using diphenyl ether as solvent with the polarizable continuum model (PCM) at the level of theory B3LYP/6-31G(d)//B3LYP/6-31G(d) with the Gaussian 09 program (revision B.01).

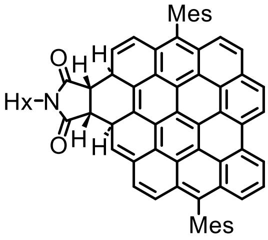

Int1-exo

Charge = 0; Multiplicity = 1

	x	y	z				
C	4.439355	-2.977286	0.422285	C	2.793163	3.298222	-0.347505
C	3.230935	-3.789853	0.444260	C	4.003930	2.711467	-0.235774
C	1.965523	-3.163872	0.318471	C	4.153233	1.276728	-0.056189
C	1.870692	-1.738112	0.174481	C	2.944601	0.473563	-0.008796
C	3.044923	-0.944158	0.145502	C	5.396884	0.685366	0.063582
C	4.329984	-1.560977	0.266509	C	-4.219112	-4.317563	0.042291
C	0.595220	-1.119345	0.059564	C	6.648622	1.513340	0.024767
C	-0.595693	-1.909103	0.071143	C	-3.206134	1.862986	-1.453410
C	-1.862834	-1.291276	-0.047599	C	-1.918097	2.721872	-1.469624
C	-1.935329	0.130701	-0.154986	C	-4.350330	2.876579	-1.407580
C	-0.788550	0.898432	-0.165813	C	-2.411014	4.168646	-1.434163
C	0.501348	0.293875	-0.072110	O	-5.545300	2.650735	-1.379436
C	5.510303	-0.748022	0.231956	O	-1.741242	5.184200	-1.431620
C	6.764817	-1.367456	0.360381	C	7.302205	1.752307	-1.202518
C	6.863634	-2.746604	0.517075	C	8.476682	2.512881	-1.212494
C	5.723877	-3.539797	0.546568	C	9.022416	3.046265	-0.042141
C	3.270374	-5.202141	0.585220	C	8.360929	2.795414	1.163507
C	2.125069	-5.960960	0.597414	C	7.184733	2.040861	1.219335
C	0.849473	-5.362330	0.469572	C	-4.708580	-4.834999	-1.176324
C	0.769148	-3.949591	0.330568	C	-5.871625	-5.611690	-1.168881
C	-0.351724	-6.126863	0.473582	C	-6.563934	-5.895590	0.011829
C	-1.575387	-5.528593	0.343790	C	-6.063619	-5.372409	1.206824
C	-1.698265	-4.111753	0.201837	C	-4.904203	-4.590027	1.245046
C	-0.510408	-3.326773	0.201464	C	-4.410478	-4.041729	2.565517
C	-2.974193	-3.477317	0.058286	C	-4.000553	-4.552461	-2.482749
C	-3.052797	-2.092030	-0.067274	C	-7.802140	-6.761923	-0.002250
C	-4.331794	-1.405687	-0.237679	C	6.505600	1.802589	2.549690
C	-4.443419	-0.072064	-0.335076	C	6.751389	1.201618	-2.499103
C	-3.262127	0.852569	-0.255268	C	10.276212	3.889151	-0.079243
C	-0.947032	2.399371	-0.278030	N	-3.802671	4.156622	-1.395346
C	0.351717	3.137329	-0.373832	C	-7.312174	9.244166	3.049996
C	1.562477	2.532214	-0.284407	C	-6.971185	8.804578	1.622576
C	1.682162	1.081934	-0.114637	C	-6.122786	7.527364	1.571069
				C	-5.781409	7.078614	0.144406

C	-4.931562	5.802580	0.099676
C	-4.613362	5.372192	-1.337236
H	7.661670	-0.758357	0.336268
H	7.842160	-3.208810	0.616147
H	5.842410	-4.610034	0.668002
H	4.223269	-5.708496	0.684815
H	2.189929	-7.041007	0.705003
H	-0.280239	-7.206611	0.580949
H	-2.477614	-6.130658	0.347185
H	-5.224364	-2.021258	-0.288679
H	-5.413669	0.393997	-0.472425
H	-3.381678	1.475436	0.651858
H	-1.472044	2.756160	0.629877
H	0.290116	4.212409	-0.511549
H	2.712493	4.374097	-0.481800
H	4.906048	3.312271	-0.279551
H	-3.307355	1.284333	-2.377306
H	-1.357141	2.579621	-2.398750
H	8.978016	2.690849	-2.162121
H	8.771425	3.195680	2.088871
H	-6.247013	-6.002856	-2.112824
H	-6.590056	-5.575465	2.137609
H	-5.036382	-4.392440	3.391867
H	-3.377904	-4.348102	2.769237
H	-4.422527	-2.945226	2.575972
H	-2.962199	-4.903683	-2.463049
H	-4.508392	-5.046870	-3.316566
H	-3.966333	-3.477802	-2.698134
H	-8.383665	-6.615440	-0.919041
H	-7.543635	-7.828210	0.049795
H	-8.453398	-6.543650	0.850658
H	7.084758	2.242173	3.367627
H	5.501751	2.243236	2.572848
H	6.386086	0.732649	2.755952
H	5.753610	1.602251	-2.714006
H	7.404413	1.455958	-3.339684
H	6.653291	0.110304	-2.464907
H	10.894479	3.650180	-0.950994
H	10.035676	4.959464	-0.134241
H	10.885416	3.741516	0.819250
H	-7.915468	10.159078	3.053686
H	-7.879964	8.468600	3.578651
H	-6.403321	9.442190	3.631636
H	-6.436413	9.615149	1.107396
H	-7.899831	8.645041	1.056488
H	-6.656170	6.716512	2.088878
H	-5.191743	7.687813	2.134638
H	-5.248778	7.889396	-0.374008
H	-6.712833	6.915116	-0.417339
H	-5.460572	4.984527	0.605340
H	-3.989128	5.963045	0.639282
H	-4.050173	6.151555	-1.857748
H	-5.534675	5.166013	-1.889266

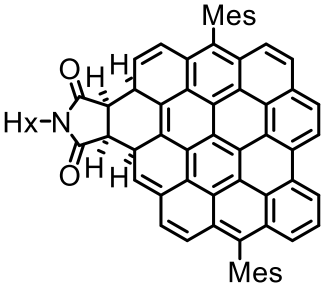

Int1-endo

Charge = 0; Multiplicity = 1

	x	y	z
C	3.037426	-3.235996	1.747062
C	1.630507	-3.602972	1.836262
C	0.670294	-2.875512	1.089499
C	1.077606	-1.782697	0.251786
C	2.445080	-1.416987	0.167275
C	3.426681	-2.141962	0.913687
C	0.106188	-1.061462	-0.494639
C	-1.276139	-1.416863	-0.418513
C	-2.239848	-0.700975	-1.166456
C	-1.810384	0.376443	-1.996044
C	-0.477905	0.727421	-2.069930
C	0.510645	0.021008	-1.323456
C	4.806376	-1.762974	0.821464
C	5.759434	-2.488302	1.556328
C	5.373945	-3.554889	2.362397
C	4.038265	-3.923841	2.458961
C	1.175798	-4.671248	2.653494

C	-0.154425	-5.006544	2.733716
C	-1.133619	-4.297269	1.998792
C	-0.716516	-3.222565	1.166904
C	-2.518660	-4.621542	2.066984
C	-3.449599	-3.922347	1.347921
C	-3.070540	-2.839519	0.495269
C	-1.690759	-2.496131	0.415127
C	-4.037221	-2.104173	-0.264585
C	-3.627287	-1.053476	-1.082780
C	-4.578965	-0.267757	-1.866028
C	-4.213194	0.766333	-2.638328
C	-2.785177	1.187242	-2.817190
C	-0.110658	1.884268	-2.969785
C	1.345259	2.222028	-2.949758
C	2.267150	1.522681	-2.241425
C	1.879768	0.388955	-1.398273
C	3.674930	1.868184	-2.282096
C	4.599047	1.178145	-1.579809
C	4.243981	0.049986	-0.734834
C	2.843053	-0.322728	-0.662579
C	5.196318	-0.648675	-0.016111
C	-5.490759	-2.467176	-0.160523
C	6.645641	-0.262923	-0.086263
C	-2.541070	2.717271	-2.574723
C	-1.048449	3.113222	-2.680547
C	-2.963031	3.157224	-1.170156
C	-0.725824	3.782981	-1.343649
O	-4.064736	3.058089	-0.668819
O	0.324707	4.295584	-1.014732
C	7.482062	-0.824471	-1.074217
C	8.829626	-0.451446	-1.119177
C	9.375529	0.459721	-0.211050
C	8.529392	1.006671	0.757516
C	7.176025	0.662316	0.838336
C	-6.032935	-3.468470	-0.993648
C	-7.387489	-3.796507	-0.871464
C	-8.221790	-3.159153	0.050598
C	-7.664305	-2.171156	0.867134
C	-6.315752	-1.810740	0.778151
C	-5.764689	-0.726825	1.678125
C	-5.173959	-4.184618	-2.011959
C	-9.689047	-3.508714	0.145273
C	6.301489	1.289441	1.901013
C	6.940251	-1.809705	-2.086096
C	10.841554	0.823456	-0.258890
N	-1.859365	3.722804	-0.538065
C	-3.211670	9.811359	3.832754
C	-2.821425	8.331017	3.774319
C	-2.757084	7.778558	2.344762
C	-2.364354	6.296970	2.280350
C	-2.306727	5.747999	0.849605
C	-1.907320	4.267885	0.817403
H	6.804817	-2.207727	1.490254
H	6.124776	-4.105206	2.923122
H	3.777901	-4.759465	3.097836
H	1.886257	-5.244573	3.237404
H	-0.469677	-5.829292	3.370921
H	-2.828897	-5.442898	2.708592
H	-4.499558	-4.185402	1.418470
H	-5.627166	-0.539150	-1.795435
H	-4.966054	1.329618	-3.184425
H	1.666675	3.069009	-3.551408
H	3.971648	2.712429	-2.899625
H	5.644583	1.462595	-1.630971
H	-3.158020	3.274558	-3.288911
H	-0.878273	3.855577	-3.468067
H	9.468171	-0.885579	-1.886501
H	8.931347	1.724049	1.470709
H	-7.800141	-4.571905	-1.514362
H	-8.295099	-1.665390	1.595985
H	-6.526090	-0.385169	2.386086
H	-4.901067	-1.080421	2.253202
H	-5.427537	0.143486	1.101897
H	-4.333547	-4.705435	-1.538117
H	-5.759927	-4.924677	-2.565811
H	-4.742228	-3.484504	-2.737078
H	-9.878827	-4.535422	-0.185474
H	-10.059810	-3.408892	1.171266
H	-10.298175	-2.846233	-0.484510
H	6.884410	1.958463	2.541590
H	5.485454	1.872520	1.458047
H	5.836319	0.529473	2.539659
H	6.168890	-1.353878	-2.718197
H	7.738156	-2.175452	-2.739771
H	6.478120	-2.675888	-1.598388
H	11.014639	1.840393	0.109506
H	11.441189	0.146378	0.364507
H	11.237550	0.760423	-1.278171
H	-3.246351	10.175269	4.865964
H	-2.492819	10.432902	3.284696
H	-4.200123	9.979219	3.387418
H	-3.540456	7.739469	4.358710

H	-1.845736	8.190207	4.260919
H	-2.038549	8.370578	1.758810
H	-3.734004	7.917226	1.858132
H	-3.081428	5.705584	2.868494
H	-1.385436	6.159710	2.762898
H	-1.583110	6.323001	0.257660
H	-3.286241	5.861369	0.367160
H	-2.627491	3.662730	1.374907
H	-0.912866	4.127979	1.250183
H	-2.530306	1.050375	-3.885833

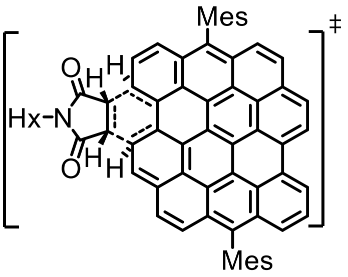

TS1

Charge = 0; Multiplicity = 1

	x	y	z
C	4.126293	-3.313945	0.206309
C	2.848937	-4.022181	0.231746
C	1.637176	-3.281431	0.221307
C	1.660622	-1.847635	0.195454
C	2.908205	-1.154239	0.162710
C	4.134713	-1.884098	0.164479
C	0.445836	-1.119321	0.200071
C	-0.812457	-1.804141	0.202097
C	-2.027880	-1.070242	0.170021
C	-1.998479	0.356242	0.192347
C	-0.757807	1.037768	0.233217
C	0.469287	0.304524	0.190346
C	5.380658	-1.173552	0.124968
C	6.585012	-1.911050	0.135076
C	6.565579	-3.295154	0.181943
C	5.355375	-3.988451	0.216026
C	2.770419	-5.433709	0.261318
C	1.560091	-6.092903	0.273128
C	0.342508	-5.382721	0.255519
C	0.378924	-3.961090	0.232515
C	-0.922462	-6.045736	0.254702
C	-2.091703	-5.343081	0.224940
C	-2.100957	-3.909446	0.199656
C	-0.845430	-3.223256	0.213130
C	-3.314151	-3.173608	0.158012
C	-3.282411	-1.769217	0.136270
C	-4.496121	-0.983982	0.095638
C	-4.477662	0.368503	0.062647
C	-3.224263	1.120175	0.011765
C	-0.709025	2.465420	0.185720
C	0.526127	3.134722	0.124708
C	1.722722	2.432540	0.080700
C	1.711496	0.992862	0.132940
C	2.993839	3.104334	0.017651
C	4.160808	2.413328	0.013614
C	4.190996	0.968404	0.068696
C	2.930194	0.265453	0.120506
C	5.391294	0.262608	0.073600
C	-4.628168	-3.900478	0.137262
C	6.704586	0.987885	0.028103
C	-3.095264	1.930359	-1.683095
C	-1.860339	2.618754	-1.912714
C	-4.155031	3.026254	-1.613102
C	-2.095326	4.061220	-1.895240
O	-5.363204	2.894988	-1.516350
O	-1.320006	4.992086	-2.054790
C	7.307504	1.282160	-1.213709
C	8.533916	1.954581	-1.231754
C	9.183606	2.341294	-0.056320
C	8.567278	2.043251	1.161927
C	7.341047	1.373107	1.227366
C	-5.220272	-4.258694	-1.092458
C	-6.444735	-4.935228	-1.087171
C	-7.102522	-5.265320	0.100697
C	-6.496453	-4.905608	1.307566
C	-5.273114	-4.228600	1.349039
C	-4.663128	-3.859907	2.683264
C	-4.553033	-3.922615	-2.407661
C	-8.440808	-5.966739	0.080909

C	6.715624	1.080044	2.573149
C	6.645577	0.889664	-2.515871
C	10.523890	3.037724	-0.101398
N	-3.481724	4.237278	-1.634398
C	-5.564769	9.857764	3.060441
C	-5.531720	9.333171	1.621358
C	-4.866924	7.956269	1.494970
C	-4.828461	7.424356	0.056680
C	-4.160271	6.049063	-0.061959
C	-4.122849	5.538149	-1.508045
H	7.529265	-1.378926	0.105416
H	7.500120	-3.849393	0.190559
H	5.384470	-5.071143	0.249753
H	3.677301	-6.026819	0.271919
H	1.535416	-7.179694	0.292763
H	-0.937469	-7.132824	0.273889
H	-3.041663	-5.866440	0.219112
H	-5.443708	-1.512171	0.106798
H	-5.403027	0.932468	0.020748
H	-3.267546	2.051765	0.577472
H	-1.572156	3.036101	0.503263
H	0.538045	4.220150	0.099994
H	2.996936	4.190385	-0.027060
H	5.106788	2.941821	-0.034353
H	-3.356099	1.069425	-2.293037
H	-0.990418	2.207992	-2.403262
H	8.992275	2.182444	-2.192321
H	9.051891	2.340744	2.089979
H	-6.895367	-5.210864	-2.038796
H	-6.987950	-5.157896	2.245403
H	-5.298768	-4.195743	3.508293
H	-3.672993	-4.312185	2.814859
H	-4.530470	-2.775835	2.780037
H	-3.550215	-4.360310	-2.476190
H	-5.142460	-4.296393	-3.250441
H	-4.435190	-2.839781	-2.534181
H	-8.551581	-6.594427	-0.809737
H	-8.573653	-6.602512	0.962919
H	-9.268331	-5.244555	0.074237
H	7.366636	1.414994	3.386424
H	5.748631	1.584299	2.687302
H	6.531224	0.007869	2.708193
H	5.673283	1.381450	-2.639043
H	7.271690	1.165621	-3.369818
H	6.462600	-0.190056	-2.565870
H	10.667287	3.688379	0.767830
H	11.349272	2.312996	-0.104260
H	10.627678	3.649543	-1.003925
H	-6.043919	10.841844	3.117008
H	-6.121244	9.178126	3.717664
H	-4.551674	9.956425	3.469628
H	-4.999743	10.051745	0.981782
H	-6.556758	9.277441	1.228225
H	-5.399523	7.236817	2.134578
H	-3.841135	8.011453	1.888711
H	-4.295340	8.143702	-0.582217
H	-5.853994	7.366999	-0.336972
H	-4.697664	5.319246	0.558511
H	-3.131727	6.101746	0.319123
H	-3.560853	6.231191	-2.141443
H	-5.137277	5.437807	-1.905926

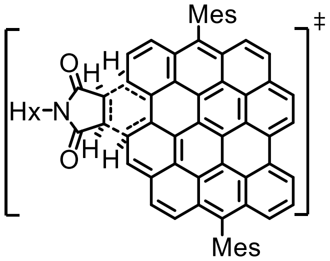

TS1-endo

Charge = 0; Multiplicity = 1

	x	y	z
C	2.590508	-2.826101	2.061428
C	1.149956	-3.043051	2.166979
C	0.275740	-2.399127	1.251443
C	0.798449	-1.548424	0.221753
C	2.205773	-1.332397	0.119872
C	3.097146	-1.965688	1.037150
C	-0.082725	-0.918514	-0.691667
C	-1.498440	-1.105480	-0.581799
C	-2.380243	-0.438293	-1.472065

C	-1.852558	0.387041	-2.508284
C	-0.452101	0.527175	-2.661083
C	0.437338	-0.086067	-1.722917
C	4.508705	-1.732393	0.927350
C	5.376916	-2.369303	1.841361
C	4.876195	-3.203983	2.826695
C	3.503869	-3.429736	2.937465
C	0.583595	-3.874156	3.161278
C	-0.778141	-4.060185	3.262090
C	-1.669373	-3.425561	2.372711
C	-1.138135	-2.589462	1.352131
C	-3.084473	-3.589778	2.470486
C	-3.933318	-2.954780	1.610944
C	-3.440336	-2.104868	0.567451
C	-2.025741	-1.936086	0.441871
C	-4.319620	-1.432552	-0.322542
C	-3.799255	-0.607667	-1.330540
C	-4.663901	0.088896	-2.258126
C	-4.175856	0.935844	-3.188244
C	-2.743783	1.233101	-3.300940
C	0.093839	1.364907	-3.681178
C	1.476909	1.580776	-3.757777
C	2.349120	1.012771	-2.837000
C	1.836794	0.143257	-1.810883
C	3.769167	1.227475	-2.905732
C	4.618860	0.631190	-2.032595
C	4.143440	-0.248593	-0.988576
C	2.721331	-0.479790	-0.892411
C	5.019006	-0.863280	-0.096599
C	-5.804788	-1.585118	-0.166253
C	6.497649	-0.624934	-0.193275
C	-2.450346	2.961304	-2.796640
C	-1.110818	3.426174	-3.034226
C	-2.610415	2.982166	-1.278220
C	-0.425380	3.613456	-1.764727
O	-3.627986	2.793610	-0.633686
O	0.702232	4.017622	-1.521383
C	7.301715	-1.476330	-0.981376
C	8.678371	-1.239357	-1.051425
C	9.282903	-0.181631	-0.366220
C	8.468806	0.646741	0.410531
C	7.087674	0.446897	0.509952
C	-6.484718	-2.629736	-0.826147
C	-7.869810	-2.748631	-0.665482
C	-8.599456	-1.859957	0.127875
C	-7.904440	-0.833938	0.775458

C	-6.521112	-0.676263	0.643742
C	-5.816348	0.449813	1.367295
C	-5.740756	-3.614565	-1.700779
C	-10.099184	-1.986836	0.264504
C	6.253117	1.376427	1.362994
C	6.697338	-2.632642	-1.746509
C	10.767845	0.073238	-0.482374
N	-1.356098	3.233712	-0.754201
C	-1.125679	7.447850	5.588135
C	-0.886378	6.079197	4.942458
C	-1.217908	6.049991	3.444776
C	-0.979701	4.682092	2.792527
C	-1.308184	4.658550	1.294690
C	-1.056977	3.279889	0.669724
H	6.444534	-2.196609	1.762957
H	5.555864	-3.687893	3.522762
H	3.153863	-4.087272	3.724551
H	1.224201	-4.382234	3.872531
H	-1.180560	-4.702784	4.041385
H	-3.478582	-4.231150	3.255141
H	-5.005772	-3.084284	1.708308
H	-5.732064	-0.084841	-2.187126
H	-4.854482	1.451700	-3.863522
H	1.878373	2.200298	-4.555077
H	4.150951	1.888268	-3.679750
H	5.686368	0.809561	-2.102890
H	-3.268098	3.434702	-3.339359
H	-0.758361	3.909669	-3.934754
H	9.293694	-1.901136	-1.658300
H	8.918860	1.473084	0.957536
H	-8.390183	-3.557425	-1.175156
H	-8.453216	-0.133612	1.402605
H	-6.531926	1.050933	1.936973
H	-5.067940	0.064855	2.070979
H	-5.287139	1.117478	0.677618
H	-4.977618	-4.162817	-1.135532
H	-6.427749	-4.347136	-2.135728
H	-5.220958	-3.109986	-2.523909
H	-10.620880	-1.295537	-0.410959
H	-10.439723	-2.998865	0.021771
H	-10.430959	-1.753070	1.282357
H	6.881840	2.129782	1.847241
H	5.497544	1.901733	0.767131
H	5.714704	0.831383	2.147226
H	5.963332	-2.287599	-2.484483
H	7.470814	-3.194235	-2.279232

H	6.173033	-3.328062	-1.080610	H	-2.268123	6.343816	3.300066
H	10.995763	0.711354	-1.346821	H	-1.584431	3.923299	3.311240
H	11.158180	0.580268	0.406438	H	0.070303	4.388614	2.939473
H	11.326395	-0.859906	-0.613374	H	-0.695263	5.399062	0.765473
H	-0.878148	7.436177	6.655911	H	-2.359383	4.932367	1.137374
H	-0.512300	8.223023	5.112354	H	-1.677435	2.520825	1.156038
H	-2.175062	7.753062	5.492877	H	-0.005338	2.999897	0.781906
H	-1.488350	5.319503	5.461295	H	-2.435393	1.361348	-4.339845
H	0.163072	5.784838	5.086343	H	-0.508119	1.604279	-4.547507
H	-0.615484	6.809634	2.924984				

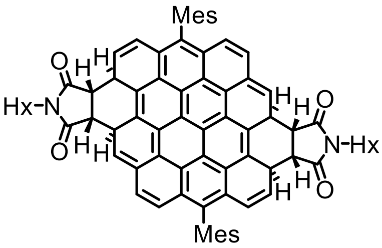

Int2

Charge = 0; Multiplicity = 1

	x	y	z				
C	-3.434139	-0.846438	-0.115814	C	4.914615	1.156350	-0.087338
C	-3.059933	0.479076	-0.102217	C	4.152769	-1.525292	-0.063552
C	-1.679561	0.854245	-0.124022	C	3.646667	-2.932116	-0.142850
C	-0.688955	-0.173631	-0.132175	C	2.322958	-3.248730	-0.169962
C	-1.074732	-1.539420	-0.147031	C	1.294683	-2.210452	-0.147732
C	-2.453500	-1.890777	-0.151496	C	1.870451	-4.625013	-0.219859
C	0.688935	0.173797	-0.132143	C	0.554460	-4.945979	-0.236104
C	1.074715	1.539585	-0.147026	C	-0.484164	-3.936031	-0.209320
C	2.453484	1.890942	-0.151424	C	-0.080437	-2.564363	-0.165781
C	3.434119	0.846604	-0.115669	C	-1.854302	-4.271737	-0.232782
C	3.059909	-0.478909	-0.102048	C	2.269305	5.714770	-0.297067
C	1.679540	-0.854079	-0.123902	C	-2.269303	-5.714615	-0.297005
C	-2.837323	-3.255832	-0.203211	C	5.641979	0.294843	-1.181038
C	-4.262698	-3.563913	-0.242339	C	5.214825	-1.192379	-1.175579
C	-5.220797	-2.622016	-0.200425	C	7.156447	0.267038	-0.972917
C	-3.646681	2.932286	-0.143078	C	6.513607	-1.972799	-0.976057
C	-2.322971	3.248895	-0.170187	O	7.915611	1.214656	-0.901546
C	-1.294701	2.210616	-0.147879	O	6.655708	-3.179287	-0.910761
C	-1.870462	4.625175	-0.220154	C	-2.437683	-6.345904	-1.547767
C	-0.554470	4.946139	-0.236369	C	-2.826845	-7.688946	-1.586818
C	0.484151	3.936192	-0.209452	C	-3.052099	-8.427598	-0.422108
C	0.080421	2.564527	-0.165873	C	-2.883938	-7.782396	0.806089
C	1.854291	4.271897	-0.232823	C	-2.496436	-6.440960	0.891200
C	2.837310	3.255995	-0.203143	C	2.438347	6.345846	-1.547869
C	4.262690	3.564074	-0.242159	C	2.827555	7.688853	-1.586957
C	5.220787	2.622177	-0.200197	C	3.052198	8.427723	-0.422243
C				C	2.883372	7.782751	0.805963

C	2.495820	6.441313	0.891112	H	-2.878485	-4.725605	-2.922654
C	2.331853	5.790399	2.246578	H	-4.003715	-10.110687	-1.400669
C	2.211847	5.590882	-2.838932	H	-2.555192	-10.535674	-0.485158
C	3.441274	9.886448	-0.488352	H	-4.057448	-10.176127	0.369796
C	-2.333133	-5.789807	2.246631	H	13.953403	-3.018146	4.428278
C	-2.210500	-5.591189	-2.838855	H	12.765383	-1.766847	4.824259
C	-3.441136	-9.886341	-0.488064	H	12.277475	-3.467261	4.780125
N	7.559921	-1.061289	-0.863812	H	12.747360	-3.529825	2.294841
C	12.909123	-2.715056	4.291661	H	13.227449	-1.840790	2.339126
C	12.557321	-2.575882	2.807098	H	10.911040	-1.201142	3.081010
C	11.101449	-2.155752	2.568440	H	10.430392	-2.891325	3.036131
C	10.741639	-2.014248	1.083839	H	10.929407	-2.969509	0.571854
C	9.285024	-1.592982	0.854464	H	11.411971	-1.278399	0.615893
C	8.947928	-1.461365	-0.635522	H	9.093505	-0.630702	1.346965
H	-4.547405	-4.609163	-0.310536	H	8.608428	-2.329783	1.306414
H	-6.269618	-2.896954	-0.241949	H	9.091092	-2.416435	-1.148685
H	-4.394591	3.718469	-0.172093	H	9.577180	-0.702298	-1.108199
H	-2.626144	5.406464	-0.240907	C	-4.914633	-1.156191	-0.087529
H	-0.251781	5.987388	-0.270904	H	-5.319203	-0.798086	0.879858
H	4.547404	4.609327	-0.310306	C	-4.152791	1.525466	-0.063736
H	6.269609	2.897117	-0.241640	H	-4.699180	1.412427	0.894760
H	5.319153	0.798233	0.880056	C	-5.641964	-0.294689	-1.181250
H	4.699186	-1.412227	0.894924	C	-5.214896	1.192556	-1.175717
H	4.394579	-3.718299	-0.171824	H	-5.443435	-0.759388	-2.152235
H	2.626135	-5.406303	-0.240524	H	-4.786293	1.488271	-2.138192
H	0.251774	-5.987231	-0.270568	C	-7.156451	-0.266990	-0.973266
H	5.443538	0.759580	-2.152024	C	-6.513711	1.972875	-0.976000
H	4.786138	-1.488006	-2.138042	O	-6.655924	3.179359	-0.910852
H	-2.958455	-8.168798	-2.554901	O	-7.915513	-1.214672	-0.901684
H	-3.060718	-8.336492	1.726199	N	-7.559983	1.061299	-0.863914
H	2.959685	8.168528	-2.555061	C	-8.948003	1.461268	-0.635510
H	3.059657	8.337016	1.726064	H	-9.577232	0.702253	-1.108300
H	2.532123	6.506420	3.049588	H	-9.091234	2.416423	-1.148496
H	1.317150	5.399796	2.386810	C	-9.285045	1.592603	0.854514
H	3.016278	4.942908	2.372270	H	-9.093455	0.630244	1.346834
H	1.187523	5.206070	-2.907132	H	-8.608467	2.329355	1.306568
H	2.387743	6.236897	-3.704543	C	-10.741671	2.013749	1.084036
H	2.880278	4.725611	-2.922468	H	-10.929516	2.969094	0.572235
H	4.007886	10.109719	-1.398738	H	-11.411988	1.277949	0.615990
H	2.555211	10.535622	-0.490328	C	-11.101411	2.154961	2.568682
H	4.053696	10.177447	0.371864	H	-10.910923	1.200268	3.081066
H	-2.533920	-6.505655	3.049666	H	-10.430370	2.890485	3.036473
H	-1.318455	-5.399303	2.387336	C	-12.557293	2.574968	2.807496
H	-3.017519	-4.942208	2.371801	H	-12.747412	3.528995	2.295425
H	-1.185968	-5.206882	-2.906846	H	-13.227407	1.839925	2.339425
H	-2.386478	-6.237217	-3.704440	C	-12.909022	2.713849	4.292104

H	-13.953312	3.016857	4.428833
H	-12.765203	1.765549	4.824519
H	-12.277389	3.465998	4.780672

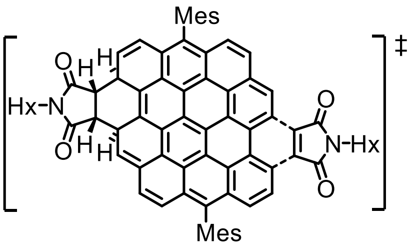

TS2

Charge = 0; Multiplicity = 1

	x	y	z
C	3.488295	0.921950	0.277017
C	3.113242	-0.433312	0.347856
C	1.738012	-0.803984	0.239508
C	0.739863	0.213115	0.160594
C	1.115761	1.579225	0.140284
C	2.495915	1.939351	0.171643
C	-0.632956	-0.147822	0.086665
C	-1.009540	-1.520034	0.060703
C	-2.379533	-1.880855	-0.020132
C	-3.368213	-0.847679	-0.047918
C	-3.004137	0.482427	-0.023375
C	-1.629534	0.867060	0.028137
C	2.867903	3.315891	0.108756
C	4.272680	3.641278	0.139588
C	5.235702	2.689244	0.185997
C	3.722602	-2.820849	0.361454
C	2.381302	-3.187564	0.237248
C	1.365339	-2.170864	0.195184
C	1.963372	-4.557857	0.186892
C	0.646098	-4.899900	0.097352
C	-0.393010	-3.907732	0.049440
C	-0.010156	-2.536571	0.102884
C	-1.768983	-4.258661	-0.053488
C	-2.750379	-3.257153	-0.087826
C	-4.171117	-3.573275	-0.212483
C	-5.134044	-2.637534	-0.235350
C	-4.845851	-1.168002	-0.112321
C	-4.103474	1.520492	-0.059298
C	-3.604492	2.930281	-0.125217
C	-2.284844	3.259403	-0.084182
C	-1.250801	2.230846	0.007309
C	-1.846295	4.639710	-0.130765
C	-0.534637	4.974229	-0.089202
C	0.510688	3.975067	0.002065
C	0.115551	2.593118	0.052811
C	1.869343	4.325401	0.031645
C	-2.158958	-5.707095	-0.135437
C	2.274833	5.771127	-0.022864
C	-5.499237	-0.321546	-1.261535
C	-5.095236	1.172422	-1.228064
C	-7.026009	-0.313499	-1.178344
C	-6.415460	1.934183	-1.109923
O	-7.776208	-1.270492	-1.176777
O	-6.574613	3.138043	-1.034984
C	2.518058	6.390890	-1.267199
C	2.890575	7.738642	-1.295460
C	3.034178	8.491133	-0.126411
C	2.785353	7.859016	1.094774
C	2.410061	6.513395	1.169570
C	-2.229975	-6.348658	-1.390080
C	-2.589943	-7.699623	-1.444714
C	-2.879317	-8.435150	-0.292733
C	-2.807886	-7.778953	0.939441
C	-2.452312	-6.430191	1.040624
C	-2.394143	-5.767671	2.399215
C	-1.931965	-5.598744	-2.669662
C	-3.234950	-9.901628	-0.372164
C	2.152112	5.879072	2.518420
C	2.378109	5.621965	-2.562164
C	3.470564	9.936799	-0.180479
N	-7.454776	1.009246	-1.094990
C	-13.364626	2.390530	3.505739
C	-12.845672	2.315103	2.066342
C	-11.363357	1.929876	1.978310
C	-10.838534	1.851300	0.539033
C	-9.357557	1.461314	0.455711
C	-8.864068	1.387713	-0.993981
H	4.553814	4.689302	0.136796
H	6.284226	2.966530	0.193990

H	4.484332	-3.592285	0.421175
H	2.727519	-5.330255	0.218735
H	0.360594	-5.945705	0.057820
H	-4.443677	-4.620813	-0.292989
H	-6.176387	-2.919523	-0.342029
H	-5.316406	-0.807177	0.822783
H	-4.701536	1.418430	0.868120
H	-4.357264	3.708494	-0.205132
H	-2.608333	5.411781	-0.200658
H	-0.239348	6.017279	-0.127394
H	-5.217458	-0.786120	-2.211792
H	-4.617066	1.473621	-2.165541
H	3.072614	8.212001	-2.258488
H	2.884928	8.427425	2.017622
H	-2.646296	-8.188172	-2.415637
H	-3.036026	-8.330675	1.849634
H	-2.615110	-6.487477	3.193304
H	-1.405123	-5.338454	2.597798
H	-3.116791	-4.946605	2.479122
H	-0.913598	-5.192638	-2.673288
H	-2.035066	-6.255243	-3.538995
H	-2.611631	-4.749263	-2.806631
H	-2.349166	-10.534579	-0.226712
H	-3.960902	-10.180712	0.399371
H	-3.661245	-10.158698	-1.347534
H	2.324909	6.597134	3.325843
H	1.120725	5.516890	2.604721
H	2.805576	5.015607	2.689795
H	1.366060	5.218772	-2.685615
H	2.592501	6.263882	-3.421984
H	3.065820	4.768583	-2.600141
H	4.565352	10.022563	-0.160429
H	3.124740	10.426411	-1.097217
H	3.085413	10.504397	0.673343
H	-14.424898	2.666090	3.534489
H	-13.257101	1.425893	4.016968
H	-12.810629	3.136729	4.088515
H	-12.997886	3.284553	1.570872
H	-13.442757	1.586893	1.499273
H	-11.210628	0.959684	2.473970
H	-10.764608	2.658587	2.544483
H	-10.988092	2.822372	0.044460
H	-11.438507	1.123478	-0.026694
H	-9.201657	0.485281	0.933238
H	-8.747407	2.192210	1.002025
H	-8.965958	2.359226	-1.485511
H	-9.432110	0.640924	-1.555664
C	4.919321	1.253575	0.148591
H	5.545501	0.696182	0.853280
C	4.110996	-1.471528	0.391902
H	5.085677	-1.240445	0.806370
C	5.498759	0.560021	-1.358557
C	5.125132	-0.826662	-1.588078
H	5.152201	1.291283	-2.088510
H	4.263527	-1.142092	-2.158492
C	7.027056	0.525036	-1.218495
C	6.315361	-1.664927	-1.544805
O	6.445131	-2.868846	-1.712702
O	7.780008	1.473262	-1.072479
N	7.407521	-0.802921	-1.236692
C	8.775973	-1.269500	-1.063292
H	9.435153	-0.462978	-1.398236
H	8.904208	-2.131216	-1.725455
C	9.096515	-1.662288	0.384757
H	8.930554	-0.792867	1.035142
H	8.391684	-2.442397	0.701289
C	10.535847	-2.166189	0.546838
H	10.696547	-3.026609	-0.119255
H	11.235247	-1.385705	0.212474
C	10.878320	-2.570152	1.986606
H	10.719195	-1.709658	2.653396
H	10.176642	-3.348304	2.321993
C	12.315629	-3.079984	2.153366
H	12.474211	-3.938827	1.485808
H	13.016612	-2.301945	1.819052
C	12.649735	-3.483733	3.593020
H	13.681943	-3.841947	3.679363
H	12.532730	-2.636205	4.279689
H	11.988452	-4.285879	3.943466

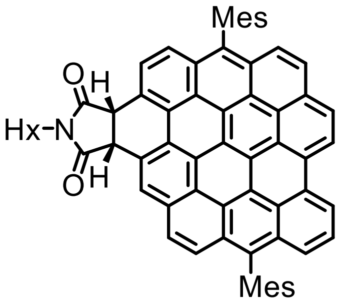

3a'

Charge = 0; Multiplicity = 1

	x	y	z
C	2.596958	-3.98976	1.181189
C	1.154309	-4.23543	1.259983
C	0.244472	-3.26075	0.760614
C	0.725064	-2.04325	0.174437
C	2.142816	-1.81365	0.08076
C	3.066145	-2.77653	0.580727
C	-0.18624	-1.08101	-0.30869
C	-1.60329	-1.31648	-0.22297
C	-2.52469	-0.34218	-0.7
C	-2.05185	0.887956	-1.25978
C	-0.6161	1.115564	-1.38593
C	0.29459	0.131901	-0.90336
C	4.477671	-2.52491	0.482826
C	5.379709	-3.49125	0.997634
C	4.911856	-4.65133	1.579689
C	3.535909	-4.9003	1.670762
C	0.630428	-5.41718	1.817017
C	-0.732	-5.64705	1.890264
C	-1.65166	-4.70332	1.409443
C	-1.16364	-3.49314	0.837255
C	-3.06733	-4.92554	1.478434
C	-3.94992	-4.00463	1.011547
C	-3.50489	-2.76364	0.427634
C	-2.08638	-2.51867	0.344174
C	-4.4149	-1.81146	-0.04541
C	-3.93464	-0.59016	-0.60467
C	-4.82763	0.407161	-1.07574
C	-4.35464	1.592525	-1.59136
C	-2.97385	1.848867	-1.68252
C	-0.10974	2.277652	-2.00702
C	1.262915	2.461834	-2.13358
C	2.180445	1.528728	-1.63838
C	1.700211	0.342147	-1.01571
C	3.596797	1.739093	-1.74532
C	4.481933	0.831088	-1.2594
C	4.042021	-0.38818	-0.62539
C	2.624111	-0.62664	-0.51731
C	4.953745	-1.32276	-0.12511
C	-5.89273	-2.06252	0.033909
C	6.430787	-1.07175	-0.21912
C	-2.52285	3.189932	-2.19684
C	-1.0441	3.326054	-2.60616
C	-2.74416	4.327016	-1.17837
C	-0.66467	4.735663	-2.12576
O	-3.70926	4.503213	-0.46304
O	0.328649	5.373838	-2.42085
C	-6.55922	-2.69807	-1.03498
C	-7.9398	-2.91327	-0.94617
C	-8.67964	-2.51872	0.171585
C	-7.99987	-1.88828	1.218995
C	-6.62179	-1.65401	1.172488
C	7.155017	-1.51222	-1.3484
C	8.53247	-1.27632	-1.40908
C	9.215868	-0.61329	-0.38467
C	8.480571	-0.1873	0.724772
C	7.101133	-0.40253	0.826902
C	-5.80756	-3.14503	-2.26929
C	-5.93649	-0.96879	2.334229
C	-10.1653	-2.78226	0.258705
C	6.465463	-2.23173	-2.48674
C	6.354515	0.079661	2.050918
C	10.69993	-0.34666	-0.48718
N	-1.65589	5.186665	-1.26495
C	0.739446	8.682639	5.055354
C	0.036788	8.820753	3.700895
C	-0.06263	7.495044	2.935337
C	-0.76679	7.625127	1.578675
C	-0.85887	6.295135	0.820313
C	-1.56619	6.450382	-0.53085
H	6.445611	-3.30566	0.927911
H	5.612776	-5.38295	1.971994

H	3.214191	-5.82441	2.13601
H	1.297755	-6.17952	2.201051
H	-1.10147	-6.57191	2.325918
H	-3.42222	-5.85474	1.917359
H	-5.01675	-4.18892	1.073982
H	-5.89534	0.228662	-1.01704
H	-5.05405	2.351734	-1.92846
H	1.634474	3.361848	-2.61309
H	3.947716	2.650258	-2.22306
H	5.548373	1.008892	-1.34394
H	-8.44871	-3.40115	-1.7753
H	-8.55655	-1.56707	2.097518
H	9.08548	-1.62208	-2.28057
H	8.992468	0.326094	1.536497
H	-5.02605	-3.87359	-2.02322
H	-6.48558	-3.60945	-2.9917
H	-5.31022	-2.30334	-2.76575
H	-6.65829	-0.71267	3.115613
H	-5.16709	-1.60872	2.782309
H	-5.43659	-0.04495	2.020449
H	-10.5979	-2.96503	-0.73021
H	-10.3772	-3.66432	0.877605
H	-10.6963	-1.93754	0.711607
H	5.702015	-1.60114	-2.95781
H	7.185982	-2.5201	-3.25804
H	5.957068	-3.13987	-2.14229
H	7.035586	0.564038	2.757154
H	5.572616	0.801606	1.787273
H	5.858648	-0.74728	2.572848
H	11.22528	-1.18144	-0.96412
H	10.9023	0.54833	-1.09064
H	11.14616	-0.18254	0.499076
H	0.796776	9.645381	5.576154
H	0.206004	7.981128	5.708694
H	1.76325	8.307586	4.933662
H	0.573012	9.555709	3.083792
H	-0.97302	9.228123	3.85178
H	-0.59807	6.758863	3.55278
H	0.947751	7.087924	2.782436
H	-0.23178	8.360853	0.960493
H	-1.7784	8.028804	1.731095
H	-1.40358	5.557536	1.423457
H	0.147905	5.892136	0.650898
H	-1.02708	7.152462	-1.17267
H	-2.58901	6.810949	-0.39382
H	-0.92582	3.311022	-3.69559
H	-3.1545	3.467373	-3.05166

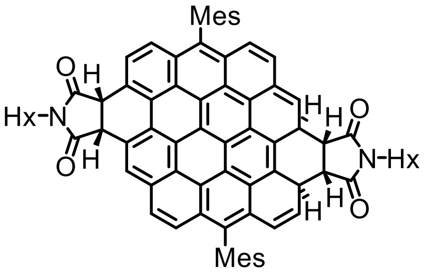

Int3

Charge = 0; Multiplicity = 1

	x	y	z
C	-3.47382	1.042151	0.824131
C	-3.12202	-0.36416	0.877595
C	-1.78482	-0.75676	0.615608
C	-0.78748	0.227125	0.299521
C	-1.123	1.603707	0.282542
C	-2.46453	2.01565	0.554496
C	0.546795	-0.17865	0.019319
C	0.90653	-1.56061	0.060677
C	2.233835	-1.9622	-0.21903
C	3.201054	-0.97043	-0.56579
C	2.86103	0.366654	-0.60216
C	1.533307	0.795705	-0.29883
C	-2.79157	3.408871	0.545493
C	-4.12089	3.785216	0.803326
C	-5.10065	2.829717	1.041648
C	-4.79829	1.468165	1.047204
C	-4.08055	-1.35969	1.223367
C	-3.70732	-2.68695	1.296841
C	-2.38816	-3.10753	1.017801
C	-1.41295	-2.13421	0.673123
C	-2.00393	-4.47901	1.076581

C	-0.71943	-4.86717	0.807592
C	0.290806	-3.91586	0.460707
C	-0.07171	-2.54098	0.398195
C	1.639866	-4.31072	0.186348
C	2.593711	-3.34886	-0.14286
C	3.98693	-3.70333	-0.40615
C	4.923297	-2.80266	-0.74252
C	4.629672	-1.33823	-0.90435
C	3.943251	1.358873	-0.97106
C	3.501365	2.787012	-0.89284
C	2.232391	3.163353	-0.5948
C	1.192379	2.174637	-0.29998
C	1.84934	4.562831	-0.55707
C	0.585448	4.948571	-0.27784
C	-0.47055	3.990289	0.004441
C	-0.1215	2.581094	-0.00881
C	-1.7652	4.392941	0.278239
C	2.01092	-5.7648	0.254797
C	-2.12848	5.849453	0.306505
C	-5.91231	0.473292	1.241536
C	-5.516	-0.97791	1.577663
C	-6.82225	0.344775	0.00284
C	-6.51858	-1.82314	0.775852
O	-7.23994	1.238714	-0.70469
O	-6.73602	-3.01513	0.888836
C	5.602396	-0.43152	-0.07286
C	5.216282	1.067553	-0.09742
C	7.032622	-0.47218	-0.60897
C	6.459133	1.781533	-0.62637
O	7.739523	-1.45084	-0.77214
O	6.613787	2.977638	-0.79883
C	-2.04233	6.57415	1.515092
C	-2.39465	7.928019	1.524618
C	-2.82884	8.587632	0.370654
C	-2.91197	7.850422	-0.81398
C	-2.56928	6.494495	-0.86868
C	2.457617	-6.32719	1.469162
C	2.794355	-7.68539	1.508418
C	2.69881	-8.50426	0.379958
C	2.257658	-7.92591	-0.81454
C	1.910866	-6.57346	-0.89837
C	-2.67437	5.744712	-2.17823
C	-1.57562	5.910526	2.79191
C	-3.17483	10.05848	0.399596
C	2.585639	-5.48529	2.7194
C	1.444712	-5.99847	-2.21778
C	3.039138	-9.97497	0.451001
N	-7.15074	-0.99761	-0.14347
C	-14.4548	-2.5136	-1.66444
C	-13.0111	-2.50718	-2.17736
C	-12.0045	-1.99462	-1.13928
C	-10.5563	-1.98848	-1.6454
C	-9.55653	-1.47572	-0.6014
C	-8.11749	-1.48427	-1.13
N	7.430169	0.826621	-0.90728
C	14.67009	2.435647	0.796315
C	13.62689	2.235755	-0.30788
C	12.23084	1.904441	0.235166
C	11.1805	1.700594	-0.8642
C	9.789934	1.366363	-0.3099
C	8.758694	1.157732	-1.42523
H	-4.38452	4.836975	0.806957
H	-6.12469	3.146015	1.216899
H	-4.44872	-3.43404	1.562356
H	-2.75546	-5.21891	1.339825
H	-0.44994	-5.91662	0.857299
H	4.260796	-4.74995	-0.31909
H	5.947519	-3.11368	-0.91988
H	4.807487	-1.0734	-1.96325
H	4.250871	1.15686	-2.0149
H	4.254324	3.541388	-1.09814
H	2.616987	5.303522	-0.76683
H	0.330342	6.002751	-0.2616
H	5.619572	-0.81453	0.95245
H	5.017691	1.441198	0.911712
H	-2.32924	8.480561	2.460161
H	-3.25491	8.341594	-1.72268
H	3.141817	-8.11238	2.447304
H	2.182078	-8.54293	-1.70803
H	-3.07145	6.391278	-2.96657
H	-1.69708	5.372384	-2.50807
H	-3.33305	4.87269	-2.092
H	-0.54792	5.539126	2.700666
H	-1.60567	6.613441	3.629971
H	-2.20157	5.048462	3.051073
H	-3.60071	10.35118	1.365413
H	-2.28357	10.67966	0.237766
H	-3.89611	10.31591	-0.38309
H	1.630788	-5.02152	2.993634
H	2.920825	-6.09287	3.565403
H	3.305955	-4.6695	2.584144
H	1.409515	-6.77334	-2.98953

H	0.444113	-5.55782	-2.13603	H	-7.81088	-2.49756	-1.40224
H	2.112558	-5.2029	-2.56911	H	15.65567	2.669802	0.377867
H	3.718988	-10.1889	1.282181	H	14.387	3.258774	1.464048
H	2.138121	-10.5849	0.600655	H	14.7742	1.532512	1.41032
H	3.512223	-10.322	-0.4742	H	13.95428	1.429534	-0.97962
H	-15.1488	-2.88359	-2.42782	H	13.56876	3.142843	-0.92611
H	-14.558	-3.1553	-0.78059	H	11.90213	2.711451	0.906573
H	-14.7785	-1.50466	-1.38014	H	12.2886	0.996964	0.854126
H	-12.9461	-1.88552	-3.08161	H	11.50851	0.893248	-1.53513
H	-12.7272	-3.52355	-2.48521	H	11.11885	2.608232	-1.48211
H	-12.0699	-2.6155	-0.23355	H	9.445747	2.175898	0.346716
H	-12.2866	-0.97655	-0.83256	H	9.841898	0.455777	0.300457
H	-10.4896	-1.3665	-2.54988	H	9.059197	0.335249	-2.07986
H	-10.2738	-3.00651	-1.9507	H	8.646894	2.064568	-2.02495
H	-9.60732	-2.09836	0.300987	H	-5.68016	-1.19867	2.63826
H	-9.82054	-0.45262	-0.30377	H	-6.58117	0.848169	2.027923
H	-8.02293	-0.8398	-2.008				

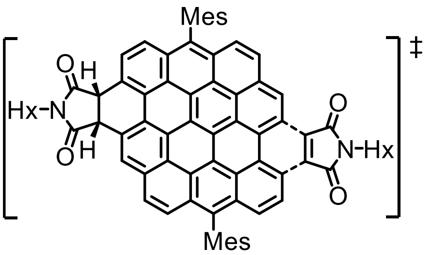

TS3

Charge = 0; Multiplicity = 1

	x	y	z				
				C	-2.23138	-3.11669	0.975733
C	-3.43517	1.002691	0.793758	C	-1.30578	-2.12752	0.54698
C	-3.0442	-0.39736	0.840539	C	-1.80843	-4.48055	1.040018
C	-1.71591	-0.76243	0.491219	C	-0.53668	-4.84348	0.702094
C	-0.76924	0.236861	0.089648	C	0.428957	-3.87539	0.269047
C	-1.14642	1.612941	0.074721	C	0.025781	-2.50534	0.19322
C	-2.4713	1.995706	0.436276	C	1.756646	-4.2458	-0.07397
C	0.5471	-0.1404	-0.2754	C	2.674835	-3.26408	-0.48025
C	0.949826	-1.51318	-0.22612	C	4.032759	-3.60325	-0.84786
C	2.279873	-1.88397	-0.56119	C	4.936076	-2.6631	-1.20405
C	3.207388	-0.89112	-0.9917	C	4.61734	-1.23034	-1.17995
C	2.801947	0.463785	-1.08549	C	3.727287	1.482114	-1.46807
C	1.484125	0.850665	-0.68479	C	3.350885	2.831074	-1.45903
C	-2.83395	3.381257	0.429457	C	2.076249	3.221424	-1.05789
C	-4.15715	3.730439	0.781706	C	1.111521	2.223003	-0.68037
C	-5.08543	2.759088	1.106018	C	1.677451	4.602773	-1.03582
C	-4.74403	1.399514	1.107863	C	0.423185	4.97185	-0.6701
C	-3.94817	-1.40505	1.263611	C	-0.57597	3.99968	-0.29024
C	-3.53497	-2.72541	1.333388	C	-0.20197	2.605557	-0.29877

C	-1.86774	4.380512	0.072375	H	-6.10179	3.048578	1.354373
C	2.172063	-5.68716	0.002967	H	-4.23919	-3.48438	1.659247
C	-2.26173	5.828617	0.092714	H	-2.52374	-5.23041	1.367724
C	-5.81779	0.385611	1.403893	H	-0.23551	-5.88357	0.760613
C	-5.36755	-1.05744	1.703491	H	4.313621	-4.65107	-0.84595
C	-6.82843	0.242869	0.246884	H	5.949581	-2.9457	-1.46581
C	-6.39941	-1.92394	0.961591	H	5.097088	-0.69069	-1.9981
O	-7.32841	1.133172	-0.40983	H	4.629734	1.212692	-1.99951
O	-6.56618	-3.1241	1.069178	H	4.068121	3.584863	-1.76954
C	5.537729	-0.46267	0.20233	H	2.410696	5.353515	-1.3184
C	5.252041	0.929637	0.42584	H	0.146839	6.020441	-0.65804
C	6.994895	-0.48397	-0.26737	H	5.309585	-1.17655	0.991587
C	6.387992	1.734568	0.022978	H	4.490651	1.322519	1.082746
O	7.699013	-1.46107	-0.47511	H	-2.36783	8.507745	2.191502
O	6.561385	2.948735	0.056572	H	-3.57914	8.244547	-1.91161
C	-2.10603	6.584563	1.275305	H	3.556596	-7.93053	2.158669
C	-2.48633	7.930459	1.276428	H	2.260322	-8.52031	-1.8841
C	-3.01424	8.551683	0.140049	H	-3.43679	6.270681	-3.12013
C	-3.16355	7.783489	-1.01776	H	-2.02137	5.280375	-2.73211
C	-2.79674	6.433425	-1.06445	H	-3.61905	4.771909	-2.19418
C	2.737912	-6.1981	1.191337	H	-0.5127	5.605076	2.377681
C	3.117378	-7.54351	1.241095	H	-1.51895	6.688935	3.350761
C	2.950651	-8.40064	0.148775	H	-2.12877	5.100149	2.859847
C	2.392412	-7.87342	-1.01896	H	-3.81591	10.3059	1.127088
C	1.998593	-6.53356	-1.11274	H	-2.513	10.65288	-0.0107
C	-2.97887	5.649022	-2.34503	H	-4.12222	10.25145	-0.61901
C	-1.53748	5.96332	2.532175	H	2.002662	-4.86587	2.739774
C	-3.39051	10.01508	0.160332	H	3.362741	-5.88747	3.234744
C	2.944147	-5.31469	2.40182	H	3.629997	-4.48685	2.185109
C	1.404307	-6.01569	-2.40424	H	1.346556	-6.81194	-3.1524
C	3.34131	-9.85773	0.23809	H	0.392627	-5.62025	-2.25464
N	-7.12826	-1.10621	0.109446	H	2.00364	-5.20019	-2.82624
C	-14.496	-2.74045	-0.90033	H	4.227956	-9.99689	0.866082
C	-13.0906	-2.71468	-1.50992	H	2.535091	-10.4588	0.679644
C	-12.0222	-2.19159	-0.54134	H	3.554744	-10.2764	-0.75086
C	-10.6132	-2.16066	-1.1473	H	-15.2351	-3.11863	-1.61582
C	-9.55055	-1.63679	-0.17326	H	-14.5313	-3.38419	-0.01265
C	-8.1547	-1.61013	-0.80633	H	-14.8128	-1.73602	-0.59317
N	7.374487	0.827897	-0.46593	H	-13.0954	-2.09013	-2.41458
C	14.54949	2.594124	1.366624	H	-12.8156	-3.72659	-1.83967
C	13.51284	2.432169	0.250017	H	-12.0151	-2.81731	0.363405
C	12.13747	1.986807	0.763492	H	-12.2973	-1.17924	-0.2104
C	11.09188	1.827192	-0.34773	H	-10.6199	-1.5332	-2.05047
C	9.719995	1.384478	0.175399	H	-10.337	-3.17253	-1.47777
C	8.685824	1.240898	-0.94901	H	-9.5223	-2.26838	0.723986
H	-4.44453	4.775767	0.787965	H	-9.81284	-0.62211	0.153144

H	-8.14004	-0.95968	-1.68489	H	11.45469	1.096878	-1.08603
H	-7.84533	-2.61459	-1.1064	H	10.986	2.78065	-0.88603
H	15.52027	2.911817	0.96892	H	9.34595	2.113709	0.905242
H	14.2275	3.344212	2.09961	H	9.811291	0.423035	0.696997
H	14.70214	1.650421	1.904899	H	9.014719	0.494878	-1.67879
H	13.87815	1.701861	-0.48602	H	8.550566	2.196102	-1.46458
H	13.40605	3.3829	-0.29151	H	-6.41967	0.753366	2.245574
H	11.77285	2.715839	1.50217	H	-5.46244	-1.28855	2.770356
H	12.24311	1.033745	1.302613				

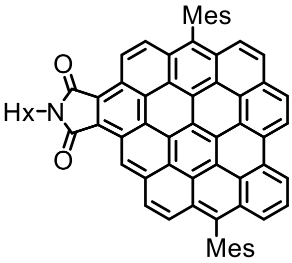

3a

Charge = 0; Multiplicity = 1

	x	y	z				
				C	-4.15731	-1.80703	-0.35384
C	4.694509	-2.63498	0.42936	C	-4.35761	-0.46739	-0.51858
C	3.547674	-3.54159	0.44563	C	-3.25315	0.436923	-0.532
C	2.236907	-3.01774	0.283437	C	-0.98288	2.233473	-0.55882
C	2.031455	-1.60916	0.105062	C	0.138329	3.091658	-0.57257
C	3.153289	-0.72139	0.088673	C	1.422657	2.595433	-0.42213
C	4.47613	-1.23226	0.248979	C	1.635647	1.183562	-0.24513
C	0.722335	-1.09907	-0.0538	C	2.570237	3.466282	-0.4376
C	-0.4077	-1.98344	-0.04034	C	3.82741	2.983088	-0.28958
C	-1.72185	-1.4718	-0.2003	C	4.083474	1.571152	-0.10677
C	-1.92532	-0.06618	-0.37401	C	2.952138	0.672385	-0.08768
C	-0.80098	0.82031	-0.38723	C	5.376546	1.078681	0.047469
C	0.518733	0.303101	-0.22918	C	-3.82735	-4.68292	-0.00171
C	5.59125	-0.32922	0.228836	C	6.555115	2.008139	0.026169
C	6.894088	-0.85243	0.390804	C	-3.39532	1.831746	-0.69462
C	7.092462	-2.21072	0.565439	C	-2.31433	2.69173	-0.70699
C	6.009278	-3.09159	0.584425	C	-4.63958	2.641867	-0.87407
C	3.694841	-4.93511	0.618302	C	-2.82231	4.082448	-0.89372
C	2.607881	-5.78497	0.631587	O	-5.80272	2.279961	-0.92025
C	1.29913	-5.29354	0.472774	O	-2.2075	5.133192	-0.95974
C	1.107934	-3.89462	0.296604	C	-4.28226	-5.26397	-1.20459
C	0.158918	-6.1574	0.483071	C	-5.38309	-6.12652	-1.16985
C	-1.10242	-5.6682	0.327957	C	-6.04625	-6.43241	0.021491
C	-1.34299	-4.26266	0.147207	C	-5.58205	-5.84255	1.200533
C	-0.21449	-3.37706	0.134139	C	-4.48532	-4.97467	1.21249
C	-2.64904	-3.75258	-0.01406	C	7.207972	2.293771	-1.19226
C	-2.8451	-2.36273	-0.18754	C	8.308642	3.1568	-1.18844

C	8.781603	3.747948	-0.01356	H	-2.54696	-5.2619	-2.50857
C	8.123484	3.448046	1.182132	H	-4.09136	-5.49809	-3.34305
C	7.019012	2.590496	1.224977	H	-3.62622	-3.89357	-2.75483
C	-3.60229	-4.96473	-2.52224	H	-4.64325	-4.709	3.350035
C	-4.02641	-4.36014	2.516279	H	-2.98353	-4.61536	2.73815
C	-7.21455	-7.39069	0.039025	H	-4.08523	-3.26556	2.489094
C	6.735692	1.682495	-2.49278	H	-7.74823	-7.38972	-0.9173
C	6.340917	2.302241	2.546024	H	-6.8819	-8.42091	0.224725
C	9.953104	4.70213	-0.04004	H	-7.93074	-7.13524	0.827396
N	-4.21707	3.970939	-0.99152	H	5.703892	1.9729	-2.72318
C	-8.74208	9.051808	2.426508	H	7.368846	2.001699	-3.32622
C	-8.20429	8.495874	1.104001	H	6.754888	0.586931	-2.45458
C	-7.18927	7.361439	1.294549	H	6.85711	2.808109	3.367669
C	-6.64388	6.800049	-0.02489	H	5.297736	2.640134	2.545469
C	-5.65438	5.645346	0.173388	H	6.325504	1.228463	2.766423
C	-5.12306	5.100188	-1.15857	H	10.66036	4.44681	-0.83649
H	7.739853	-0.17409	0.377514	H	9.621944	5.73394	-0.21924
H	8.099653	-2.59924	0.689211	H	10.49639	4.696828	0.910902
H	6.209406	-4.14744	0.723295	H	-9.46585	9.857385	2.257877
H	4.681641	-5.36457	0.745001	H	-9.24363	8.269947	3.010153
H	2.757678	-6.85336	0.766355	H	-7.93155	9.45643	3.045296
H	0.320289	-7.22417	0.618461	H	-7.73719	9.307481	0.528159
H	-1.95393	-6.33968	0.338684	H	-9.04189	8.132042	0.492067
H	-5.00608	-2.48216	-0.34661	H	-7.65851	6.548326	1.867994
H	-5.35476	-0.06085	-0.64245	H	-6.35201	7.723115	1.909679
H	-0.02583	4.155721	-0.70447	H	-6.15682	7.608427	-0.59015
H	2.400528	4.531364	-0.57266	H	-7.48339	6.45484	-0.64638
H	4.676742	3.657367	-0.30517	H	-6.14347	4.827752	0.718342
H	-5.73203	-6.56859	-2.10122	H	-4.80565	5.978749	0.784862
H	-6.08784	-6.06079	2.139275	H	-4.56982	5.873735	-1.6991
H	8.80964	3.371248	-2.13065	H	-5.95119	4.757601	-1.78672
H	8.478506	3.892007	2.11034				

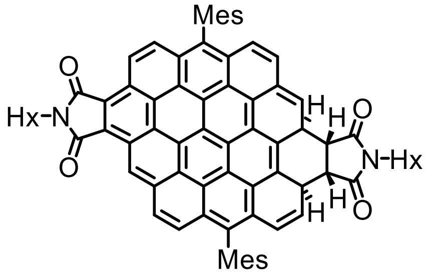

Int4

Charge = 0; Multiplicity = 1

	x	y	z				
C	3.472597	-0.921453	-0.446491	C	0.727526	-0.164049	-0.496772
C	3.114200	0.465482	-0.448528	C	1.079902	-1.536765	-0.491662
C	1.745629	0.842636	-0.470244	C	2.453543	-1.921774	-0.463696
				C	-0.640948	0.215632	-0.525353

C	-1.003241	1.594916	-0.513273
C	-2.368612	1.972257	-0.533236
C	-3.369706	0.953455	-0.589293
C	-3.021909	-0.381376	-0.605319
C	-1.652903	-0.784339	-0.561341
C	2.805073	-3.304950	-0.449954
C	4.181485	-3.653055	-0.426595
C	5.172715	-2.697161	-0.415835
C	4.846669	-1.321430	-0.423268
C	4.122684	1.492826	-0.425517
C	3.750988	2.842186	-0.420327
C	2.403659	3.221358	-0.435417
C	1.383259	2.217799	-0.462356
C	2.005074	4.596195	-0.422211
C	0.688663	4.955736	-0.429794
C	-0.359304	3.975624	-0.455497
C	0.008133	2.599822	-0.477559
C	-1.737819	4.344118	-0.453792
C	-2.727375	3.355960	-0.488314
C	-4.149964	3.688506	-0.463066
C	-5.122467	2.764464	-0.516909
C	-4.843585	1.292867	-0.635810
C	-4.137029	-1.402062	-0.669671
C	-3.663600	-2.820602	-0.593159
C	-2.351668	-3.168753	-0.549982
C	-1.295224	-2.156042	-0.544783
C	-1.938559	-4.558864	-0.510615
C	-0.635326	-4.916371	-0.483967
C	0.434383	-3.933389	-0.483640
C	0.061156	-2.536563	-0.508977
C	1.773508	-4.307735	-0.455248
C	-2.113152	5.798060	-0.410174
C	2.159211	-5.758666	-0.421502
C	5.816632	-0.281944	-0.404488
C	5.477706	1.049011	-0.404818
C	7.310847	-0.385812	-0.378252
C	6.734493	1.856351	-0.378431
O	8.017979	-1.377837	-0.367626
O	6.878782	3.066444	-0.367704
C	-5.598232	0.441535	0.445023
C	-5.207413	-1.056564	0.426975
C	-7.112659	0.451962	0.232807
C	-6.522897	-1.802045	0.200286
O	-7.848966	1.418197	0.177454
O	-6.690926	-3.003651	0.110613
C	2.308180	-6.420565	0.815259
C	2.674290	-7.771210	0.825025
C	2.896972	-8.485663	-0.354443
C	2.748600	-7.809199	-1.569112
C	2.383446	-6.460734	-1.625744
C	-2.278875	6.448119	0.831654
C	-2.621735	7.803809	0.850288
C	-2.802205	8.537477	-0.325782
C	-2.637749	7.873580	-1.544161
C	-2.296415	6.518293	-1.609367
C	2.236215	-5.776432	-2.966630
C	2.079877	-5.694505	2.122522
C	3.265648	-9.950737	-0.322917
C	-2.099616	5.699658	2.133868
C	-2.136758	5.847221	-2.955555
C	-3.139597	10.009679	-0.279572
N	7.786678	0.930122	-0.371761
C	13.828013	2.329488	4.045028
C	13.275286	2.233952	2.619381
C	11.786192	1.867884	2.572706
C	11.224758	1.766216	1.148806
C	9.737255	1.394653	1.112408
C	9.197960	1.290333	-0.319762
N	-7.545105	-0.864261	0.089758
C	-14.147967	-2.837377	3.322928
C	-13.392677	-2.543196	2.022958
C	-11.922794	-2.167607	2.250703
C	-11.158138	-1.878653	0.952614
C	-9.690633	-1.500702	1.189372
C	-8.945896	-1.228848	-0.123027
H	4.452824	-4.703144	-0.416476
H	6.218454	-2.982252	-0.399060
H	4.528398	3.598504	-0.401902
H	2.780919	5.357204	-0.403501
H	0.413726	6.004935	-0.416285
H	-4.415700	4.738610	-0.393651
H	-6.166566	3.058085	-0.483013
H	-5.242529	0.952321	-1.610742
H	-4.661536	-1.274454	-1.637159
H	-4.430883	-3.588574	-0.583226
H	-2.716918	-5.317821	-0.509222
H	-0.359623	-5.965163	-0.460894
H	-5.392493	0.892135	1.421248
H	-4.802705	-1.372900	1.393548
H	2.789949	-8.275854	1.782320
H	2.923445	-8.344344	-2.500759
H	-2.751477	8.298489	1.811135

H	-2.779493	8.423515	-2.472653
H	2.460537	-6.468640	-3.783899
H	1.218695	-5.396396	-3.116252
H	2.911387	-4.917338	-3.057937
H	1.050004	-5.327393	2.206013
H	2.271012	-6.355146	2.973641
H	2.735946	-4.821514	2.219318
H	3.688998	-10.236677	0.645483
H	2.387097	-10.586592	-0.496468
H	3.998953	-10.196625	-1.099075
H	-1.096048	5.266025	2.215944
H	-2.250326	6.364652	2.989701
H	-2.810788	4.869632	2.223102
H	-2.281342	6.565384	-3.768422
H	-1.141286	5.402669	-3.070426
H	-2.862485	5.036164	-3.090245
H	-3.765061	10.251091	0.586758
H	-2.232084	10.623862	-0.204649
H	-3.672982	10.327214	-1.181741
H	14.892723	2.589415	4.045192
H	13.298108	3.094977	4.625264
H	13.717354	1.376593	4.577221
H	13.849420	1.486714	2.053255
H	13.429482	3.191508	2.101837
H	11.210837	2.616245	3.137569
H	11.631803	0.910510	3.092182
H	11.801074	1.018419	0.584161
H	11.375247	2.724419	0.629728
H	9.150946	2.145851	1.657532
H	9.580841	0.433533	1.619193
H	9.744652	0.525675	-0.879899
H	9.302309	2.246984	-0.840256
H	-15.193309	-3.101542	3.126880
H	-13.689756	-3.672378	3.867104
H	-14.143930	-1.966520	3.990002
H	-13.895164	-1.727877	1.483398
H	-13.443885	-3.421206	1.363376
H	-11.420470	-2.981505	2.794106
H	-11.870870	-1.286133	2.906867
H	-11.660549	-1.066051	0.407597
H	-11.206359	-2.762167	0.299121
H	-9.179152	-2.310113	1.725785
H	-9.633804	-0.604998	1.821552
H	-9.408199	-0.399270	-0.664699
H	-8.950007	-2.118554	-0.758922

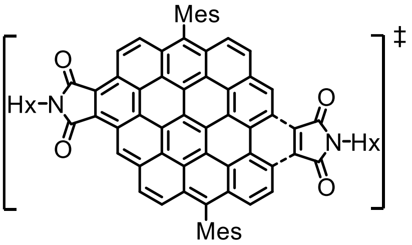

TS4

Charge = 0; Multiplicity = 1

	x	y	z
C	-3.484567	0.868402	-0.487201
C	-3.078139	-0.505129	-0.494468
C	-1.697950	-0.837525	-0.581743
C	-0.718763	0.199154	-0.672951
C	-1.122490	1.566051	-0.659541
C	-2.501639	1.903353	-0.563818
C	0.654552	-0.134568	-0.772092
C	1.065518	-1.500915	-0.753984
C	2.447457	-1.831494	-0.813846
C	3.419280	-0.795776	-0.948689
C	3.011547	0.556565	-1.013215
C	1.630630	0.898122	-0.877756
C	-2.899900	3.276279	-0.539917
C	-4.293001	3.576085	-0.450727
C	-5.243294	2.590204	-0.384613
C	-4.867352	1.220086	-0.397339
C	-4.046799	-1.562715	-0.408815
C	-3.625922	-2.904079	-0.405296
C	-2.274543	-3.236034	-0.481325
C	-1.288406	-2.199163	-0.574158
C	-1.829903	-4.600736	-0.463299
C	-0.507592	-4.918188	-0.523409
C	0.509921	-3.904317	-0.615815
C	0.094648	-2.533932	-0.649800
C	1.884846	-4.230381	-0.667277
C	2.851003	-3.205421	-0.756711
C	4.264186	-3.498777	-0.808678

C	5.201072	-2.523443	-0.881658	C	-9.567920	-1.668658	1.414125
C	4.843742	-1.104425	-0.858617	C	-9.108212	-1.542265	-0.043871
C	3.978690	1.610999	-1.081179	N	7.331813	0.991549	0.481739
C	3.567730	2.955363	-1.041114	C	14.046877	2.588591	3.693549
C	2.226382	3.295942	-0.904809	C	13.213118	2.557655	2.408578
C	1.229445	2.259878	-0.840319	C	11.781850	2.051481	2.629274
C	1.787657	4.664471	-0.860398	C	10.943201	2.008785	1.345374
C	0.471203	4.986780	-0.763564	C	9.512131	1.508334	1.576208
C	-0.556986	3.974890	-0.697372	C	8.698919	1.453667	0.276497
C	-0.147252	2.597020	-0.733212	H	-4.598026	4.616769	-0.435438
C	-1.916302	4.307525	-0.600641	H	-6.297103	2.835226	-0.318013
C	2.314652	-5.669037	-0.627153	H	-4.376270	-3.684541	-0.337093
C	-2.342906	5.746008	-0.549204	H	-2.580203	-5.384079	-0.394411
C	-5.796577	0.152229	-0.319203	H	-0.196456	-5.956952	-0.500824
C	-5.409618	-1.169115	-0.323840	H	4.569378	-4.539886	-0.801690
C	-7.289685	0.202382	-0.214938	H	6.255974	-2.773820	-0.907176
C	-6.633763	-2.019345	-0.222993	H	5.474027	-0.505855	-1.519560
O	-8.031171	1.168225	-0.177633	H	4.961561	1.400572	-1.484154
O	-6.733563	-3.233655	-0.193221	H	4.316788	3.738471	-1.108579
C	5.443646	-0.372010	0.731685	H	2.541640	5.446045	-0.906426
C	5.067311	1.000111	0.933730	H	0.166781	6.027313	-0.732530
C	6.961589	-0.340786	0.553143	H	5.080374	-1.128033	1.423781
C	6.247602	1.853643	0.798722	H	4.208820	1.337496	1.495689
O	7.720013	-1.288514	0.441591	H	-2.924211	8.249096	1.683374
O	6.362246	3.065252	0.903983	H	-3.252465	8.315224	-2.589055
C	-2.449519	6.404294	0.694476	H	3.201990	-8.104863	1.579859
C	-2.847113	7.745284	0.721794	H	2.961920	-8.294776	-2.694812
C	-3.145920	8.452504	-0.445593	H	-2.793370	6.454647	-3.895143
C	-3.030309	7.781943	-1.666594	H	-1.509776	5.401574	-3.278757
C	-2.636293	6.442124	-1.741551	H	-3.191114	4.894236	-3.159049
C	2.587430	-6.291861	0.609907	H	-1.098411	5.338517	2.019547
C	2.989360	-7.631060	0.623323	H	-2.294895	6.347016	2.847638
C	3.126949	-8.374239	-0.552744	H	-2.772162	4.803079	2.124049
C	2.855300	-7.737307	-1.766222	H	-4.701331	9.957493	-0.369001
C	2.450803	-6.399247	-1.826686	H	-3.232920	10.395503	0.508773
C	-2.527065	5.764059	-3.089289	H	-3.263313	10.456359	-1.262588
C	-2.137658	5.686911	1.989354	H	1.443080	-5.138958	2.049785
C	-3.605237	9.890898	-0.388933	H	2.686697	-6.178348	2.764308
C	2.457003	-5.532444	1.911507	H	3.138014	-4.673591	1.947701
C	2.173314	-5.758370	-3.168596	H	2.330489	-6.473700	-3.981538
C	3.533415	-9.828900	-0.509157	H	1.141973	-5.392519	-3.236913
N	-7.715385	-1.130312	-0.169043	H	2.827115	-4.896600	-3.347621
C	-13.465964	-2.778501	4.542957	H	4.244896	-10.022619	0.300996
C	-12.987669	-2.659845	3.092290	H	2.665105	-10.479689	-0.339337
C	-11.520452	-2.227931	2.973991	H	3.996880	-10.143993	-1.449868
C	-11.034587	-2.102818	1.524206	H	-14.516255	-3.087316	4.594366

H	-12.873853	-3.517712	5.096484
H	-13.374026	-1.820671	5.069839
H	-13.621284	-1.939445	2.555597
H	-13.123953	-3.623790	2.581684
H	-10.885387	-2.949492	3.508926
H	-11.383662	-1.264211	3.486611
H	-11.669784	-1.380714	0.990150
H	-11.169480	-3.066807	1.011629
H	-8.923687	-2.394931	1.926604
H	-9.426178	-0.701949	1.914595
H	-9.710878	-0.797730	-0.572607
H	-9.204261	-2.501645	-0.561003
H	15.061505	2.957082	3.503758
H	13.590419	3.242657	4.446742
H	14.132960	1.587745	4.134272
H	13.711459	1.920536	1.664018
H	13.177217	3.565461	1.970745
H	11.280565	2.692367	3.369567
H	11.817169	1.045301	3.072498
H	11.442218	1.361956	0.608475
H	10.910850	3.013613	0.898763
H	8.995622	2.167406	2.285764
H	9.533955	0.504941	2.020216
H	9.170518	0.773740	-0.439917
H	8.631298	2.448757	-0.172868

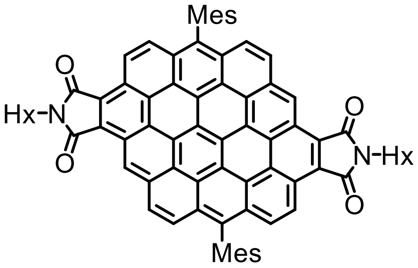

4a

Charge = 0; Multiplicity = 1

	x	y	z
C	3.446447	-0.896819	-0.397503
C	3.064122	0.483665	-0.396906
C	1.686182	0.840490	-0.398538
C	0.684385	-0.178372	-0.399198
C	1.066955	-1.554881	-0.399588
C	2.442465	-1.915124	-0.399448
C	-0.684470	0.178415	-0.398894
C	-1.067040	1.554924	-0.399200
C	-2.442549	1.915166	-0.398411
C	-3.446531	0.896862	-0.395960
C	-3.064206	-0.483622	-0.395470
C	-1.686267	-0.840446	-0.397692
C	2.818760	-3.295481	-0.400635
C	4.211496	-3.618540	-0.400179
C	5.178771	-2.649793	-0.397434
C	4.825330	-1.271555	-0.395530
C	4.054862	1.522992	-0.394213
C	3.658434	2.874351	-0.395225
C	2.314437	3.229150	-0.397938
C	1.303540	2.209860	-0.398845
C	1.897737	4.605454	-0.399416
C	0.583783	4.949684	-0.401035
C	-0.461054	3.953936	-0.400740
C	-0.073618	2.569608	-0.399775
C	-1.819568	4.309104	-0.401095
C	-2.818843	3.295522	-0.399412
C	-4.211580	3.618583	-0.398164
C	-5.178855	2.649837	-0.395024
C	-4.825413	1.271599	-0.393361
C	-4.054944	-1.522949	-0.392285
C	-3.658516	-2.874308	-0.393342
C	-2.314520	-3.229107	-0.396587
C	-1.303625	-2.209816	-0.398056
C	-1.897821	-4.605412	-0.398036
C	-0.583868	-4.949640	-0.400226
C	0.460968	-3.953890	-0.400661
C	0.073533	-2.569563	-0.399603
C	1.819477	-4.309063	-0.401778
C	-2.223832	5.755082	-0.402955
C	2.223747	-5.755039	-0.404045
C	5.775395	-0.221890	-0.389829
C	5.411844	1.107350	-0.388927
C	7.270611	-0.298024	-0.381511
C	6.654546	1.935795	-0.379928
O	7.996460	-1.276533	-0.376743

O	6.778104	3.148330	-0.373728
C	-5.775475	0.221933	-0.387265
C	-5.411923	-1.107307	-0.386476
C	-7.270688	0.298067	-0.378388
C	-6.654621	-1.935752	-0.377005
O	-7.996535	1.276576	-0.373428
O	-6.778178	-3.148286	-0.370770
C	2.413371	-6.437907	0.816452
C	2.788385	-7.785154	0.790366
C	2.985130	-8.475620	-0.408733
C	2.787812	-7.781208	-1.605388
C	2.412804	-6.433828	-1.626845
C	-2.410739	6.438323	0.817718
C	-2.785818	7.785588	0.792034
C	-2.985215	8.475663	-0.406816
C	-2.790566	7.780856	-1.603713
C	-2.415626	6.433492	-1.625573
C	2.213203	-5.729685	-2.950677
C	2.214187	-5.738150	2.142669
C	3.423019	-9.921778	-0.411105
C	-2.219228	5.728817	-2.949599
C	-2.208533	5.739045	2.143733
C	-3.423039	9.921844	-0.408813
N	7.721465	1.027117	-0.383710
C	13.762385	2.548359	3.990779
C	13.204460	2.438828	2.568145
C	11.721358	2.048061	2.530394
C	11.154394	1.931153	1.109826
C	9.673298	1.533723	1.083923
C	9.126918	1.412748	-0.344270
N	-7.721542	-1.027075	-0.380408
C	-13.761542	-2.548744	3.995247
C	-13.203874	-2.439123	2.572519
C	-11.720792	-2.048308	2.534518
C	-11.154105	-1.931294	1.113848
C	-9.673021	-1.533839	1.087663
C	-9.126984	-1.412709	-0.340647
H	4.499505	-4.664171	-0.401547
H	6.230672	-2.911573	-0.396496
H	4.426195	3.640658	-0.393703
H	2.667273	5.373006	-0.399248
H	0.294570	5.994949	-0.402279
H	-4.499585	4.664216	-0.399239
H	-6.230755	2.911617	-0.393546
H	-4.426276	-3.640615	-0.391423
H	-2.667357	-5.372963	-0.397335
H	-0.294649	-5.994904	-0.401354
H	2.929613	-8.307967	1.734440
H	2.928555	-8.300889	-2.551249
H	-2.924968	8.308695	1.736251
H	-2.933385	8.300246	-2.549427
H	2.406540	-6.409167	-3.786190
H	1.190659	-5.348416	-3.056330
H	2.884398	-4.868958	-3.055077
H	2.884140	-4.876704	2.248975
H	1.191146	-5.358817	2.250613
H	2.409467	-6.419933	2.975859
H	3.052122	-10.454895	0.470819
H	3.064040	-10.448328	-1.301803
H	4.518137	-10.005137	-0.403916
H	-2.411496	6.408831	-3.784926
H	-1.197906	5.344611	-3.056221
H	-2.892998	4.869996	-3.053363
H	-1.184668	5.361527	2.250327
H	-2.403823	6.420585	2.977119
H	-2.876820	4.876447	2.251036
H	-4.518173	10.005255	-0.406423
H	-3.055981	10.453519	0.475559
H	-3.060127	10.449804	-1.297105
H	14.822808	2.825089	3.984867
H	13.223192	3.307452	4.570839
H	13.669302	1.595735	4.526768
H	13.787990	1.699091	2.001792
H	13.340232	3.396904	2.046491
H	11.136505	2.789101	3.095151
H	11.585398	1.090411	3.054416
H	11.740329	1.191021	0.545031
H	11.285429	2.889654	0.586166
H	9.077370	2.277413	1.628902
H	9.536763	0.572678	1.596503
H	9.682618	0.654833	-0.904536
H	9.210422	2.368417	-0.870149
H	-14.821955	-2.825515	3.989510
H	-13.222216	-3.307844	4.575174
H	-13.668401	-1.596142	4.531265
H	-13.787529	-1.699374	2.006308
H	-13.339711	-3.397176	2.050840
H	-11.135810	-2.789364	3.099118
H	-11.584763	-1.090685	3.058572
H	-11.740166	-1.191134	0.549219
H	-11.285230	-2.889762	0.590149
H	-9.076958	-2.277577	1.632428

H	-9.536384	-0.572845	1.600311	H	-9.210628	-2.368320	-0.866610
H	-9.682820	-0.654729	-0.900689				

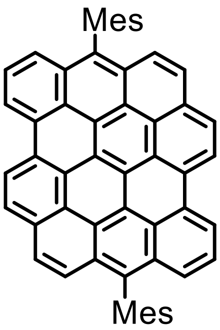

DBOV-Mes 1

Charge = 0; Multiplicity = 1

	x	y	z				
				H	0.424890	-4.341739	0.002403
C	-2.132934	-3.689244	0.001680	C	2.214552	-3.169569	0.001734
C	-1.535630	-2.430713	0.001107	C	2.767541	-1.852389	0.000886
C	-2.379726	-1.272200	0.000601	C	2.448398	0.578854	-0.000634
C	-3.809177	-1.426614	0.000722	C	3.883321	0.756764	-0.000848
C	-4.353502	-2.738398	0.001550	C	4.392417	2.111398	-0.001952
C	-3.527861	-3.840872	0.001958	H	5.468138	2.250797	-0.002493
C	-1.814317	0.034701	-0.000042	C	4.736573	-0.345025	-0.000006
C	-4.656924	-0.279460	0.000025	C	3.077823	-4.280883	0.002636
C	-4.093506	1.002978	-0.000557	H	2.653039	-5.281593	0.003227
C	-2.661805	1.165289	-0.000526	H	1.697074	5.128845	-0.002511
C	-2.099368	2.483629	-0.001047	H	-3.025428	5.783217	-0.002081
C	-2.944383	3.624933	-0.001449	C	6.226131	-0.161875	0.000268
C	-4.363734	3.433872	-0.001547	C	6.930471	-0.087910	-1.220874
C	-4.912529	2.188665	-0.001228	C	6.928698	-0.079555	1.221191
H	-1.522034	-4.584822	0.001947	C	8.320145	0.068336	-1.197348
H	-5.431089	-2.859004	0.001821	C	8.318942	0.076826	1.198135
H	-3.954840	-4.840152	0.002507	C	9.034671	0.155507	0.000812
H	-5.003512	4.313327	-0.001975	H	8.858067	0.121195	-2.142262
H	-5.990320	2.067812	-0.001485	H	8.855751	0.136458	2.143186
C	-0.072153	-2.257046	0.001034	C	6.202252	-0.157832	2.545547
C	-0.380292	0.205115	-0.000203	H	5.487476	0.665469	2.662326
C	-0.684196	2.655265	-0.001190	H	5.630227	-1.088449	2.637231
C	0.474052	-0.918909	0.000289	H	6.907944	-0.111332	3.380717
C	0.174544	1.517947	-0.000839	C	6.205107	-0.175227	-2.545243
C	-2.366870	4.917917	-0.001844	H	5.637258	-1.108800	-2.633074
C	-1.002160	5.087933	-0.001973	H	5.486728	0.644331	-2.665957
H	-0.573435	6.087023	-0.002305	H	6.911036	-0.129384	-3.380247
C	-0.127480	3.970739	-0.001720	C	10.532934	0.353106	-0.000244
C	1.280248	4.123852	-0.002038	H	11.003786	-0.152120	-0.850674
C	1.588318	1.700028	-0.001102	H	10.795100	1.417461	-0.070993
C	1.893443	-0.732865	0.000154	H	10.988787	-0.032815	0.917728
C	0.800504	-3.323997	0.001729	C	4.189169	-1.673397	0.000967

C	4.458018	-4.101591	0.002873
C	2.132744	3.035322	-0.001806
C	5.009602	-2.823229	0.002130
H	5.114797	-4.967474	0.003693
C	-6.147862	-0.453905	-0.000212
C	-6.850668	-0.536315	-1.221020
C	-6.852005	-0.530033	1.220987
C	-8.240890	-0.693137	-1.197828
C	-8.241627	-0.686662	1.197613
C	-8.956402	-0.773084	-0.000480
H	-8.777785	-0.752550	-2.142834
H	-8.779265	-0.740839	2.142613
C	-10.454481	-0.972051	0.000813
H	-10.910123	-0.591599	-0.919519
H	-10.715721	-2.036221	0.077418
H	-10.926283	-0.462532	0.848171
C	-6.127217	-0.437712	2.545348
H	-5.365963	-1.220436	2.643520
H	-5.609160	0.522403	2.655164
H	-6.826660	-0.540387	3.380728
C	-6.125350	-0.450657	-2.545545
H	-5.605060	0.507830	-2.658856
H	-5.365889	-1.235511	-2.640627
H	-6.824814	-0.554906	-3.380710
H	6.087227	-2.700274	0.002506
C	3.565423	3.185622	-0.002313
H	3.974109	4.193330	-0.003071

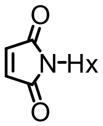

***N*-*n*-hexylmaleimide (2a)**

Charge = 0; Multiplicity = 1

	x	y	z
C	2.492063	1.157633	-0.007228
C	3.784350	0.722233	-0.642005
C	3.820299	-0.613259	-0.664697
C	2.554250	-1.139265	-0.045938
H	4.515465	1.437872	-0.995912
H	4.588545	-1.276202	-1.041811
O	2.222401	-2.296440	0.113766
O	2.097129	2.288953	0.190648
N	1.811686	-0.015849	0.328849
C	0.493810	-0.060691	0.952549
H	0.448603	-0.989698	1.528709
H	0.437458	0.782528	1.647976
C	-0.654154	0.002553	-0.062020
H	-0.553730	-0.835457	-0.764300
H	-0.558326	0.926526	-0.646878
C	-2.029383	-0.046061	0.614897
H	-2.109963	-0.966738	1.211580
H	-2.114948	0.788317	1.326654
C	-3.195502	0.015728	-0.379992
H	-3.114631	0.935898	-0.977396
H	-3.109563	-0.817924	-1.092607
C	-4.574246	-0.032573	0.290794
H	-4.659846	0.800934	1.002417
H	-4.653906	-0.951773	0.888462
C	-5.733687	0.027955	-0.708771
H	-6.703403	-0.008957	-0.199511
H	-5.701810	0.952852	-1.297839
H	-5.694784	-0.812853	-1.412327

H_2

Charge = 0; Multiplicity = 1

	x	y	z
H	0.000000	0.000000	0.371410
H	0.000000	0.000000	-0.371410

7.3 Energy and thermodynamic quantities for the species involved in the Diels-Alder cycloaddition of DBOV-Mes 1 with N-*n*-hexylmaleimide (2a). The properties were obtained using diphenyl ether as solvent with the polarizable continuum model (PCM) at the B3LYP/6-31G(d)//B3LYP/6-31G(d) level of theory with Gaussian 09 program (revision B.01).

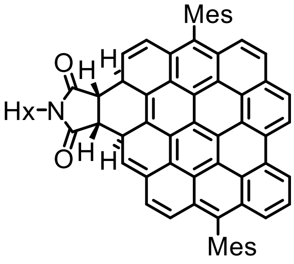

Int1

E(RB3LYP) = –2751.20497604 a.u.

Zero-point correction =	0.983979 (Hartree/Particle)
Thermal correction to Energy =	1.040551
Thermal correction to Enthalpy =	1.041495
Thermal correction to Gibbs Free Energy =	0.888447
Sum of electronic and zero-point Energies =	-2750.220997
Sum of electronic and thermal Energies =	-2750.164425
Sum of electronic and thermal Enthalpies =	-2750.163481
Sum of electronic and thermal Free Energies =	-2750.316529

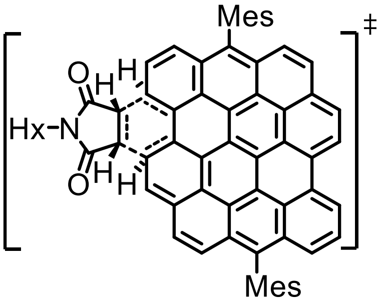

TS1

E(RB3LYP) = –2751.16053390 a.u.

Zero-point correction =	0.979848 (Hartree/Particle)
Thermal correction to Energy =	1.036941
Thermal correction to Enthalpy =	1.037885
Thermal correction to Gibbs Free Energy =	0.884082
Sum of electronic and zero-point Energies =	-2750.180686
Sum of electronic and thermal Energies =	-2750.123593
Sum of electronic and thermal Enthalpies =	-2750.122649
Sum of electronic and thermal Free Energies =	-2750.276452

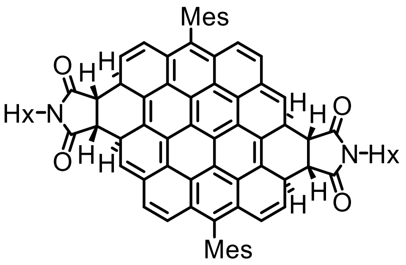

Int2

E(RB3LYP) = –3346.50339910 a.u.

Zero-point correction =	1.227718 (Hartree/Particle)
Thermal correction to Energy =	1.296591
Thermal correction to Enthalpy =	1.297535
Thermal correction to Gibbs Free Energy =	1.113923
Sum of electronic and zero-point Energies =	-3345.275681
Sum of electronic and thermal Energies =	-3345.206809
Sum of electronic and thermal Enthalpies =	-3345.205864
Sum of electronic and thermal Free Energies =	-3345.389476

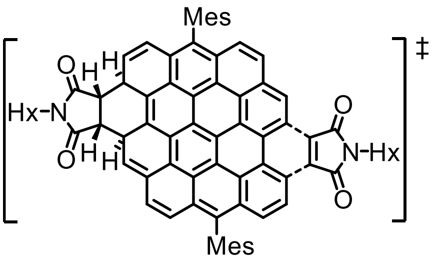

TS2

E(RB3LYP) = –3346.47047062 a.u.

Zero-point correction =	1.224690 (Hartree/Particle)
Thermal correction to Energy =	1.295513
Thermal correction to Enthalpy =	1.296457
Thermal correction to Gibbs Free Energy =	1.108916
Sum of electronic and zero-point Energies =	-3345.245781
Sum of electronic and thermal Energies =	-3345.174958
Sum of electronic and thermal Enthalpies =	-3345.174014
Sum of electronic and thermal Free Energies =	-3345.361555

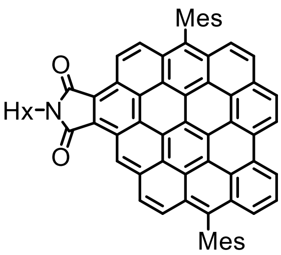

3a

E(RB3LYP)=-2748.86300374 a.u.

Zero-point correction= 0.937389 (Hartree/Particle)
Thermal correction to Energy= 0.993409
Thermal correction to Enthalpy= 0.994353
Thermal correction to Gibbs Free Energy= 0.843596
Sum of electronic and zero-point Energies= -2747.925615
Sum of electronic and thermal Energies= -2747.869595
Sum of electronic and thermal Enthalpies= -2747.868651
Sum of electronic and thermal Free Energies= -2748.019407

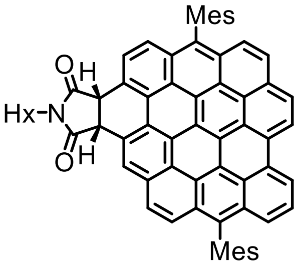

3a'

E(RB3LYP)=-2750.04629776 a.u.

Zero-point correction= 0.961170 (Hartree/Particle)
Thermal correction to Energy= 1.017312
Thermal correction to Enthalpy= 1.018257
Thermal correction to Gibbs Free Energy= 0.865560
Sum of electronic and zero-point Energies= -2749.085128
Sum of electronic and thermal Energies= -2749.028985
Sum of electronic and thermal Enthalpies= -2749.028041
Sum of electronic and thermal Free Energies= -2749.180738

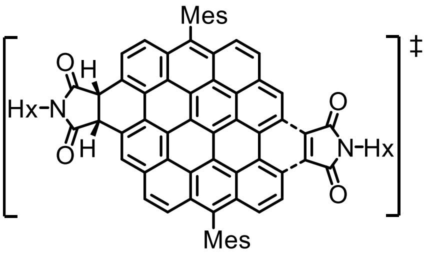

TS3

E(RB3LYP)=-3345.32351688 a.u.

Zero-point correction= 1.201812 (Hartree/Particle)
Thermal correction to Energy= 1.272434
Thermal correction to Enthalpy= 1.273378
Thermal correction to Gibbs Free Energy= 1.086099
Sum of electronic and zero-point Energies= -3344.121705

Sum of electronic and thermal Energies= -3344.051083
Sum of electronic and thermal Enthalpies= -3344.050139
Sum of electronic and thermal Free Energies= -3344.237418

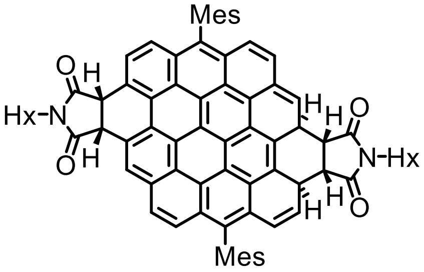

Int3

E(RB3LYP)=-3345.36771297 a.u.

Zero-point correction= 1.206214 (Hartree/Particle)
Thermal correction to Energy= 1.276180
Thermal correction to Enthalpy= 1.277124
Thermal correction to Gibbs Free Energy= 1.092074
Sum of electronic and zero-point Energies= -3344.161499
Sum of electronic and thermal Energies= -3344.091533
Sum of electronic and thermal Enthalpies= -3344.090589
Sum of electronic and thermal Free Energies= -3344.275639

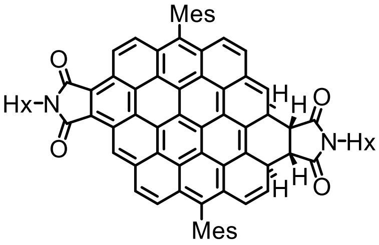

Int4

E(RB3LYP) = –3344.17713999 a.u.

Zero-point correction = 1.182150 (Hartree/Particle)
Thermal correction to Energy = 1.252068
Thermal correction to Enthalpy = 1.253012
Thermal correction to Gibbs Free Energy = 1.067995
Sum of electronic and zero-point Energies = -3342.994990
Sum of electronic and thermal Energies = -3342.925072
Sum of electronic and thermal Enthalpies = -3342.924128
Sum of electronic and thermal Free Energies = -3343.109145

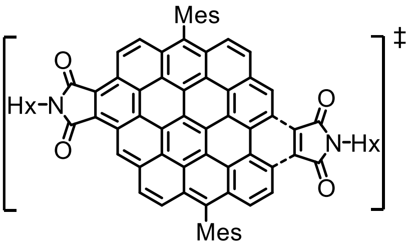

TS4

E(RB3LYP) = –3344.13504037 a.u.

Zero-point correction =	1.178055 (Hartree/Particle)
Thermal correction to Energy =	1.248547
Thermal correction to Enthalpy =	1.249491
Thermal correction to Gibbs Free Energy =	1.063092
Sum of electronic and zero-point Energies =	-3342.956985
Sum of electronic and thermal Energies =	-3342.886494
Sum of electronic and thermal Enthalpies =	-3342.885550
Sum of electronic and thermal Free Energies =	-3343.071948

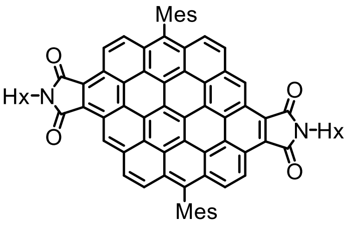

4a

E(RB3LYP) = –3341.83903751 a.u.

Zero-point correction =	1.135590 (Hartree/Particle)
Thermal correction to Energy =	1.204985
Thermal correction to Enthalpy =	1.205930
Thermal correction to Gibbs Free Energy =	1.022072
Sum of electronic and zero-point Energies =	-3340.703447
Sum of electronic and thermal Energies =	-3340.634052
Sum of electronic and thermal Enthalpies =	-3340.633108
Sum of electronic and thermal Free Energies =	-3340.816966

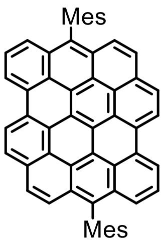

1

E(RB3LYP) = -2155.87733092 a.u.

Zero-point correction =	0.738179 (Hartree/Particle)

Thermal correction to Energy = 0.781100
Thermal correction to Enthalpy = 0.782044
Thermal correction to Gibbs Free Energy = 0.660937
Sum of electronic and zero-point Energies = -2155.139152
Sum of electronic and thermal Energies = -2155.096231
Sum of electronic and thermal Enthalpies = -2155.095287
Sum of electronic and thermal Free Energies = -2155.216394

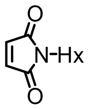

2a

E(RB3LYP) = –595.32138221 a.u.

Zero-point correction = 0.239708 (Hartree/Particle)
Thermal correction to Energy = 0.253484
Thermal correction to Enthalpy = 0.254428
Thermal correction to Gibbs Free Energy = 0.196690
Sum of electronic and zero-point Energies = -595.081674
Sum of electronic and thermal Energies = -595.067899
Sum of electronic and thermal Enthalpies = -595.066954
Sum of electronic and thermal Free Energies = -595.124692

H_2

E(RB3LYP) -1.17553752 a.u.

Zero-point correction = 0.010142 (Hartree/Particle)
Thermal correction to Energy = 0.012502
Thermal correction to Enthalpy = 0.013446
Thermal correction to Gibbs Free Energy = -0.001346
Sum of electronic and zero-point Energies = -1.165396
Sum of electronic and thermal Energies = -1.163035
Sum of electronic and thermal Enthalpies = -1.162091

7.4 Energy, thermodynamic quantities and cartesian coordinates for the species involved in the Diels-Alder cycloaddition of DBOV-Mes 1 with N-*n*-hexylmaleimide (2a). The properties were obtained using diphenyl ether as solvent with the polarizable continuum model (PCM) at the M062X/6-31G(d)//B3LYP/6-31G(d) level of theory with Gaussian 09 program (revision B.01).

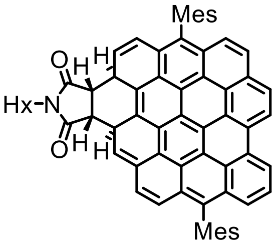

Int1

E(RM062X) = –2750.09735130 a.u.

Zero-point correction =	0.991796 (Hartree/Particle)
Thermal correction to Energy =	1.046954
Thermal correction to Enthalpy =	1.047898
Thermal correction to Gibbs Free Energy =	0.904682
Sum of electronic and zero-point Energies =	-2749.105555
Sum of electronic and thermal Energies =	-2749.050398
Sum of electronic and thermal Enthalpies =	-2749.049454
Sum of electronic and thermal Free Energies =	-2749.192670

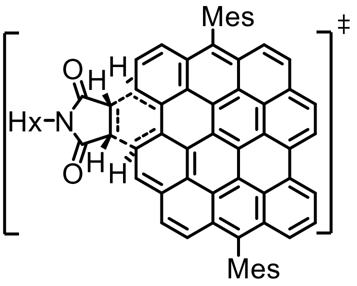

TS1

E(RM062X) = –2750.03669833 a.u.

Zero-point correction =	0.984984 (Hartree/Particle)
Thermal correction to Energy =	1.041174
Thermal correction to Enthalpy =	1.042119
Thermal correction to Gibbs Free Energy =	0.895452
Sum of electronic and zero-point Energies =	-2749.051715
Sum of electronic and thermal Energies =	-2748.995524
Sum of electronic and thermal Enthalpies =	-2748.994580
Sum of electronic and thermal Free Energies =	-2749.141246

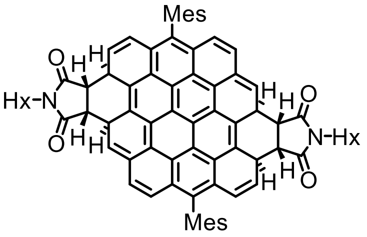

Int2

E(RM062X) = -3345.14617773 a.u.

Zero-point correction =	1.236808 (Hartree/Particle)
Thermal correction to Energy =	1.305661
Thermal correction to Enthalpy =	1.306605
Thermal correction to Gibbs Free Energy =	1.132647
Sum of electronic and zero-point Energies =	-3343.909370
Sum of electronic and thermal Energies =	-3343.840517
Sum of electronic and thermal Enthalpies =	-3343.839573

Sum of electronic and thermal Free Energies = -3344.013531

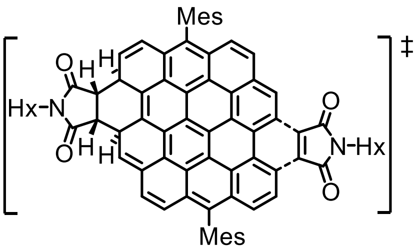

TS2

E(RM062X) = –3345.10113527 a.u.

Zero-point correction =	1.232087 (Hartree/Particle)
Thermal correction to Energy =	1.301834
Thermal correction to Enthalpy =	1.302779
Thermal correction to Gibbs Free Energy =	1.125230
Sum of electronic and zero-point Energies =	-3343.869048
Sum of electronic and thermal Energies =	-3343.799301
Sum of electronic and thermal Enthalpies =	-3343.798357
Sum of electronic and thermal Free Energies =	-3343.975906

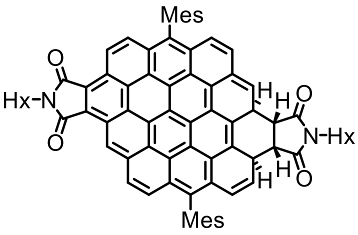

Int4

E(RM062X) = –3342.82504826 a.u.

Zero-point correction =	1.190542 (Hartree/Particle)
Thermal correction to Energy =	1.258900
Thermal correction to Enthalpy =	1.259844
Thermal correction to Gibbs Free Energy =	1.087065
Sum of electronic and zero-point Energies =	-3341.634506
Sum of electronic and thermal Energies =	-3341.566149
Sum of electronic and thermal Enthalpies =	-3341.565204
Sum of electronic and thermal Free Energies =	-3341.737983

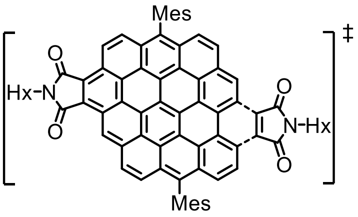

TS4

E(RM062X) = –3342.76827333 a.u.

Zero-point correction =	1.185196 (Hartree/Particle)
Thermal correction to Energy =	1.254447
Thermal correction to Enthalpy =	1.255391
Thermal correction to Gibbs Free Energy =	1.079392
Sum of electronic and zero-point Energies =	-3341.583077
Sum of electronic and thermal Energies =	-3341.513826
Sum of electronic and thermal Enthalpies =	-3341.512882
Sum of electronic and thermal Free Energies =	-3341.688882

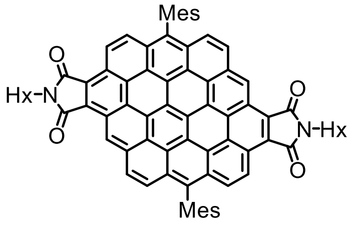

4a

E(RM062X) = –3340.48796742 a.u.

Zero-point correction =	1.142757 (Hartree/Particle)
Thermal correction to Energy =	1.210763
Thermal correction to Enthalpy =	1.211707
Thermal correction to Gibbs Free Energy =	1.039313
Sum of electronic and zero-point Energies =	-3339.345210
Sum of electronic and thermal Energies =	-3339.277205
Sum of electronic and thermal Enthalpies =	-3339.276261
Sum of electronic and thermal Free Energies =	-3339.448654

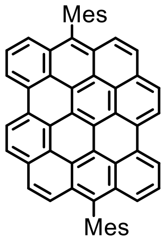

1

E(RM062X) = –2155.00914740 a.u.

Zero-point correction =	0.742277 (Hartree/Particle)
Thermal correction to Energy =	0.784756
Thermal correction to Enthalpy =	0.785700
Thermal correction to Gibbs Free Energy =	0.668867
Sum of electronic and zero-point Energies =	-2154.266870
Sum of electronic and thermal Energies =	-2154.224392
Sum of electronic and thermal Enthalpies =	-2154.223448
Sum of electronic and thermal Free Energies =	-2154.340280

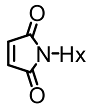

2a

E(RM062X) = –595.05208303 a.u.

Zero-point correction =	0.241440 (Hartree/Particle)
Thermal correction to Energy =	0.254895
Thermal correction to Enthalpy =	0.255839
Thermal correction to Gibbs Free Energy =	0.200300
Sum of electronic and zero-point Energies =	-594.810643
Sum of electronic and thermal Energies =	-594.797189
Sum of electronic and thermal Enthalpies =	-594.796244
Sum of electronic and thermal Free Energies =	-594.851783

H_2

E(RM062X) = –1.16359349 a.u.

Zero-point correction =	0.010085 (Hartree/Particle)
Thermal correction to Energy =	0.012446
Thermal correction to Enthalpy =	0.013390
Thermal correction to Gibbs Free Energy =	-0.001402
Sum of electronic and zero-point Energies =	-1.153508
Sum of electronic and thermal Energies =	-1.151148
Sum of electronic and thermal Enthalpies =	-1.150203
Sum of electronic and thermal Free Energies =	-1.164995

7.5 Energy, thermodynamic quantities and cartesian coordinates for the species involved in the Diels-Alder cycloaddition of DBOV-Mes 1 with N-*n*-hexylmaleimide (2a). The properties were obtained using diphenyl ether as solvent with the polarizable continuum model (PCM) at the wB97XD/6-31G(d)//B3LYP/6-31G(d) level of theory with Gaussian 09 program (revision B.01).

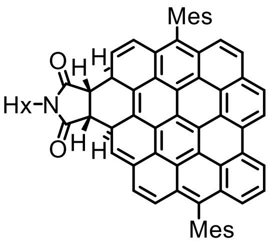

Int1

E(RwB97XD) = –2750.36071644 a.u.

Zero-point correction =	0.993397 (Hartree/Particle)
Thermal correction to Energy =	1.046753
Thermal correction to Enthalpy =	1.047697
Thermal correction to Gibbs Free Energy =	0.908359
Sum of electronic and zero-point Energies =	-2749.367320

Sum of electronic and thermal Energies = -2749.313963
Sum of electronic and thermal Enthalpies = -2749.313019
Sum of electronic and thermal Free Energies = -2749.452358

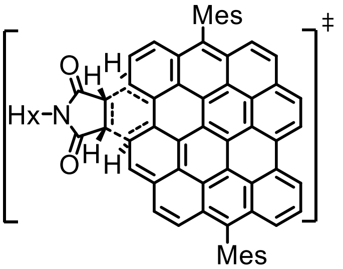

TS1

E(RwB97XD) = –2750.29551567 a.u.
Zero-point correction = 0.988371 (Hartree/Particle)
Thermal correction to Energy = 1.042449
Thermal correction to Enthalpy = 1.043393
Thermal correction to Gibbs Free Energy = 0.901713
Sum of electronic and zero-point Energies = -2749.307144
Sum of electronic and thermal Energies = -2749.253066
Sum of electronic and thermal Enthalpies = -2749.252122
Sum of electronic and thermal Free Energies = -2749.393803

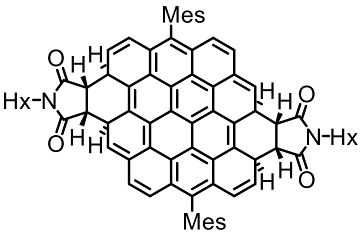

Int2

E(RwB97XD) = –3345.49973103 a.u.
Zero-point correction = 1.239772 (Hartree/Particle)
Thermal correction to Energy = 1.308783
Thermal correction to Enthalpy = 1.309727
Thermal correction to Gibbs Free Energy = 1.133374
Sum of electronic and zero-point Energies = -3344.259959
Sum of electronic and thermal Energies = -3344.190948
Sum of electronic and thermal Enthalpies = -3344.190004
Sum of electronic and thermal Free Energies = -3344.366357

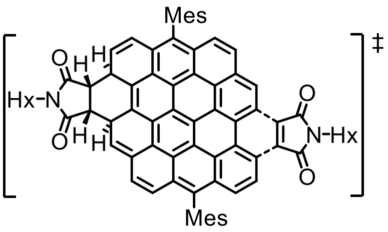

TS2

E(RwB97XD) = –3345.45167766 a.u.

Zero-point correction =	1.235987 (Hartree/Particle)
Thermal correction to Energy =	1.305403
Thermal correction to Enthalpy =	1.306348
Thermal correction to Gibbs Free Energy =	1.126894
Sum of electronic and zero-point Energies =	-3344.215691
Sum of electronic and thermal Energies =	-3344.146274
Sum of electronic and thermal Enthalpies =	-3344.145330
Sum of electronic and thermal Free Energies =	-3344.324784

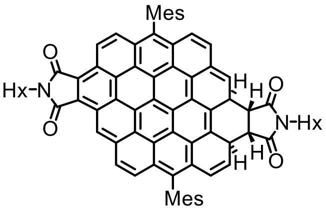

Int4

E(RwB97XD) = –3343.15596930 a.u.

Zero-point correction =	1.193352 (Hartree/Particle)
Thermal correction to Energy =	1.261613
Thermal correction to Enthalpy =	1.262557
Thermal correction to Gibbs Free Energy =	1.088424
Sum of electronic and zero-point Energies =	-3341.962617
Sum of electronic and thermal Energies =	-3341.894356
Sum of electronic and thermal Enthalpies =	-3341.893412
Sum of electronic and thermal Free Energies =	-3342.067545

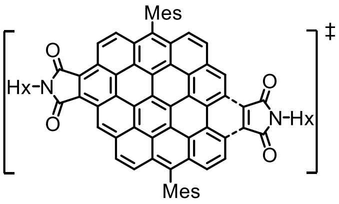

TS4

E(RwB97XD) = –3343.09497759 a.u.

Zero-point correction =	1.188500 (Hartree/Particle)
Thermal correction to Energy =	1.256768

Thermal correction to Enthalpy = 1.257712
Thermal correction to Gibbs Free Energy = 1.081715
Sum of electronic and zero-point Energies = -3341.906478
Sum of electronic and thermal Energies = -3341.838209
Sum of electronic and thermal Enthalpies = -3341.837265
Sum of electronic and thermal Free Energies = -3342.013262

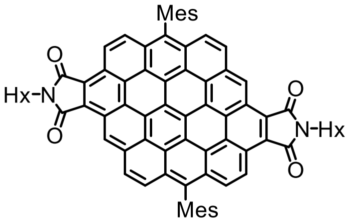

4a

E(RwB97XD) = –3340.79583652 a.u.
Zero-point correction = 1.146186 (Hartree/Particle)
Thermal correction to Energy = 1.214082
Thermal correction to Enthalpy = 1.215026
Thermal correction to Gibbs Free Energy = 1.041520
Sum of electronic and zero-point Energies = -3339.649650
Sum of electronic and thermal Energies = -3339.581755
Sum of electronic and thermal Enthalpies = -3339.580811
Sum of electronic and thermal Free Energies = -3339.754316

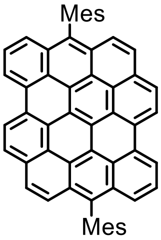

1

E(RwB97XD) = –2155.17964748 a.u.
Zero-point correction = 0.745070 (Hartree/Particle)
Thermal correction to Energy = 0.786791
Thermal correction to Enthalpy = 0.787735
Thermal correction to Gibbs Free Energy = 0.673799
Sum of electronic and zero-point Energies = -2154.434578
Sum of electronic and thermal Energies = -2154.392856
Sum of electronic and thermal Enthalpies = -2154.391912
Sum of electronic and thermal Free Energies = -2154.505848

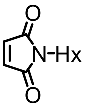

2a

E(RwB97XD) = –595.13900945 a.u.

Zero-point correction =	0.241925 (Hartree/Particle)
Thermal correction to Energy =	0.255376
Thermal correction to Enthalpy =	0.256320
Thermal correction to Gibbs Free Energy =	0.200566
Sum of electronic and zero-point Energies =	-594.897085
Sum of electronic and thermal Energies =	-594.883634
Sum of electronic and thermal Enthalpies =	-594.882690
Sum of electronic and thermal Free Energies =	-594.938443

H_2

E(RwB97XD) = –1.17196636 a.u.

Zero-point correction =	0.010117 (Hartree/Particle)
Thermal correction to Energy =	0.012477
Thermal correction to Enthalpy =	0.013421
Thermal correction to Gibbs Free Energy =	-0.001371
Sum of electronic and zero-point Energies =	-1.161850
Sum of electronic and thermal Energies =	-1.159489
Sum of electronic and thermal Enthalpies =	-1.158545
Sum of electronic and thermal Free Energies =	-1.173337

7.6 Energy and intrinsic reaction coordinates for the transition states (TS1, TS2 and TS3) involved in the two-folds Diels-Alder addition of DBOV-Mes (1) with N-*n*-hexylmaleimide (**2a**). **The properties were obtained using diphenyl ether as solvent with the polarizable continuum model (PCM) at the B3LYP/6-31G(d)//B3LYP/6-31G(d) level of theory with Gaussian 09 program (revision B.01).**

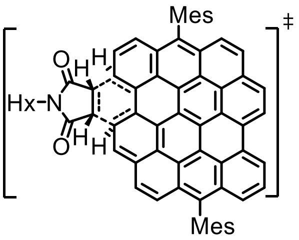

TS1

B3LYP/6-31g(d)

# Total Energy along IRC

X-Axis: Intrinsic Reaction Coordinate

Y-Axis: Total Energy (Hartree)

Energies reported relative to the TS energy of –2751.151091

Summary of reaction path following

	Energy	Rx Coord		Energy	Rx Coord
1	-0.021400	-3.073590	11	0.000000	0.000000
2	-0.017840	-2.765850	12	-0.000360	0.307410
3	-0.014440	-2.458030	13	-0.001450	0.615330
4	-0.011330	-2.150220	14	-0.003250	0.923280
5	-0.008570	-1.842510	15	-0.005620	1.231240
6	-0.006190	-1.535030	16	-0.008380	1.539210
7	-0.004180	-1.227920	17	-0.011330	1.847160
8	-0.002520	-0.921120	18	-0.014300	2.155100
9	-0.001200	-0.614250	19	-0.017180	2.463030
10	-0.000320	-0.307040	20	-0.019890	2.770960
			21	-0.022390	3.078880

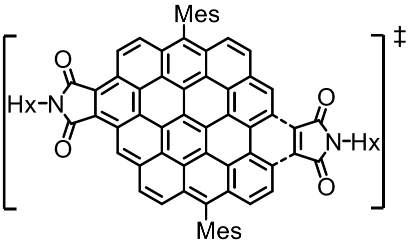

TS4

B3LYP/6-31g(d)

# Total Energy along IRC

X-Axis: Intrinsic Reaction Coordinate

Y-Axis: Total Energy (Hartree)

Energies reported relative to the TS energy of –3344.123770

Summary of reaction path following

	Energy	Rx Coord		Energy	Rx Coord
1	-0.023770	-3.059750	11	0.000000	0.000000
2	-0.020960	-2.753710	12	-0.000300	0.304910
3	-0.017950	-2.447660	13	-0.001120	0.610030
4	-0.014790	-2.141610	14	-0.002390	0.915050
5	-0.011570	-1.835550	15	-0.004070	1.220300
6	-0.008440	-1.529470	16	-0.006180	1.525900
7	-0.005580	-1.223390	17	-0.008710	1.831710
8	-0.003180	-0.917320	18	-0.011650	2.137630
9	-0.001400	-0.611250	19	-0.014910	2.443560
10	-0.000340	-0.305380	20	-0.018380	2.749450
			21	-0.021890	3.055230

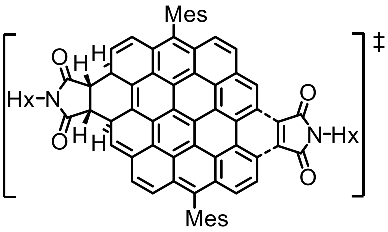

TS2

B3LYP/6-31g(d)

# Total Energy along IRC

X-Axis: Intrinsic Reaction Coordinate

Y-Axis: Total Energy (Hartree)

Energies reported relative to the TS energy of –3346.457295

Summary of reaction path following

	Energy	Rx Coord			
1	-0.020000	-3.042020	11	0.000000	0.000000
2	-0.017080	-2.738000	12	-0.000200	0.302510
3	-0.013960	-2.433960	13	-0.000830	0.606170
4	-0.010870	-2.129280	14	-0.002000	0.910320
5	-0.007990	-1.824630	15	-0.003770	1.214810
6	-0.005480	-1.519870	16	-0.006150	1.519530
7	-0.003420	-1.215170	17	-0.009050	1.824270
8	-0.001860	-0.910560	18	-0.012310	2.129010
9	-0.000800	-0.606150	19	-0.015770	2.433750
10	-0.000190	-0.302400	20	-0.019270	2.738480
			21	-0.022660	3.043200

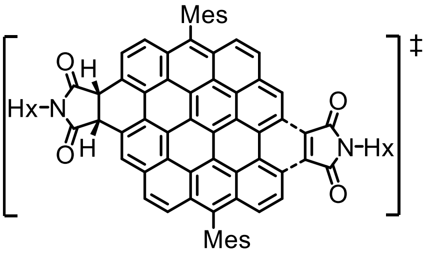

TS3

B3LYP/6-31g(d)

# Total Energy along IRC

X-Axis: Intrinsic Reaction Coordinate

Y-Axis: Total Energy (Hartree)

Energies reported relative to the TS energy of -3345.323228

Summary of reaction path following

	Energy	Rx Coord
1	-0.02159	-3.04503
2	-0.01904	-2.73682
3	-0.01629	-2.42858
4	-0.01339	-2.12034
5	-0.01044	-1.81208
6	-0.00757	-1.50382
7	-0.00495	-1.19554
8	-0.00276	-0.88727
9	-0.00118	-0.57921
10	-0.00029	-0.27723
11	0.00000	0.00000
12	-0.00007	0.03837
13	-0.00011	0.07706
14	-0.00044	0.32253
15	-0.00126	0.62894
16	-0.00244	0.93539
17	-0.00392	1.24205
18	-0.00574	1.54935
19	-0.00793	1.85717
20	-0.01053	2.16524
21	-0.01351	2.47338

8.6 Energy and intrinsic reaction coordinates for the transition states (TS1, TS2 and TS3) involved in the two-folds Diels-Alder addition of DBOV-Mes (1) with N-*n*-hexylmaleimide (**2a**). **The properties were obtained using diphenyl ether as solvent with the polarizable continuum model (PCM) at the M062X/6-31G(d)//B3LYP/6-31G(d) level of theory with Gaussian 09 program (revision B.01).**

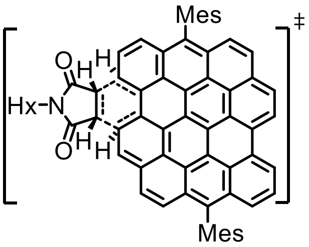

TS1

M062X/6-31g(d)

# Total Energy along IRC

X-Axis: Intrinsic Reaction Coordinate

Y-Axis: Total Energy (Hartree)

Energies reported relative to the TS energy of –2750.026231

Summary of reaction path following

	Energy	Rx Coord		Energy	Rx Coord
1	-0.006630	-1.890300	11	0.000000	0.000000
2	-0.005360	-1.594210	12	-0.000190	0.021240
3	-0.004340	-1.298190	13	-0.001210	0.142370
4	-0.003520	-1.001000	14	-0.001950	0.322360
5	-0.002770	-0.703650	15	-0.002640	0.574320
6	-0.002000	-0.415270	16	-0.003570	0.851050
7	-0.001250	-0.200610	17	-0.005220	1.148370
8	-0.000420	-0.059460	18	-0.007650	1.447680
9	-0.000060	-0.012380	19	-0.010740	1.747060
10	-0.000010	-0.001550	20	-0.014370	2.046440
			21	-0.018420	2.345820

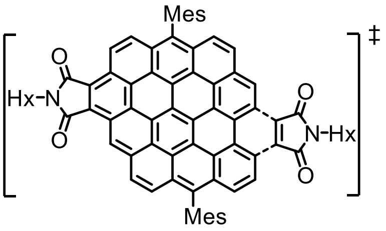

TS4

M062x/6-31g(d)

# Total Energy along IRC

X-Axis: Intrinsic Reaction Coordinate

Y-Axis: Total Energy (Hartree)

Energies reported relative to the TS energy of –3342.756407

Summary of reaction path following

	Energy	Rx Coord		Energy	Rx Coord
1	-0.003480	-1.086980	11	0.000000	0.000000
2	-0.003150	-0.838650	12	-0.000240	0.021500
3	-0.002750	-0.597600	13	-0.001400	0.135950
4	-0.002190	-0.355860	14	-0.002050	0.264040
5	-0.001620	-0.198410	15	-0.002900	0.494390
6	-0.000860	-0.083480	16	-0.004150	0.780970
7	-0.000340	-0.032550	17	-0.006070	1.078580
8	-0.000250	-0.021870	18	-0.008690	1.377560
9	-0.000030	-0.003740	19	-0.011940	1.676570
10	-0.000030	-0.002970	20	-0.015710	1.975630
			21	-0.019880	2.274710

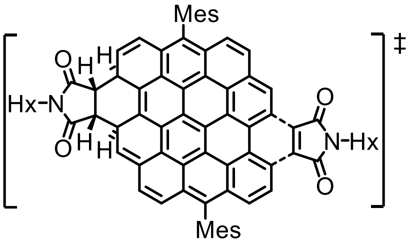

TS2

M062X/6-31g(d)

# Total Energy along IRC

X-Axis: Intrinsic Reaction Coordinate

Y-Axis: Total Energy (Hartree)

Energies reported relative to the TS energy of –3345.087177

Summary of reaction path following

	Energy	Rx Coord		Energy	Rx Coord
			11	0.000000	0.000000
1	-0.005380	-1.865070	12	-0.000210	0.020570
2	-0.004520	-1.565060	13	-0.001320	0.134150
3	-0.003820	-1.264950	14	-0.002190	0.315850
4	-0.003210	-0.965440	15	-0.003040	0.576900
5	-0.002610	-0.668300	16	-0.004070	0.865930
6	-0.001930	-0.385950	17	-0.005630	1.164390
7	-0.001170	-0.177590	18	-0.007850	1.464770
8	-0.000350	-0.048970	19	-0.010720	1.765340
9	-0.000090	-0.012120	20	-0.014150	2.066060
10	-0.000010	-0.001170	21	-0.017980	2.366840

8.8 Energy and intrinsic reaction coordinates for the transition states (TS1, TS2 and TS3) involved in the two-folds Diels-Alder addition of DBOV-Mes (1) with N-*n*-hexylmaleimide (**2a**). **The properties were obtained using diphenyl ether as solvent with the polarizable continuum model (PCM) at the wB97XD/6-31G(d)//B3LYP/6-31G(d) level of theory with Gaussian 09 program (revision B.01).**

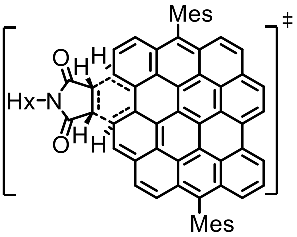

TS1

----

wB97XD/6-31G(d)

# Total Energy along IRC

X-Axis: Intrinsic Reaction Coordinate
Y-Axis: Total Energy (Hartree)

Energies reported relative to the TS energy of –2750.284809

Summary of reaction path following

	Energy	Rx Coord			
1	-0.029650	-2.634700	11	0.000000	0.000000
2	-0.024860	-2.337090	12	-0.000150	0.017680
3	-0.020290	-2.039540	13	-0.001270	0.141310
4	-0.016030	-1.742060	14	-0.001890	0.269680
5	-0.012160	-1.444650	15	-0.003030	0.530820
6	-0.008760	-1.147340	16	-0.004810	0.825120
7	-0.005910	-0.850170	17	-0.007310	1.122220
8	-0.003700	-0.555630	18	-0.010440	1.419480
9	-0.002150	-0.290860	19	-0.014100	1.716820
10	-0.000500	-0.077240	20	-0.018180	2.014230
			21	-0.022620	2.311710

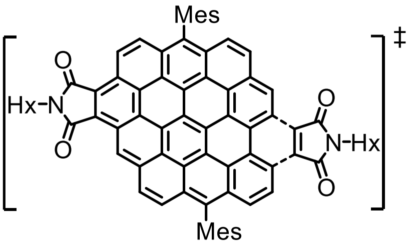

TS4

----

wB97XD/6-31G(d)

# Total Energy along IRC

X-Axis: Intrinsic Reaction Coordinate
Y-Axis: Total Energy (Hartree)

Energies reported relative to the TS energy of –3343.082797

Summary of reaction path following

	Energy	Rx Coord			
1	-0.016340	-1.929890	8	-0.000560	-0.070170
2	-0.012800	-1.646520	9	-0.000050	-0.011760
3	-0.009630	-1.356630	10	-0.000020	-0.001790
4	-0.006890	-1.064500	11	0.000000	0.000000
5	-0.004670	-0.771830	12	-0.000340	0.031320
6	-0.002970	-0.484980	13	-0.001820	0.188050
7	-0.001630	-0.226830	14	-0.002830	0.383990
			15	-0.004490	0.671840

16	-0.006830	0.967370	19	-0.017410	1.856170
17	-0.009830	1.263510	20	-0.021820	2.152590
18	-0.013380	1.559810	21	-0.026490	2.449050

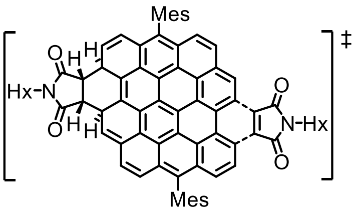

TS2

----

wB97XD/6-31G(d)

# Total Energy along IRC

X-Axis: Intrinsic Reaction Coordinate

Y-Axis: Total Energy (Hartree)

Energies reported relative to the TS energy of –3345.437272

Summary of reaction path following

	Energy	Rx Coord			
1	-0.026610	-2.603190	11	0.000000	0.000000
2	-0.022250	-2.303500	12	-0.000160	0.016150
3	-0.017980	-2.003820	13	-0.001450	0.143280
4	-0.013990	-1.704170	14	-0.002130	0.273000
5	-0.010460	-1.404600	15	-0.003120	0.518740
6	-0.007510	-1.105140	16	-0.004570	0.813380
7	-0.005180	-0.806990	17	-0.006640	1.111420
8	-0.003450	-0.512250	18	-0.009370	1.410840
9	-0.002240	-0.275610	19	-0.012720	1.710410
10	-0.000470	-0.067320	20	-0.016560	2.010070
			21	-0.020750	2.309760

8.9 Cartesian coordinates of optimized geometries of 3a, 4a, 1 and CP by Gaussian 16

Cartesian coordinates of optimized geometry of 3a (*n*-hexyl replaced by Me-, xyz format; number of atoms: 101)

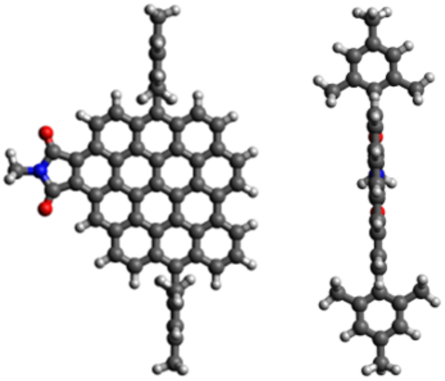

E(B3LYP) = –2552.33812248 a.u.
LUMO = – 2.59 eV
HOMO = – 4.85 eV

	x	y	z
C	-4.40627	2.515859	-0.00025
C	-3.01327	2.827911	-0.00029
C	-2.05176	1.771021	-0.00020
C	-2.50023	0.412282	-0.000095
C	-3.90392	0.120618	-0.000083
C	-4.82785	1.218281	-0.00015
C	-0.64893	2.057812	-0.00022
C	0.298678	0.992223	-0.00012
C	-0.14818	-0.36357	0.000006
C	-1.55497	-0.64631	0.000001
C	-0.17719	3.412923	-0.00035
C	1.209788	3.677627	-0.00037
C	2.135086	2.647075	-0.00027
C	1.691975	1.278172	-0.00013
C	3.551569	2.910423	-0.00029
C	4.458281	1.903944	-0.00017
C	4.053629	0.515168	0.000003
C	2.639363	0.219283	0.000004
C	0.79328	-1.41830	0.000128
C	2.19414	-1.12825	0.000137
C	3.147394	-2.19004	0.000280
C	2.713743	-3.55367	0.000403
C	1.281961	-3.84926	0.000378
C	0.345342	-2.78109	0.000238
C	4.549152	-1.88309	0.000298
C	5.479105	-2.94716	0.000453
C	5.047332	-4.26169	0.000572
C	-0.56373	-5.44378	0.000435
C	-1.51343	-4.40551	0.000291
C	-1.05714	-3.05786	0.000207
C	-2.92025	-4.66532	0.000223
C	-3.82830	-3.65078	0.000112
C	-3.41299	-2.27473	0.000067
C	-2.00714	-1.98995	0.000090
C	-4.35192	-1.22089	-0.000005

C	4.988661	-0.51632	0.000156
C	-2.51404	4.148323	-0.00042
C	-1.16149	4.430006	-0.00045
C	-3.26278	5.442824	-0.00053
N	-2.28481	6.446342	-0.00056
C	-0.99000	5.911528	-0.00058
O	-4.46428	5.644862	-0.00046
O	0.028382	6.581271	-0.00058
C	-2.57253	7.867726	-0.00058
C	-5.82246	-1.52306	0.000005
C	-6.51574	-1.66233	-1.22137
C	-7.88644	-1.94007	-1.19779
C	-8.59108	-2.08613	0.000073
C	-7.88621	-1.94078	1.197968
C	-6.51557	-1.66307	1.221470
C	-5.80113	-1.51203	2.545410
C	-5.80161	-1.51032	-2.54537
C	-10.0649	-2.41665	0.000270
C	6.458896	-0.21403	0.000163
C	7.152667	-0.07702	-1.22128
C	8.524154	0.196576	-1.19777
C	9.229290	0.340738	0.000145
C	8.524067	0.197224	1.197999
C	7.152512	-0.07638	1.221537
C	6.436304	-0.21963	2.545363
C	6.436545	-0.22107	-2.54506
C	10.70384	0.668152	-0.000049
H	-5.11446	3.335970	-0.00030
H	-5.88884	0.995730	-0.00010
H	1.538980	4.710791	-0.00048
H	3.877692	3.946627	-0.00042
H	5.519661	2.124609	-0.00019
H	6.538705	-2.71957	0.000482
H	5.773216	-5.06938	0.000692
H	-0.90906	-6.47402	0.000508
H	-3.25441	-5.69941	0.000251

H	-4.89044	-3.86738	0.00004	H	9.054228	0.298126	-2.14228
H	-3.65695	7.981251	-0.00056	H	9.054048	0.299296	2.142497
H	-2.14924	8.345194	0.887282	H	7.134520	-0.11354	3.379623
H	-2.14929	8.345154	-0.88849	H	5.653657	0.537796	2.662490
H	-8.41647	-2.04212	-2.14224	H	5.947524	-1.19560	2.635057
H	-8.41608	-2.04337	2.142455	H	5.653298	0.535703	-2.66232
H	-6.49262	-1.65496	3.379734	H	7.134664	-0.11462	-3.37936
H	-4.98991	-2.24051	2.650435	H	5.948527	-1.19745	-2.63455
H	-5.34961	-0.51936	2.647849	H	11.20512	0.257982	-0.88186
H	-4.98913	-2.23738	-2.65030	H	10.86908	1.752858	-0.00808
H	-6.49290	-1.65457	-3.37964	H	11.20238	0.271275	0.889309
H	-5.35184	-0.51686	-2.64801	C	3.684180	-4.56317	0.000543
H	-10.5643	-2.01980	-0.88858	H	3.388861	-5.60523	0.000624
H	-10.2282	-3.50171	0.007454	C	0.788884	-5.17217	0.000482
H	-10.5668	-2.00794	0.882407	H	1.479072	-6.00690	0.000609

Cartesian coordinates of optimized geometry of **4a** (*n*-hexyl replaced by Me-, xyz format; number of atoms: 110)

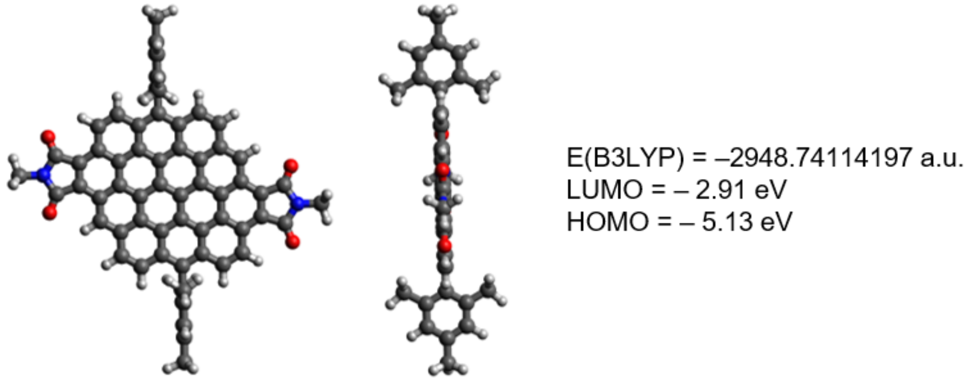

	x	y	z				
C	-5.45418	2.024202	-0.00107	C	3.225163	2.332744	-0.000003
C	-4.19946	2.695252	-0.00100	C	1.943106	1.682778	-0.00003
C	-2.99076	1.933177	-0.00063	C	0.594104	-0.38368	0.000176
C	-3.06234	0.505042	-0.00030	C	1.86698	0.264872	0.000143
C	-4.33389	-0.15044	-0.00029	C	3.062343	-0.50506	0.000261
C	-5.51382	0.656728	-0.00071	C	2.99077	-1.93319	0.000426
C	-1.71491	2.584543	-0.00058	C	1.714921	-2.58456	0.000532
C	-0.52054	1.810578	-0.00028	C	0.520547	-1.81059	0.00042
C	-0.59410	0.383671	-0.000029	C	4.333895	0.150426	0.000215
C	-1.86697	-0.26488	0.000003	C	5.513825	-0.65674	0.000256
C	-1.61841	4.017056	-0.00082	C	5.454186	-2.02421	0.000363
C	-0.35480	4.639129	-0.00074	C	4.199462	-2.69527	0.000474
C	0.815174	3.888511	-0.00044	C	1.618412	-4.01707	0.00076
C	0.747135	2.454478	-0.00024	C	0.354806	-4.63914	0.000926
C	2.111502	4.511494	-0.00034	C	-0.81517	-3.88853	0.00083
C	3.252949	3.775764	-0.00012	C	-0.74713	-2.45449	0.000528
				C	-2.11150	-4.51151	0.00103

C	-3.25294	-3.77578	0.000852
C	-3.22515	-2.33276	0.000429
C	-1.94310	-1.68279	0.000325
C	-4.40525	-1.57215	0.000111
C	4.405264	1.572124	0.000114
C	-4.06775	4.10492	-0.00127
C	-2.84217	4.735432	-0.00118
C	4.067754	-4.10493	0.000655
C	2.842175	-4.73544	0.000811
C	-5.13545	5.153662	-0.00163
N	-4.46128	6.381858	-0.00166
C	-3.07169	6.210769	-0.00154
C	5.135457	-5.15367	0.000681
N	4.461284	-6.38187	0.00086
C	3.071692	-6.21078	0.000998
O	-6.34649	5.025603	-0.00178
O	-2.26629	7.124967	-0.00141
O	2.266292	-7.12498	0.00112
O	6.346494	-5.02561	0.000571
C	-5.11788	7.675401	-0.00192
C	5.11789	-7.67541	0.000943
C	-5.74378	-2.25196	0.000197
C	-6.37526	-2.56909	-1.22149
C	-7.62429	-3.19803	-1.19761
C	-8.26563	-3.52427	0.000391
C	-7.62414	-3.19797	1.198276
C	-6.37509	-2.56903	1.221976
C	-5.72467	-2.23771	2.546122
C	-5.72518	-2.23739	-2.54571
C	-9.60017	-4.23152	0.000489
C	5.743776	2.25196	0.000141
C	6.375467	2.568586	-1.22157
C	7.624475	3.197574	-1.19773
C	8.265593	3.524343	0.000239
C	7.623904	3.198531	1.198149
C	6.37487	2.569555	1.221899
C	5.724245	2.238734	2.546069
C	5.725618	2.23634	-2.54577
C	9.600111	4.23163	0.000254

H	-6.35720	2.62314	-0.00140
H	-6.47749	0.160221	-0.00074
H	-0.31044	5.722641	-0.00091
H	2.157348	5.596812	-0.00044
H	4.218891	4.267815	-0.000045
H	6.477496	-0.16023	0.000211
H	6.357206	-2.62315	0.000364
H	0.310442	-5.72265	0.001124
H	-2.15734	-5.59683	0.001342
H	-4.21889	-4.26782	0.001026
H	-6.19328	7.495666	-0.00200
H	-4.83738	8.24817	0.886062
H	-4.83719	8.247912	-0.89000
H	6.193286	-7.49568	0.000775
H	4.837412	-8.24794	0.889086
H	4.837174	-8.24817	-0.88698
H	-8.10839	-3.43689	-2.14196
H	-8.10813	-3.43673	2.142707
H	-6.36036	-2.54597	3.380015
H	-4.75740	-2.73990	2.656237
H	-5.53635	-1.16324	2.645157
H	-4.75652	-2.73701	-2.65501
H	-6.35975	-2.54809	-3.37956
H	-5.53978	-1.16248	-2.64567
H	10.18910	-3.97533	-0.88519
H	-9.47114	-5.32114	0.000813
H	10.18923	-3.97482	0.885933
H	8.108725	3.436033	-2.14210
H	8.107723	3.437712	2.142558
H	6.359811	2.547297	3.379943
H	4.756961	2.740976	2.655863
H	5.535905	1.164306	2.645464
H	4.757001	2.735974	-2.65546
H	6.360359	2.546642	-3.37963
H	5.540172	1.161395	-2.64529
H	10.18986	3.97382	-0.88441
H	9.47104	5.321248	-0.00159
H	10.18837	3.976594	0.886707

Cartesian coordinates of optimized geometry of **CP** (xyz format; number of atoms: 96)

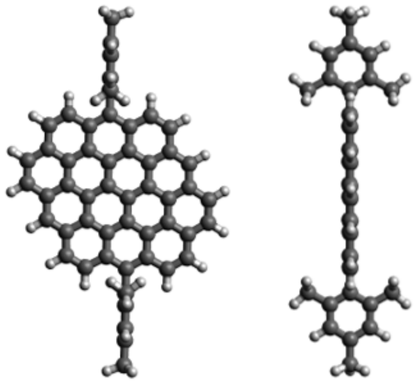

E(B3LYP) = –2308.41725028 a.u.
LUMO = – 2.22 eV
HOMO = – 4.70 eV

	x	y	z
C	4.448231	-3.71760	0.000061
C	3.042607	-3.93610	0.000046
C	2.178357	-2.80891	0.000032
C	2.723197	-1.48715	0.000023
C	4.13961	-1.29338	0.000022
C	4.976197	-2.45299	0.000054
C	0.76330	-3.00102	0.000029
C	-0.10910	-1.87860	0.000021
C	0.433417	-0.55735	0.000018
C	1.850674	-0.36542	0.000017
C	0.220719	-4.32259	0.000032
C	-1.17892	-4.48900	0.000025
C	-2.04827	-3.40266	0.000017
C	-1.51675	-2.06941	0.000018
C	-3.47853	-3.56135	-0.000002
C	-4.31504	-2.49061	-0.000012
C	-3.81711	-1.13484	0.000007
C	-2.39180	-0.94630	0.000013
C	-0.43342	0.557347	0.000014
C	-1.85067	0.365421	0.000011
C	-2.72320	1.487151	0.000007
C	-2.17836	2.808912	0.00001
C	-0.76330	3.001025	0.000010
C	0.109103	1.878599	0.000011
C	-4.13961	1.293382	0.000003
C	-4.97620	2.452991	0.000025
C	-4.44823	3.717605	0.000025
C	-3.04261	3.936106	0.000014
C	-0.22072	4.322592	0.000008
C	1.178922	4.488999	0.000002
C	2.048269	3.402662	0.000000
C	1.516749	2.069414	0.000009
C	3.478525	3.561355	-0.000021
C	4.315035	2.490609	-0.000028
C	3.817110	1.134841	0.000002
C	2.391798	0.946303	0.000011
C	4.677999	0.025862	0.000004
C	-4.67800	-0.02586	0.00000
C	2.476103	-5.24364	0.00005
C	1.119481	-5.43151	0.000043
C	-2.47610	5.243637	0.000014
C	-1.11948	5.431511	0.00001
C	6.166319	0.222390	-0.000017
C	6.868521	0.310566	-1.22109
C	8.255888	0.487573	-1.19789
C	8.969384	0.582167	-0.000054
C	8.255829	0.488284	1.197799
C	6.868461	0.311292	1.221035
C	6.144043	0.215908	2.544701
C	6.144187	0.214257	-2.54473
C	10.4633	0.805787	-0.000078
C	-6.16632	-0.22239	-0.000009
C	-6.86852	-0.31063	-1.22108
C	-8.25588	-0.48764	-1.19787
C	-8.96938	-0.58217	-0.000031
C	-8.25583	-0.48823	1.197812
C	-6.86846	-0.31123	1.221041
C	-6.14405	-0.21578	2.544706
C	-6.14418	-0.21439	-2.54473
C	-10.4633	-0.8058	-0.00008
H	5.108823	-4.58075	0.000085
H	6.051673	-2.31502	0.000083
H	-1.58853	-5.49634	0.000024
H	-3.88305	-4.5701	-0.000015
H	-5.38905	-2.64009	-0.000045
H	-6.05167	2.315026	0.000053
H	-5.10882	4.580747	0.000042
H	1.588527	5.496345	-0.000004
H	3.883047	4.570102	-0.000043
H	5.389051	2.64009	-0.000066
H	8.791797	0.551529	-2.14247

H	8.791692	0.552783	2.142371
H	6.845586	0.292442	3.379519
H	5.400519	1.012784	2.653783
H	5.605538	-0.73296	2.640848
H	5.39949	1.010033	-2.65373
H	6.84559	0.291833	-3.37957
H	5.607072	-0.73540	-2.64092
H	10.93382	0.368107	-0.88563
H	10.70453	1.876267	-0.00011
H	10.93384	0.368159	0.885494
H	-8.79179	-0.55165	-2.14246
H	-8.79170	-0.55268	2.142386
H	-6.84560	-0.29224	3.379524
H	-5.40054	-1.01267	2.653844
H	-5.60552	0.733080	2.640797
H	-5.39951	-1.01020	-2.65369
H	-6.84559	-0.29198	-3.37957
H	-5.60703	0.735244	-2.64095
H	-10.934	-0.36684	-0.88491
H	-10.7045	-1.87628	-0.00164
H	-10.9337	-0.36945	0.886215
H	-3.14641	6.099087	0.000017
H	-0.70480	6.435889	0.000009
H	0.704801	-6.43589	0.000046
H	3.146414	-6.09909	0.000060

Cartesian coordinates of optimized geometry of DBOV-Mes **1** (xyz format; number of atoms: 92)

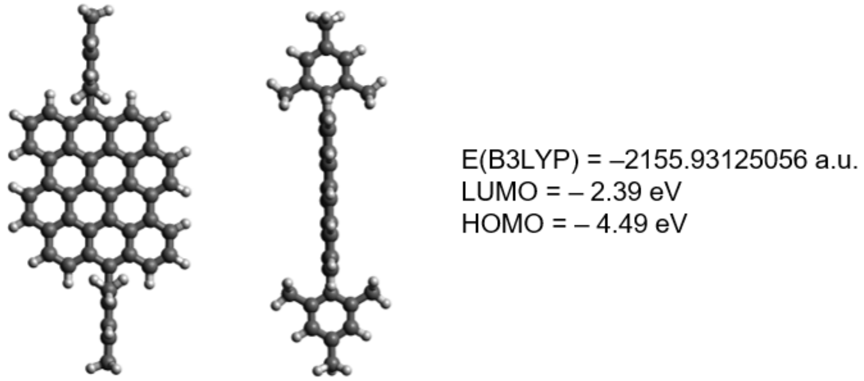

	x	y	z
C	4.365346	-3.83972	0.000049
C	2.973477	-3.99708	0.000044
C	2.115248	-2.89613	0.000024
C	2.685139	-1.58178	0.000014
C	4.113233	-1.42233	0.000013
C	4.930385	-2.58203	0.000032
C	0.657499	-3.04192	0.000014
C	-0.16708	-1.88171	0.000011
C	0.413824	-0.57079	0.000011
C	1.846754	-0.42999	0.000008
C	0.036838	-4.30449	0.000006
C	-1.33950	-4.44008	-0.000002
C	-2.17672	-3.31551	-0.000002
C	-1.58950	-2.01751	0.000005
C	-3.60574	-3.43879	-0.000012
C	-4.40873	-2.34431	-0.000012
C	-3.86160	-1.01013	0.000001
C	-2.42747	-0.85944	0.000006
C	-0.41382	0.570792	0.000011
C	-1.84675	0.429993	0.000010
C	-2.68514	1.581780	0.000016
C	-2.11525	2.896132	0.000024
C	-0.65750	3.041917	0.000013
C	0.167084	1.881713	0.000008
C	-4.11323	1.422326	0.000018
C	-4.93039	2.582029	0.000036
C	-4.36535	3.839718	0.000048
C	-2.97348	3.997079	0.000041
C	-0.03684	4.304486	0.000004
C	1.339497	4.440083	-0.000007
C	2.176723	3.315505	-0.000009
C	1.589504	2.017511	0.000000
C	3.605741	3.438792	-0.000023
C	4.408730	2.344306	-0.000024
C	3.861603	1.010127	-0.000008
C	2.427469	0.859439	0.000000
C	4.689940	-0.11653	-0.000002
C	-4.68994	0.116530	0.000006
C	6.182989	0.037663	-0.000011
C	6.887552	0.106476	-1.22101
C	8.279326	0.244685	-1.19784

C	8.99526	0.319055	-0.000027
C	8.279326	0.244809	1.197791
C	6.887555	0.106605	1.220978
C	6.160494	0.031919	2.544461
C	6.160493	0.031631	-2.54448
C	10.49486	0.500727	-0.000032
C	-6.18299	-0.03766	-0.000001
C	-6.88755	-0.10648	-1.22100
C	-8.27932	-0.24469	-1.19784
C	-8.99526	-0.31906	-0.000022
C	-8.27933	-0.24480	1.197791
C	-6.88755	-0.10660	1.220981
C	-6.16050	-0.03191	2.544468
C	-6.16049	-0.03164	-2.54448
C	-10.4949	-0.50073	-0.000061
H	5.001255	-4.72017	0.000066
H	6.007783	-2.46444	0.000037
H	-1.78552	-5.43098	-0.000009
H	-4.03625	-4.43670	-0.000022
H	-5.48702	-2.45564	-0.000023
H	-6.00778	2.464438	0.000043
H	-5.00126	4.720166	0.000064
H	1.785521	5.430982	-0.000014
H	4.036252	4.436700	-0.000034
H	5.487018	2.455643	-0.000038
H	8.816776	0.293761	-2.14245
H	8.816779	0.293979	2.142398
H	6.864463	0.079797	3.379399
H	5.446376	0.854851	2.656926
H	5.588171	-0.89728	2.636519
H	5.446168	0.854384	-2.65693
H	6.86444	0.079685	-3.37943
H	5.58840	-0.89771	-2.63654
H	10.95292	0.050212	-0.88569
H	10.76605	1.564018	0.000170
H	10.95297	0.049871	0.885430
H	-8.81677	-0.29377	-2.14245
H	-8.81679	-0.29397	2.142397
H	-6.86447	-0.07978	3.379402
H	-5.44638	-0.85484	2.656942
H	-5.58818	0.897293	2.636524
H	-5.44616	-0.8544	-2.65692
H	-6.86443	-0.0797	-3.37942
H	-5.58839	0.897697	-2.63653
H	-10.9530	-0.04901	-0.88505
H	-10.7661	-1.56402	-0.00128
H	-10.9528	-0.05107	0.886071
H	2.572781	-5.00333	0.000058
H	0.639295	-5.20461	0.000004
H	-0.63930	5.204609	0.000004
H	-2.57278	5.003334	0.000052
