# Supplementary material for: Diels-Alder reaction affords circumpyrene tetracarboxydiimide with excited state intramolecular charge transfer character
Source: Commun Chem. 2026 Feb 28;9:122. doi: 10.1038/s42004-026-01946-3 (PMC12992557; doi:10.1038/s42004-026-01946-3)
Supplement: Supplementary file 3 — Description of Additional Supplementary Files [file 42004_2026_1946_MOESM3_ESM.pdf]

Description of Additional Supplementary Files:

File name: Supplementary Data 1

Description: NMR spectra of intermediates 2a-c and products 3a-3c and 4a-4b

File name: Supplementary Data 2

Description: Source data of all the graphs shown in Figure 4

File name: Supplementary Data 3

Description: Source data of all the graphs shown in Figure 5
