# Supplementary material for: Diels-Alder reaction affords circumpyrene tetracarboxydiimide with excited state intramolecular charge transfer character
Source: Commun Chem. 2026 Feb 28;9:122. doi: 10.1038/s42004-026-01946-3 (PMC12992557; doi:10.1038/s42004-026-01946-3)
Supplement: Supplementary file 4 — Supplementary Data 1 [file 42004_2026_1946_MOESM4_ESM.pdf]

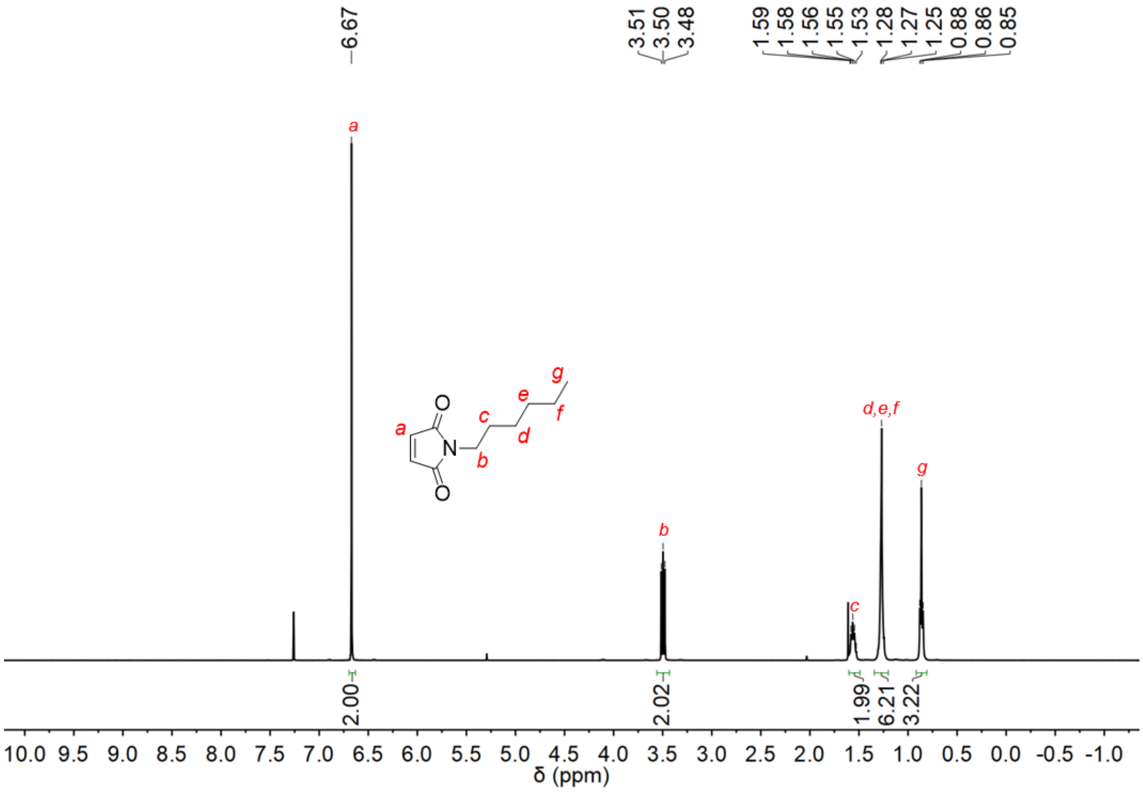

^{1}H NMR spectrum of *N*-*n*-hexylmaleimide (**2a**) ($CDCl_3$, 400 MHz, 298 K).

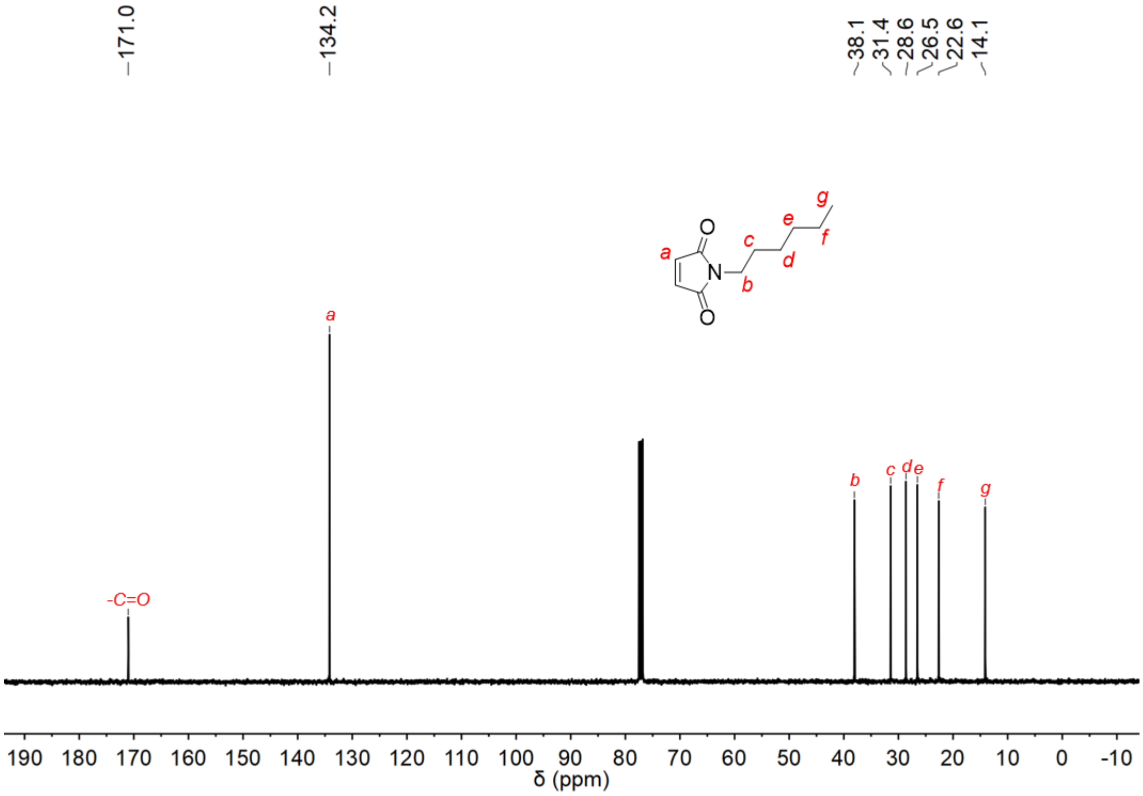

^{13}C NMR spectrum of *N*-*n*-hexylmaleimide (**2a**) ($CDCl_3$, 100 MHz, 298 K).

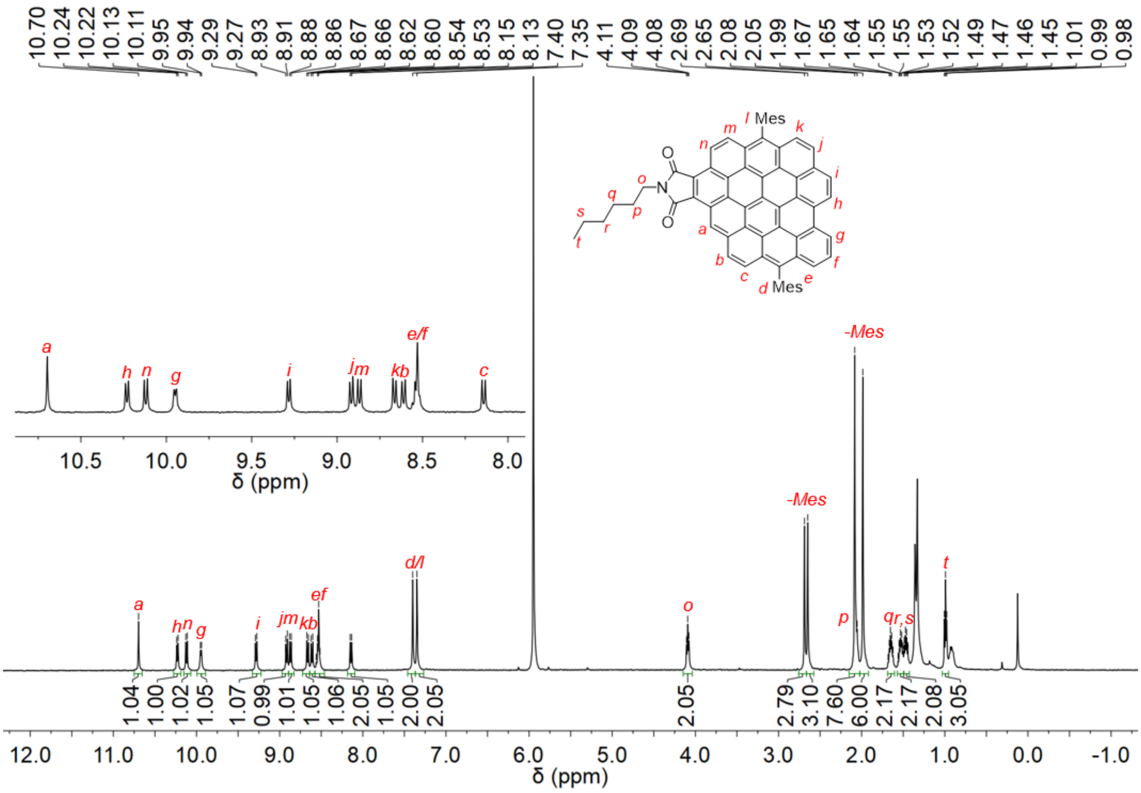

^{1}H NMR spectrum of mono-adduct **3a** ($C_2D_2Cl_4$, 500 MHz, 413 K).

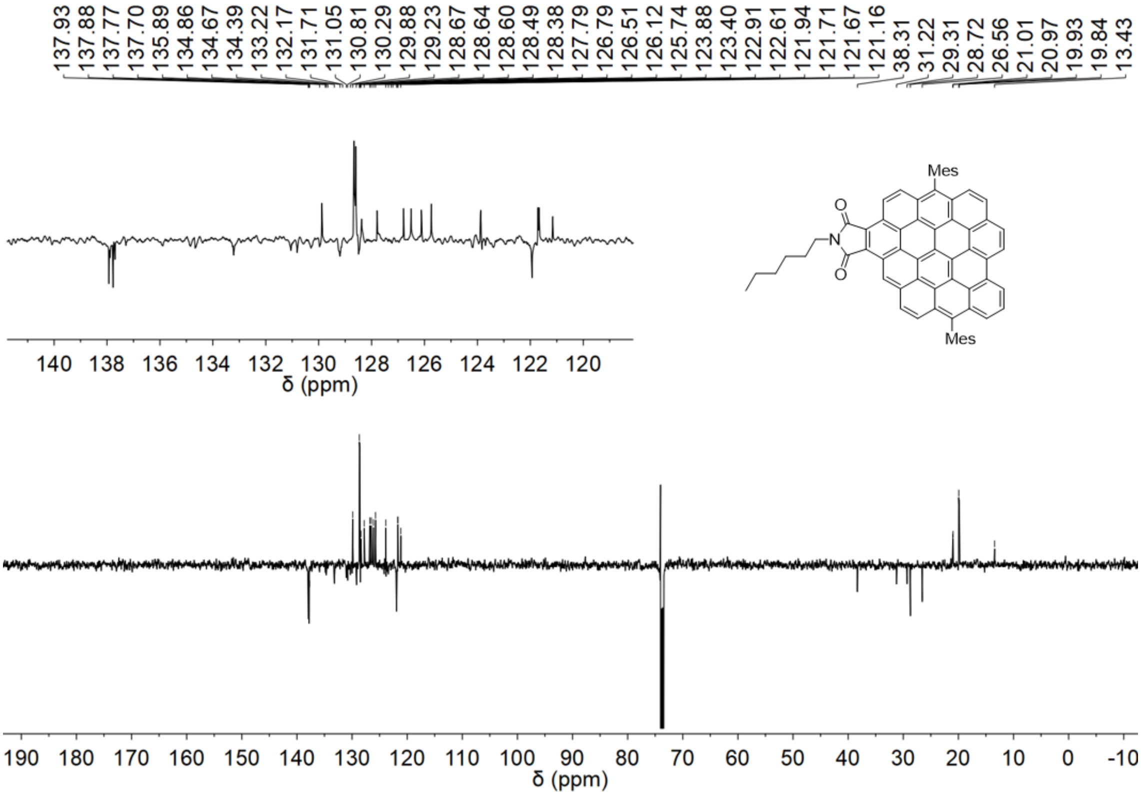

APT ^{13}C NMR of mono-adduct **3a** ($C_2D_2Cl_4$, 125 MHz, 413 K).

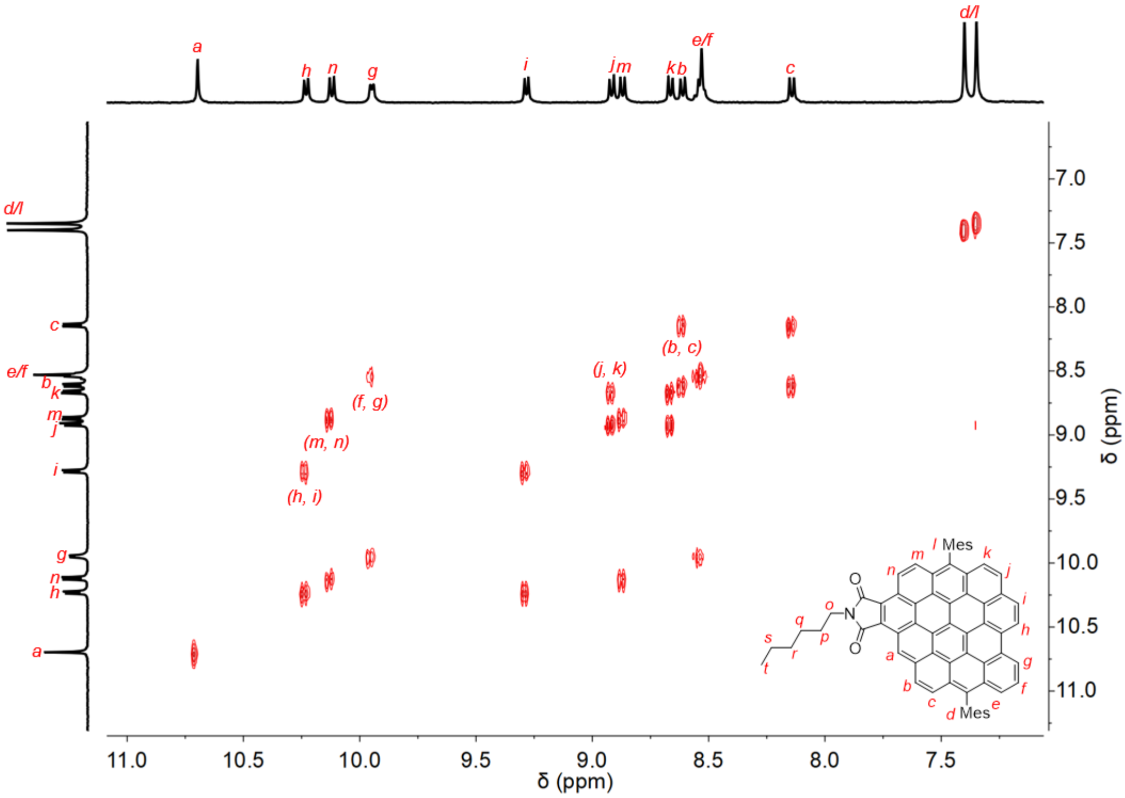

Aromatic region of ^{1}H,^{1}H-COSY spectrum of mono-adduct **3a** ($C_2D_2Cl_4$, 500 MHz, 413 K).

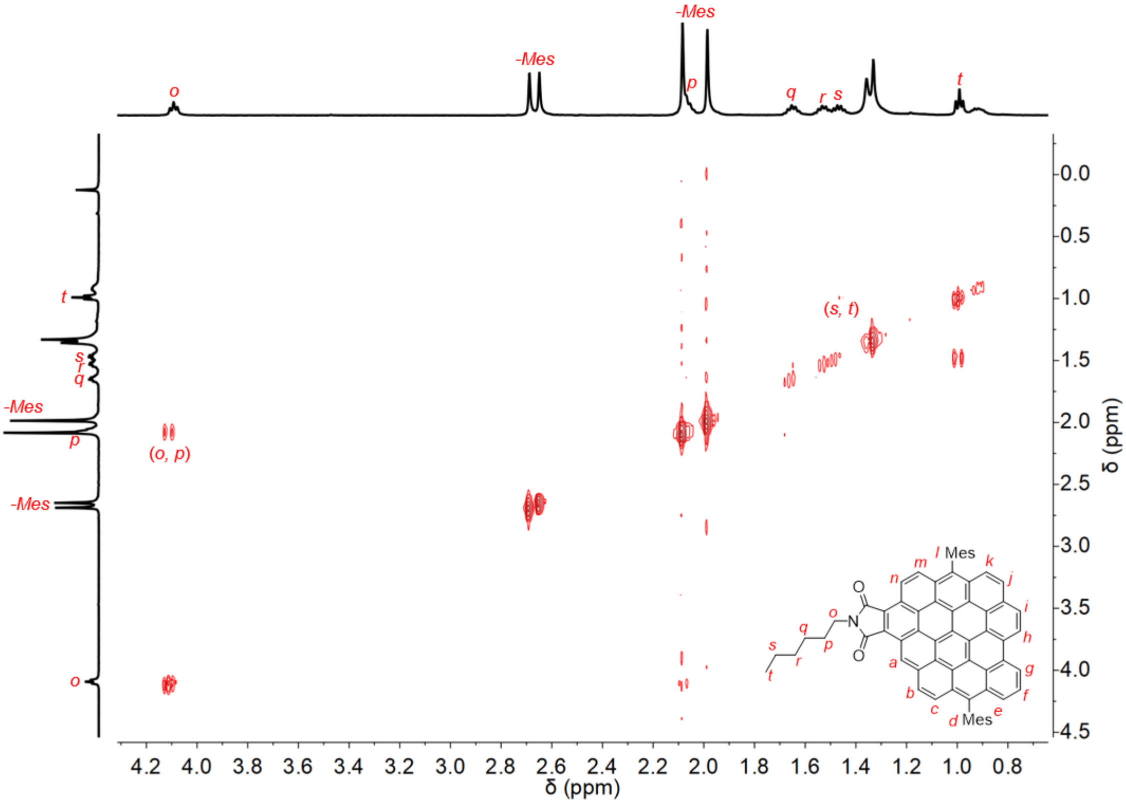

Aliphatic region of ^{1}H,^{1}H-COSY spectrum of mono-adduct **3a** ($C_2D_2Cl_4$, 500 MHz, 413 K).

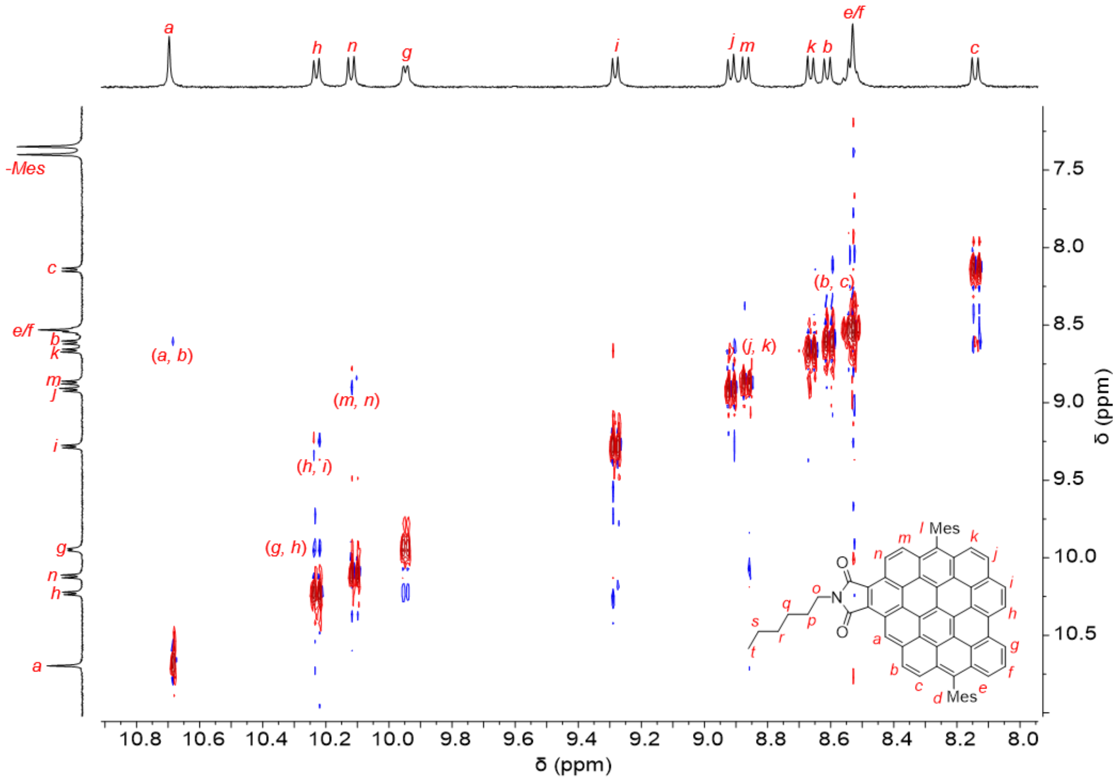

Aromatic region of ^{1}H,^{1}H-NOE spectrum of mono-adduct **3a** ($C_2D_2Cl_4$, 500 MHz, 413 K).

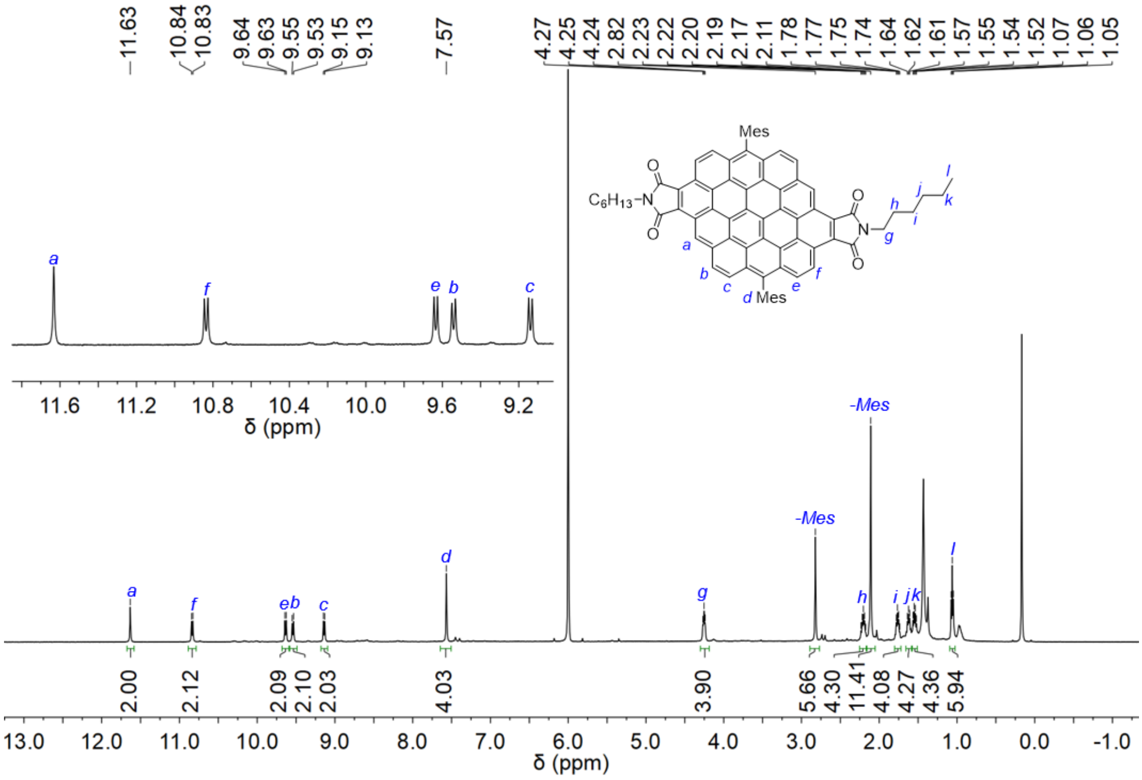

^{1}H NMR spectrum of di-adduct **4a** ($C_2D_2Cl_4$, 500 MHz, 413 K).

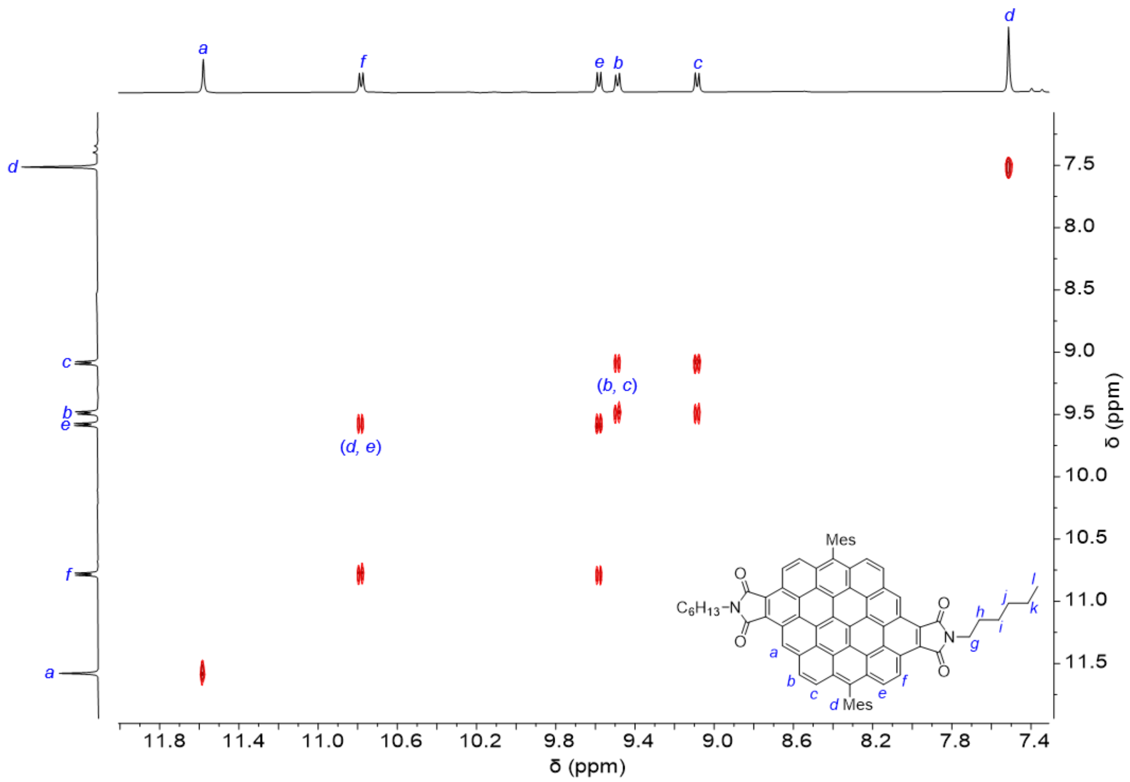

Aromatic region of ^{1}H,^{1}H-COSY spectrum of di-adduct **4a** ($C_2D_2Cl_4$, 500 MHz, 413 K).

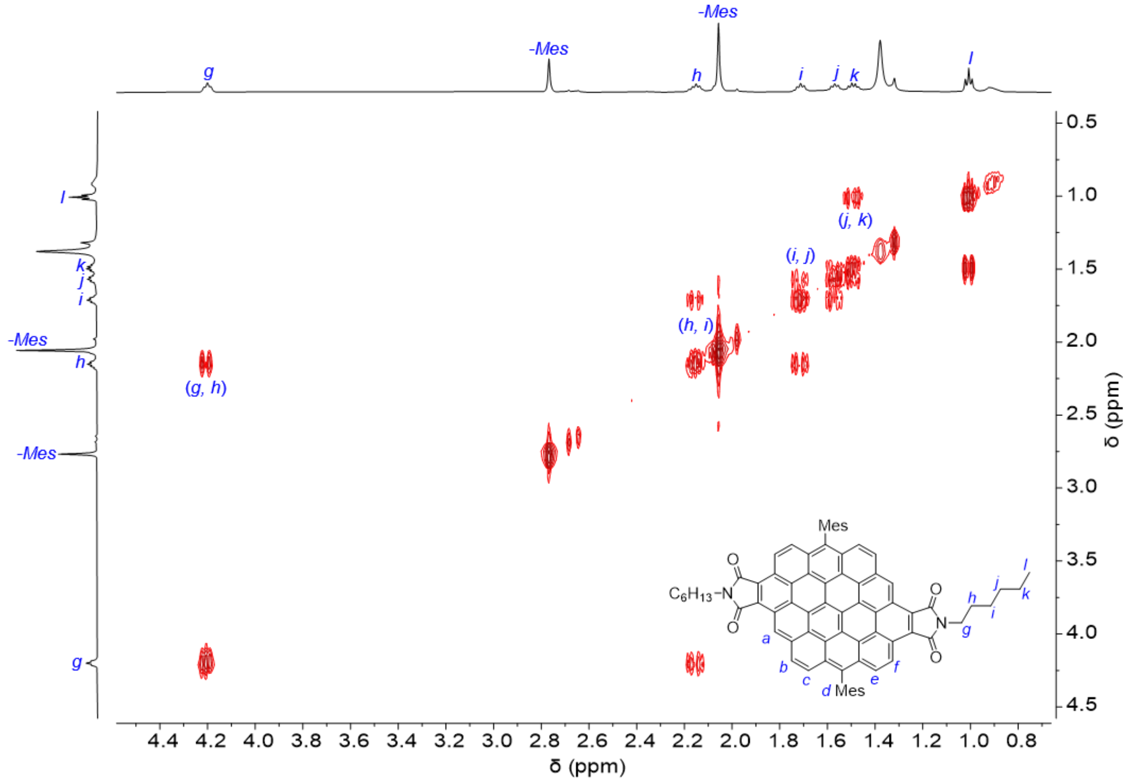

Aliphatic region of ^{1}H,^{1}H-COSY spectrum of di-adduct **4a** ($C_2D_2Cl_4$, 500 MHz, 413 K).

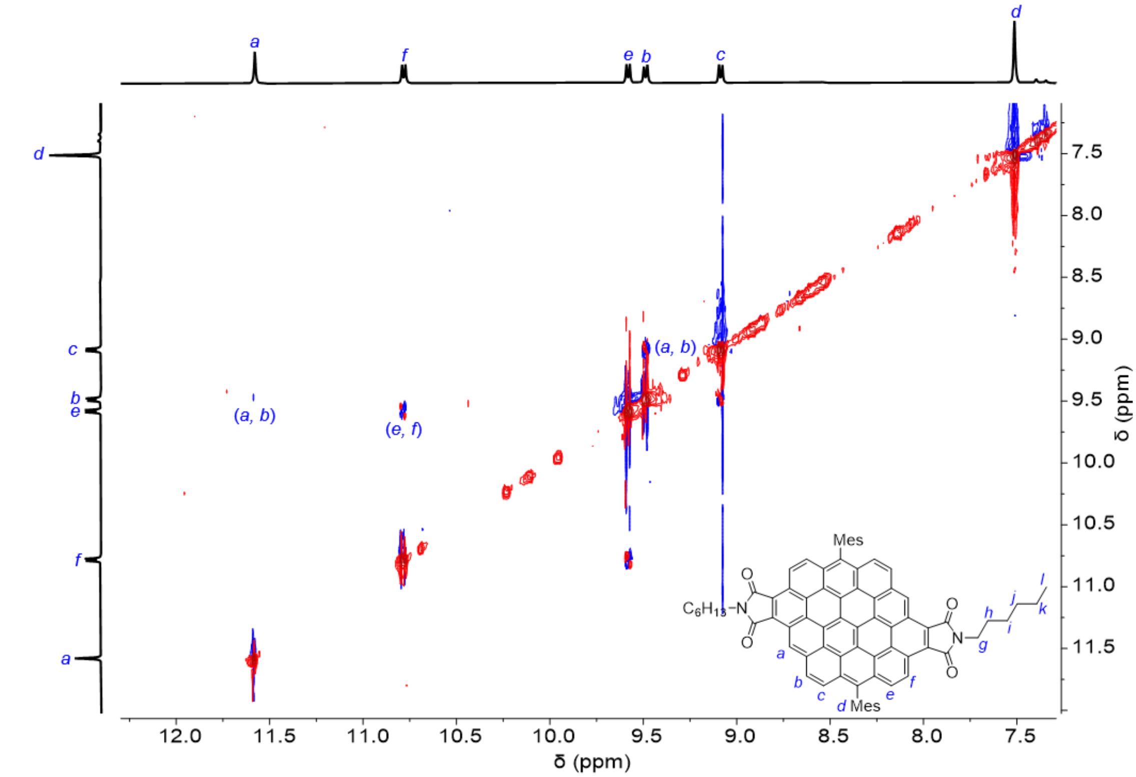

Aromatic region of ^{1}H,^{1}H-NOE spectrum of di-adduct **4a** ($C_2D_2Cl_4$, 500 MHz, 413 K).

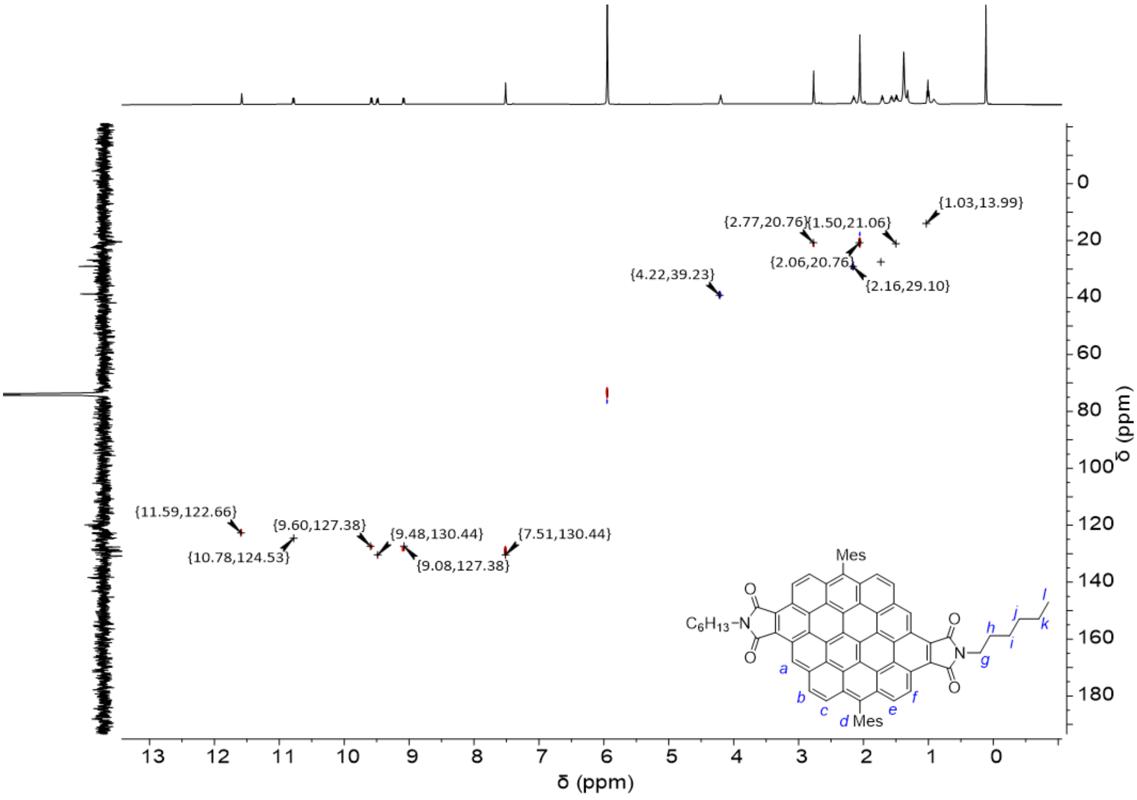

^{1}H,^{13}C-HSQC spectrum of di-adduct **4a** ($C_2D_2Cl_4$, 500 MHz, 413 K).

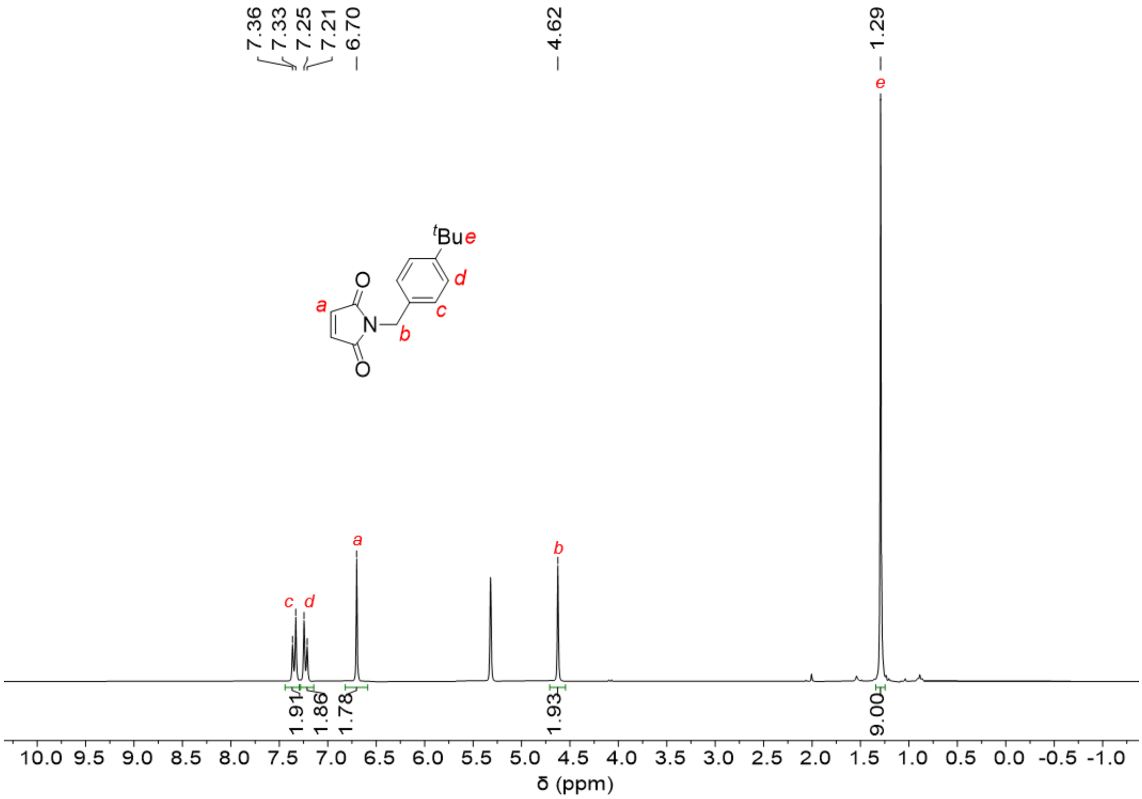

^{1}H NMR spectrum of *N*-(4-*tert*-butylbenzyl)maleimide (**2b**) (CD_2Cl_2, 250 MHz, 298 K).

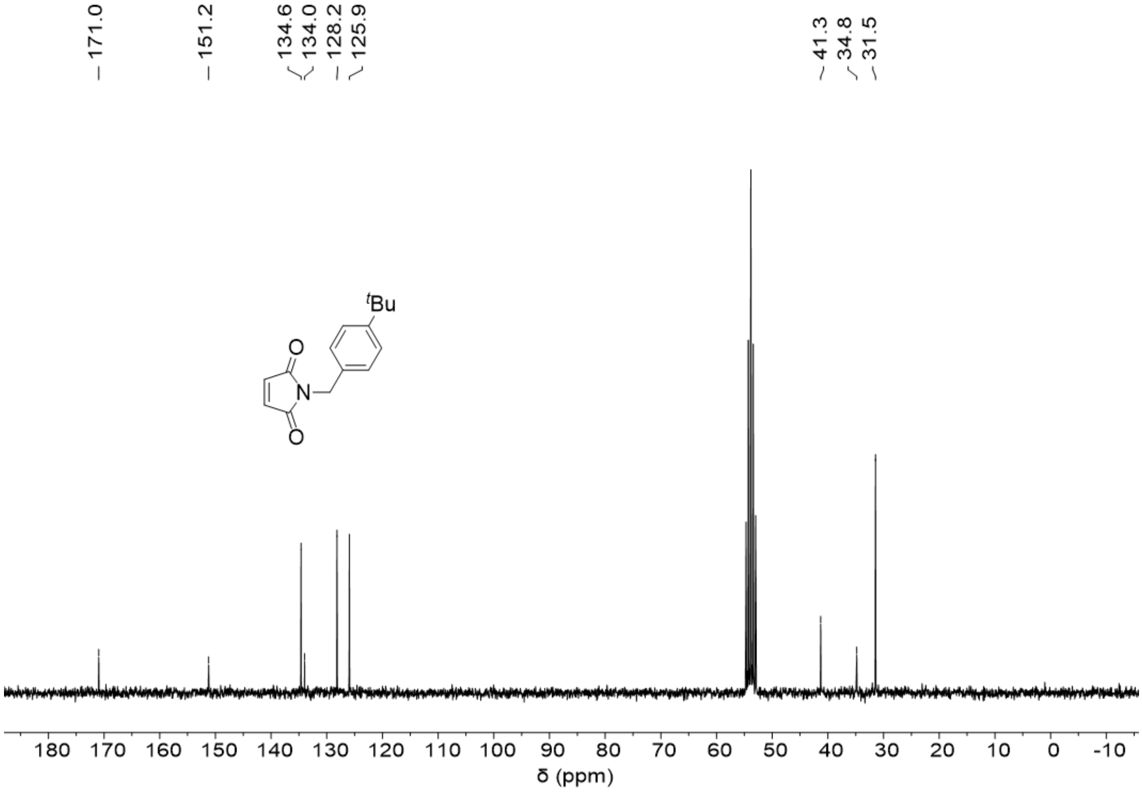

^{13}C NMR spectrum of *N*-(4-*tert*-butylbenzyl)maleimide (**2b**) (CD_2Cl_2, 63 MHz, 298 K).

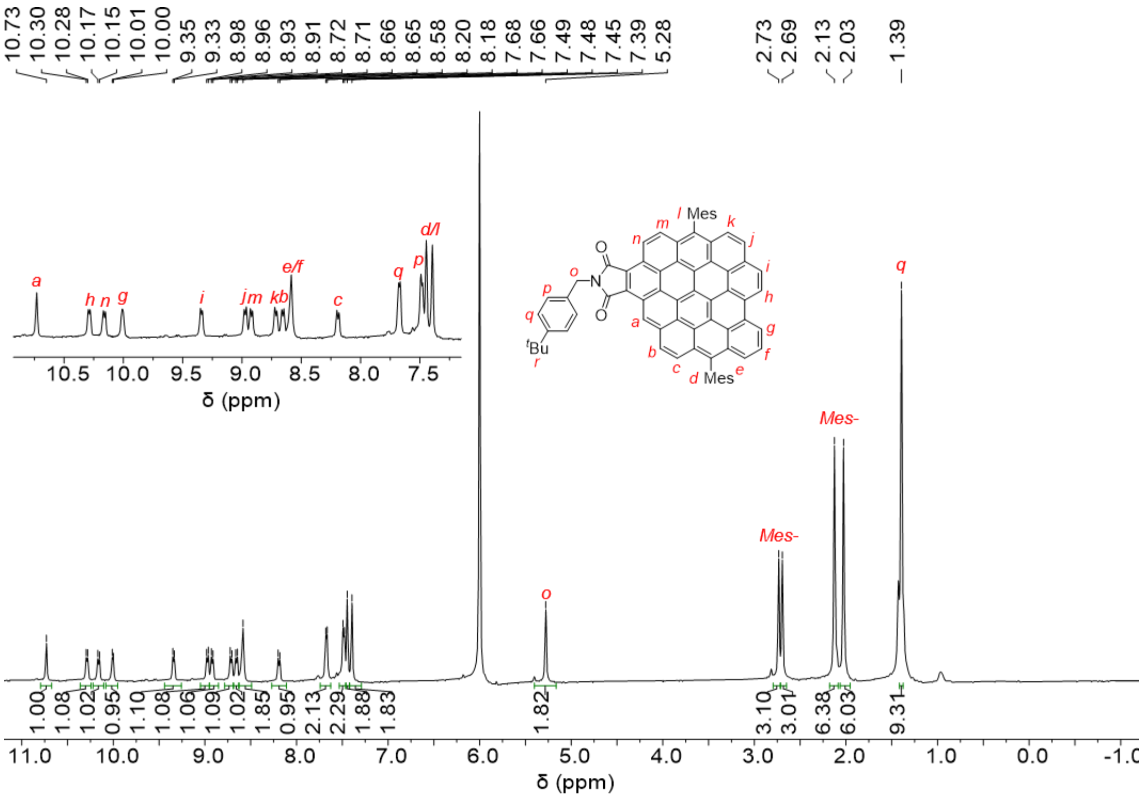

^{1}H NMR spectrum of mono-adduct **3b** ($C_2D_2Cl_4$, 500 MHz, 413 K).

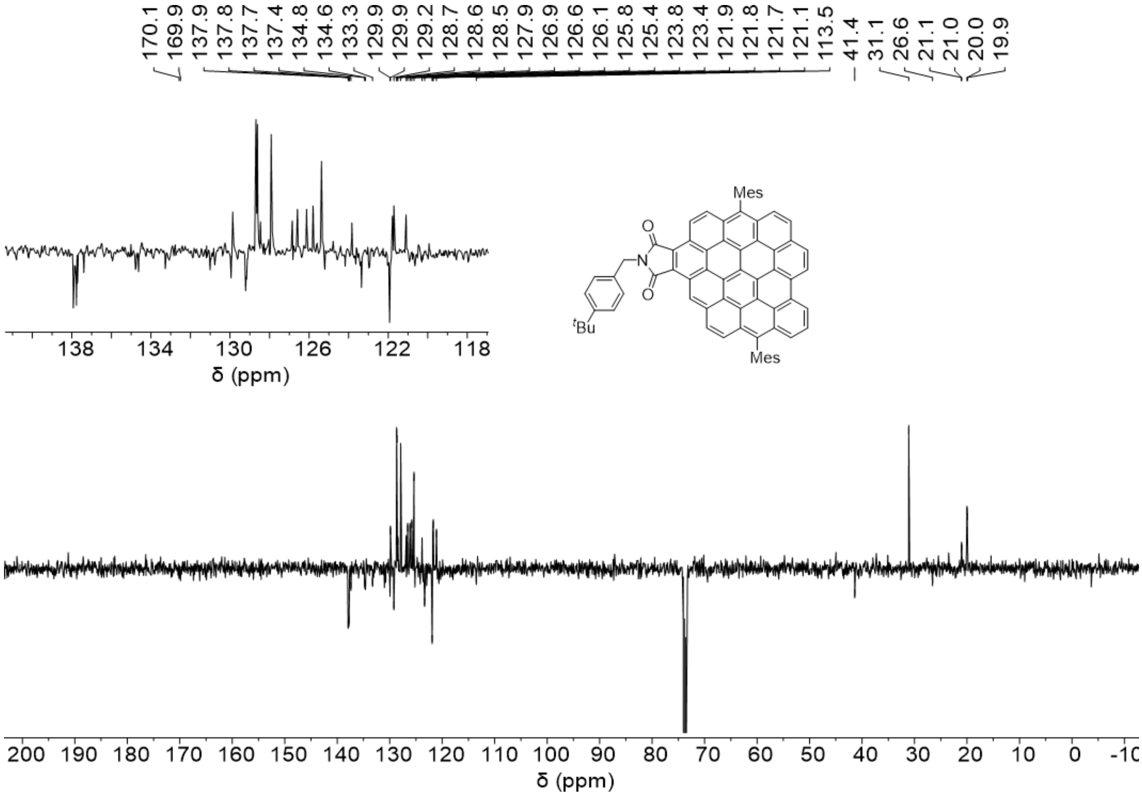

APT ^{13}C NMR of mono-adduct **3b** ($C_2D_2Cl_4$, 125 MHz, 413 K).

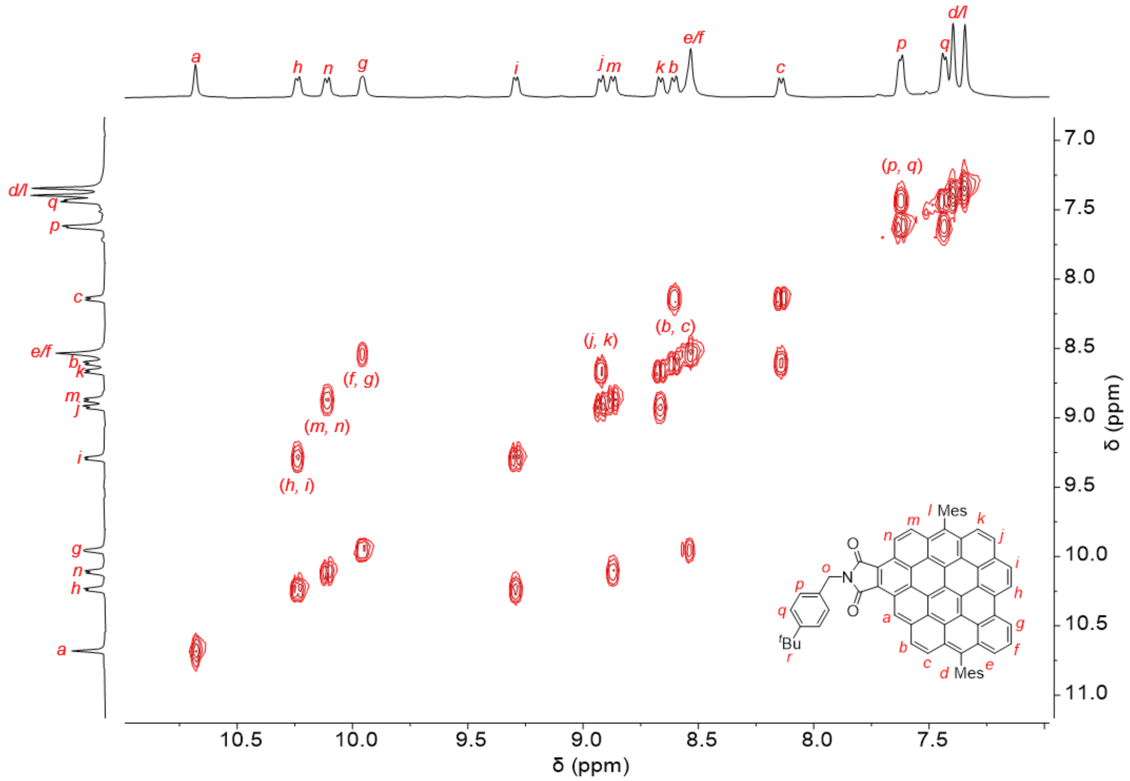

Aromatic region of ^{1}H,^{1}H-COSY spectrum of mono-adduct **3b** ($C_2D_2Cl_4$, 500 MHz, 413 K).

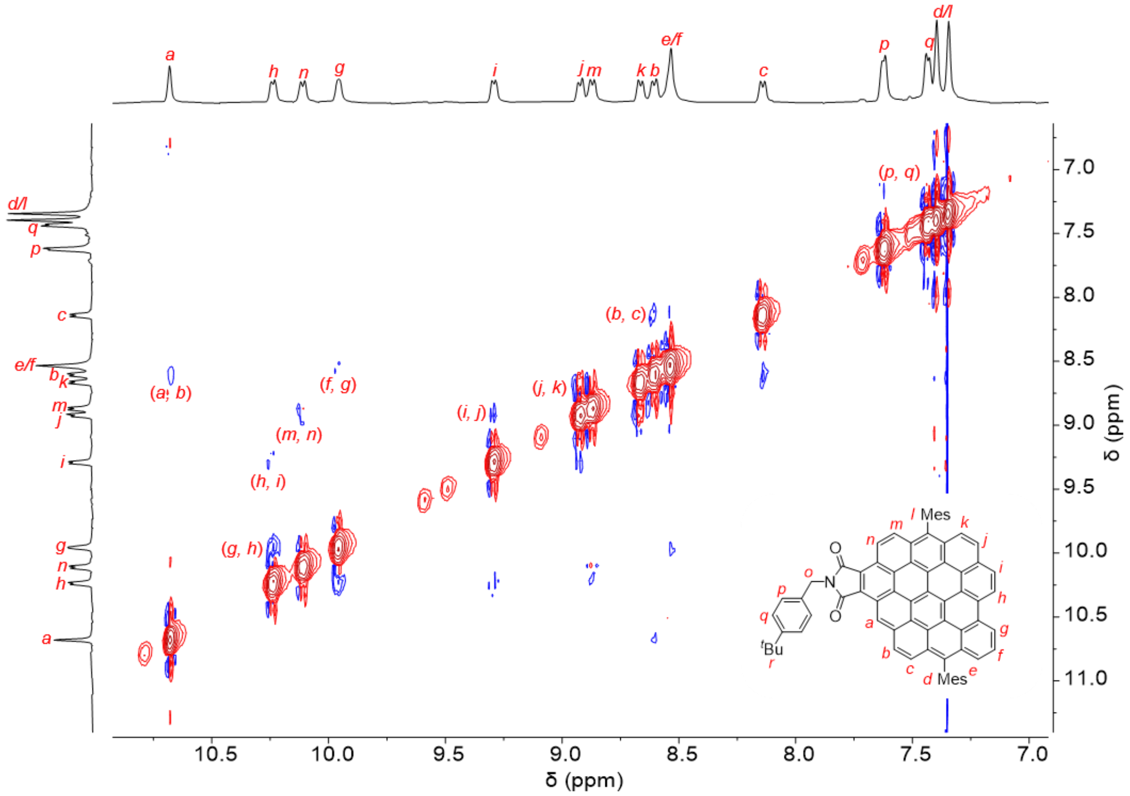

Aromatic region of ^{1}H,^{1}H-NOE spectrum of mono-adduct **3b** ($C_2D_2Cl_4$, 500 MHz, 413 K).

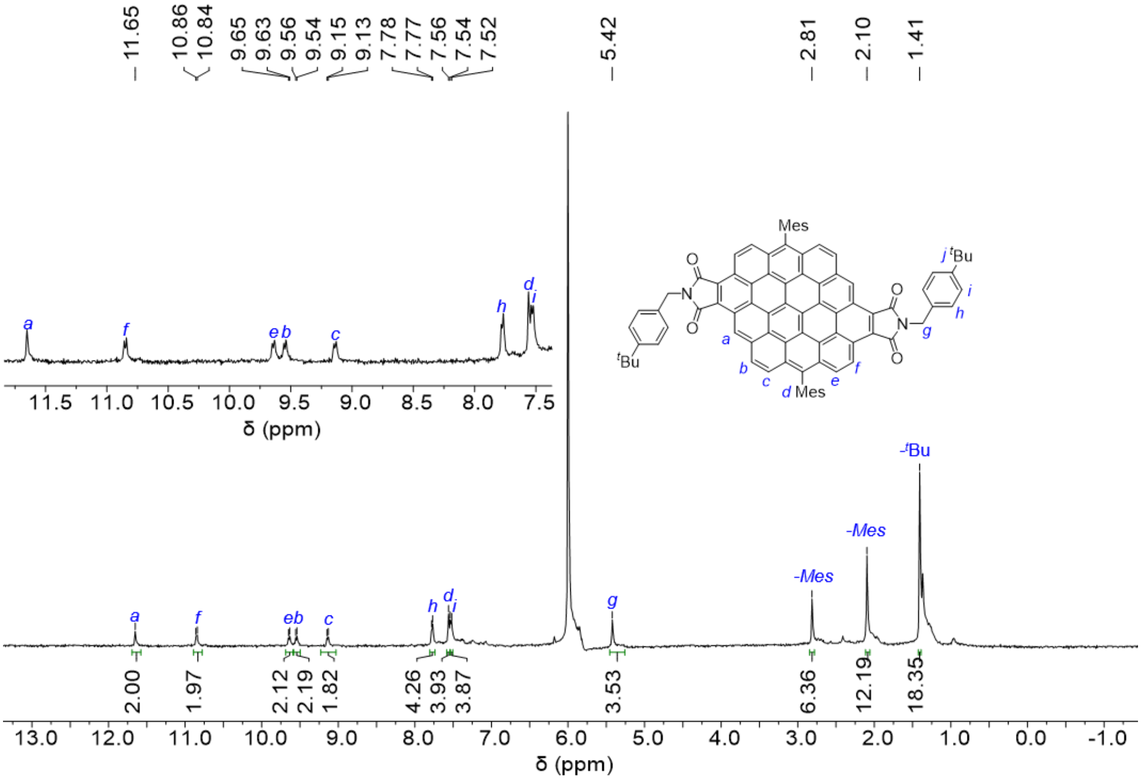

^{1}H NMR spectrum of di-adduct **4b** ($C_2D_2Cl_4$, 500 MHz, 413 K).

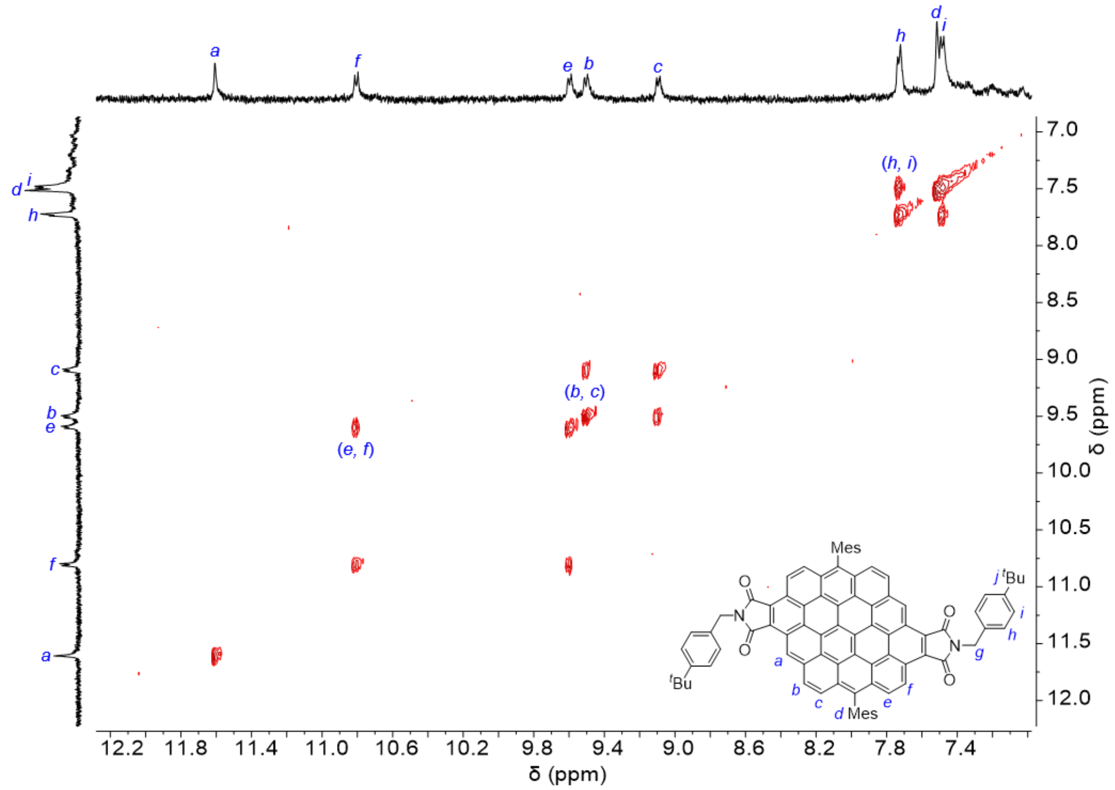

Aromatic region of $^{1}H,^{1}H$-COSY spectrum of di-adduct **4b** ($C_2D_2Cl_4$, 500 MHz, 413 K).

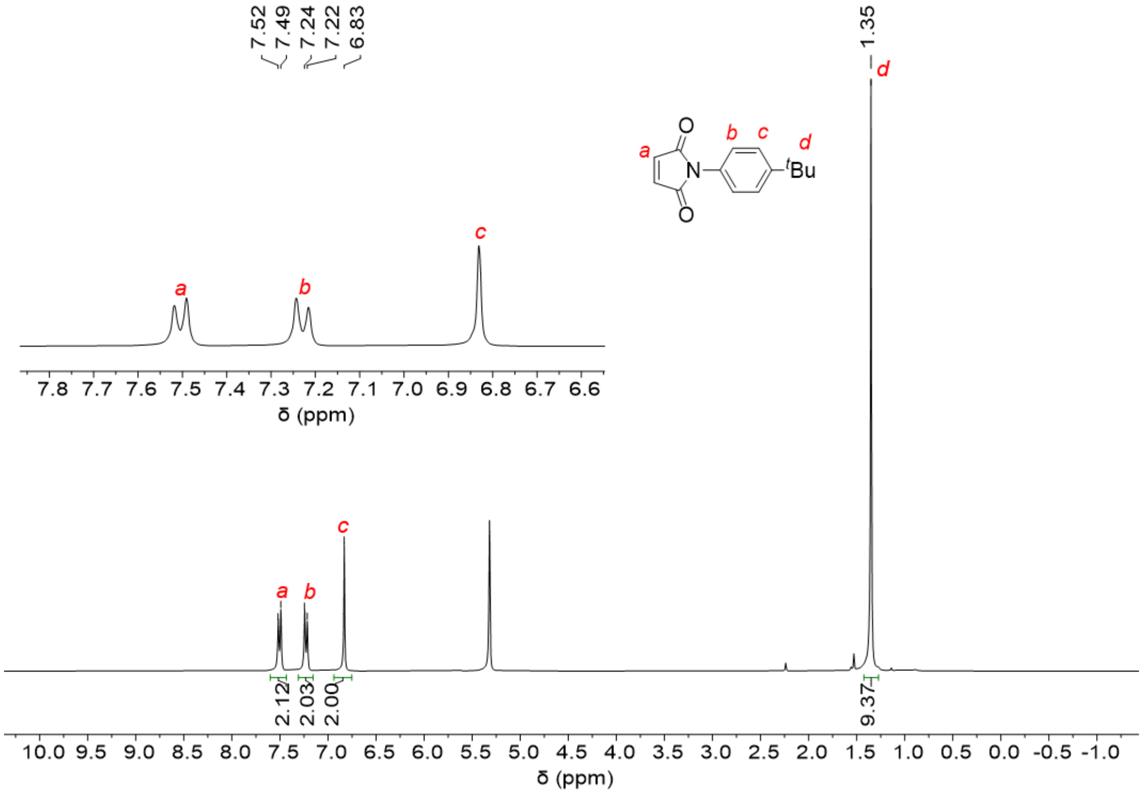

^{1}H NMR spectrum of *N*-(4-*tert*-butylphenyl)maleimide (**2c**) (CD_2Cl_2, 300 MHz, 298 K).

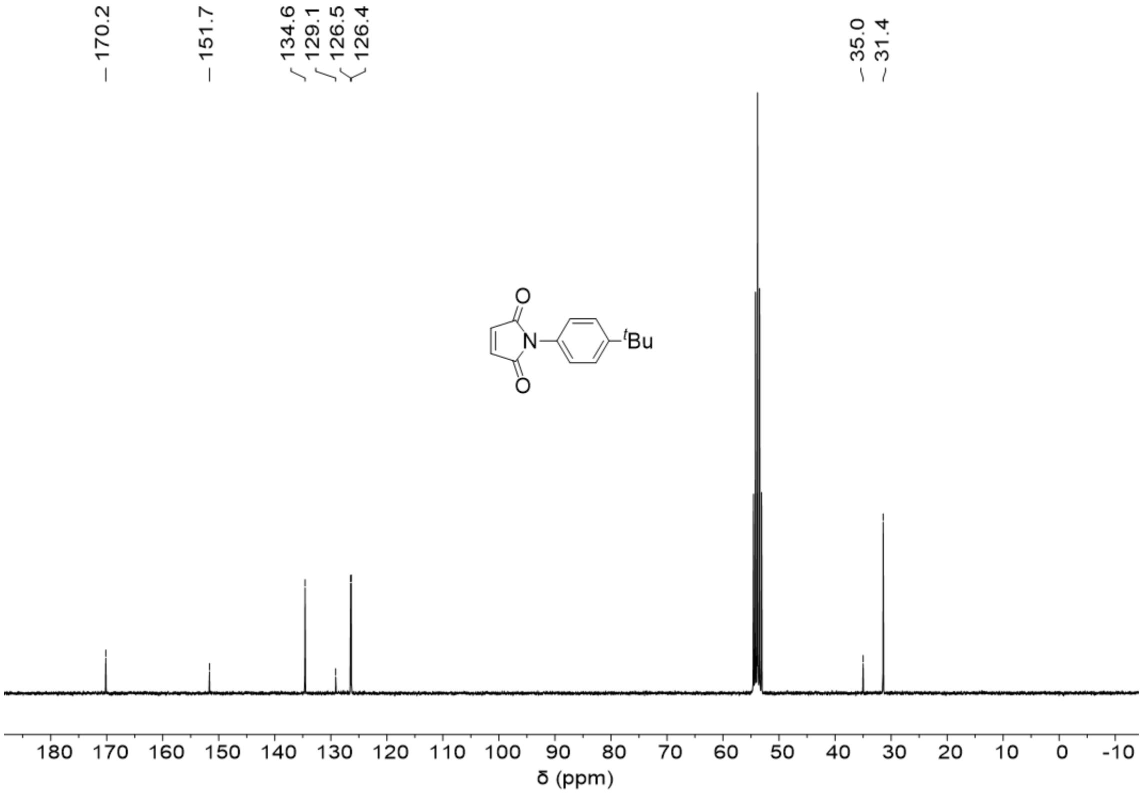

^{13}C NMR spectrum of *N*-(4-*tert*-butylphenyl)maleimide (**2c**) (CD_2Cl_2, 75 MHz, 298 K).

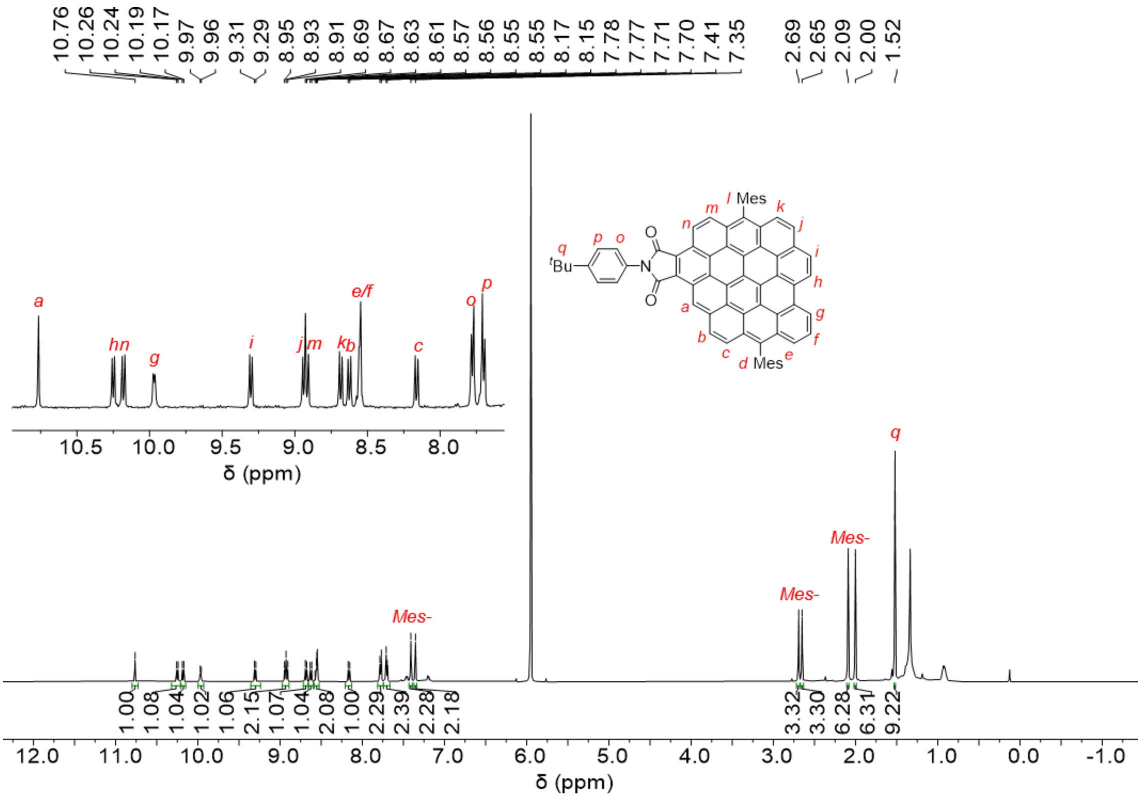

^{1}H NMR spectrum of mono-adduct **3c** ($C_2D_2Cl_4$, 500 MHz, 413 K).

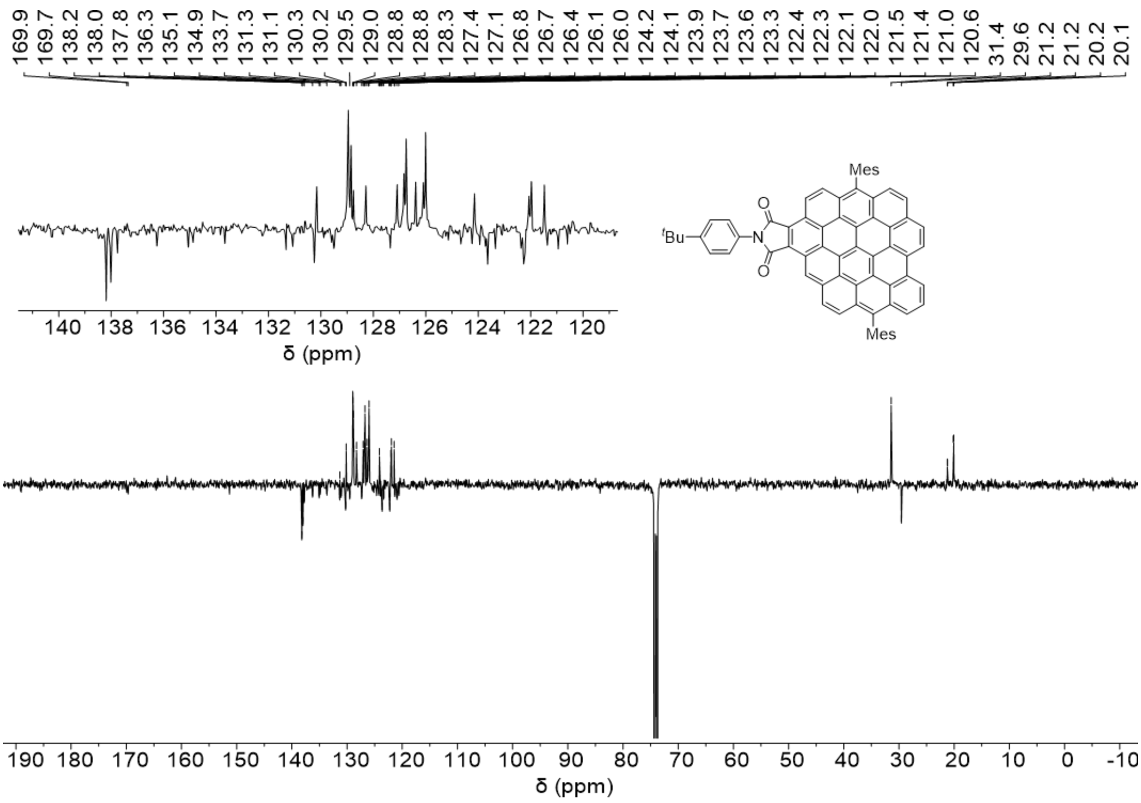

APT ^{13}C NMR of mono-adduct **3c** ($C_2D_2Cl_4$, 125 MHz, 413 K).

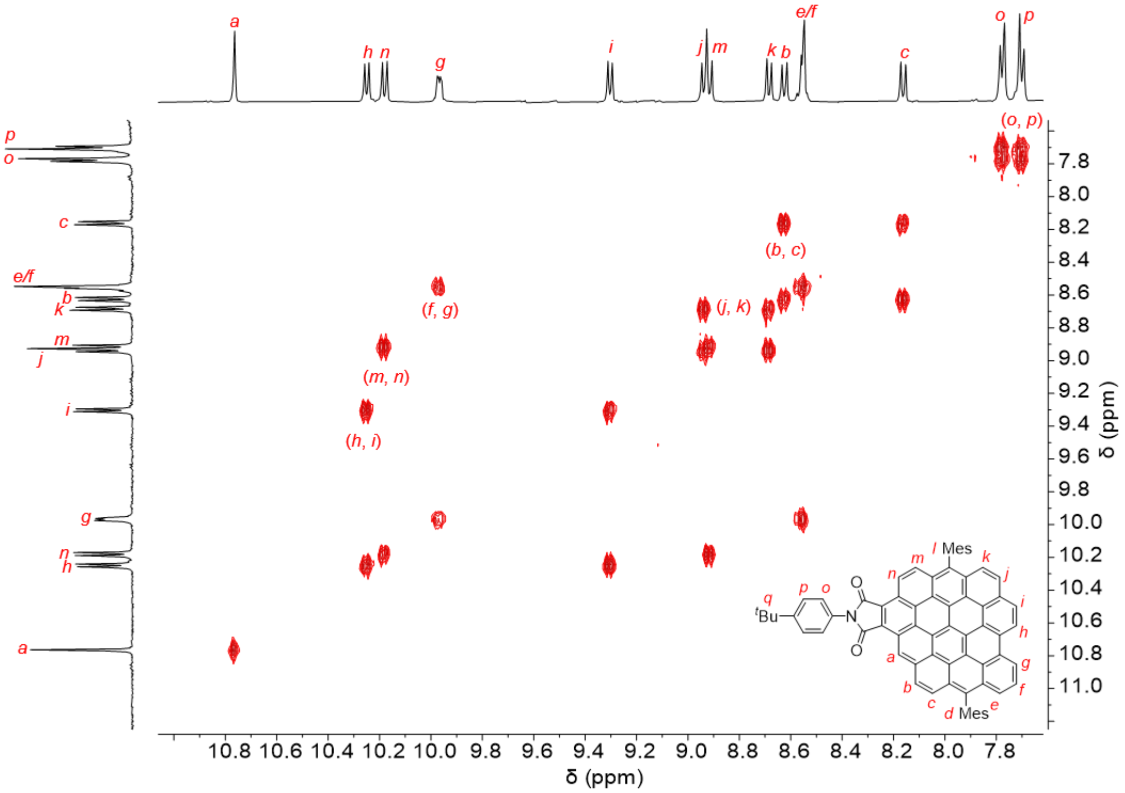

Aromatic region of ^{1}H,^{1}H-COSY spectrum of mono-adduct **3c** ($C_2D_2Cl_4$, 500 MHz, 413 K).

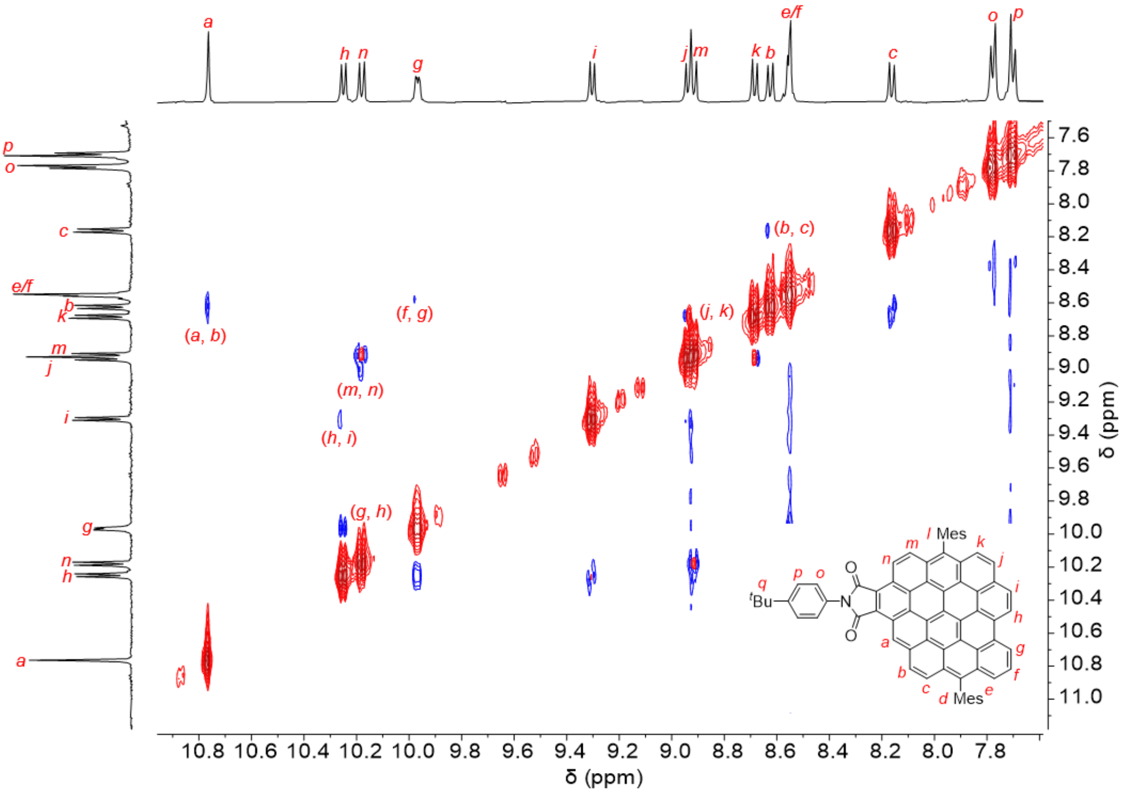

Aromatic region of ^{1}H,^{1}H-NOE spectrum of mono-adduct **3c** ($C_2D_2Cl_4$, 500 MHz, 413 K).
